# Motor Evoked Potential Warning Criteria in Supratentorial Surgery: A Scoping Review

**DOI:** 10.3390/cancers13112803

**Published:** 2021-06-04

**Authors:** Evridiki Asimakidou, Pablo Alvarez Abut, Andreas Raabe, Kathleen Seidel

**Affiliations:** 1 Department of Neurosurgery, Inselspital, Bern University Hospital, 3010 Bern, Switzerland; evridiki.asimakidou@students.unibe.ch (E.A.); pabloalvarezabut@gmail.com (P.A.A.); Andreas.Raabe@insel.ch (A.R.); 2 Department of Neurosurgery, Clínica 25 de Mayo, 7600 Mar del Plata, Argentina

**Keywords:** motor evoked potential, warning criteria, glioma surgery, aneurysm clipping, motor deficit, intraoperative monitoring, intraoperative neurophysiology

## Abstract

**Simple Summary:**

Motor evoked potential (MEP) alarm criteria may have an important impact on the preservation of motor function in supratentorial neurosurgical procedures. However, no consensus exists regarding the optimal cut-off values and interpretation of MEP signal changes. In addition, their performance as diagnostic and surrogate biomarkers has not been adequately investigated. The existing clinical studies that utilized alarm criteria are heterogeneous, rendering quantitative evidence synthesis problematic. In this study, we sought to summarize the pertinent literature using an emerging synthesis methodology, namely a scoping review. The objective was to assess the extent and range of available evidence, identifying research gaps, clarifying concepts, and providing insights for further research. Due to the heterogeneity of studies, we applied a descriptive approach, in particular by visualizing instead of pooling the data. A comprehensive overview of MEP warning criteria has not been provided yet, and therefore, our study should pave the way for future research.

**Abstract:**

During intraoperative monitoring of motor evoked potentials (MEP), heterogeneity across studies in terms of study populations, intraoperative settings, applied warning criteria, and outcome reporting exists. A scoping review of MEP warning criteria in supratentorial surgery was conducted in accordance with the Preferred Reporting Items for Systematic reviews and Meta-Analyses extension for Scoping Reviews (PRISMA-ScR). Sixty-eight studies fulfilled the eligibility criteria. The most commonly used alarm criteria were MEP signal loss, which was always a major warning sign, followed by amplitude reduction and threshold elevation. Irreversible MEP alterations were associated with a higher number of transient and persisting motor deficits compared with the reversible changes. In almost all studies, specificity and Negative Predictive Value (NPV) were high, while in most of them, sensitivity and Positive Predictive Value (PPV) were rather low or modest. Thus, the absence of an irreversible alteration may reassure the neurosurgeon that the patient will not suffer a motor deficit in the short-term and long-term follow-up. Further, MEPs perform well as surrogate markers, and reversible MEP deteriorations after successful intervention indicate motor function preservation postoperatively. However, in future studies, a consensus regarding the definitions of MEP alteration, critical duration of alterations, and outcome reporting should be determined.

## 1. Introduction

During supratentorial surgery, risk stratification and intraoperative guidance of the surgical strategy depend on various tools. Intraoperative monitoring of motor evoked potentials (MEPs) enables real-time assessment of functional integrity of motor pathways and has become a valuable adjunct in neurosurgical procedures [1,2]. Minimizing the risk of disabling motor deficits is the main factor during surgery in eloquent motor areas. At the same time, this constitutes the major challenge for the neurosurgeon, who aims to achieve the best possible surgical outcome, such as the maximal extent of tumor removal, without compromising the patient’s functional status.

Classical, intraoperative stimulation for MEP can be delivered through scalp electrodes (transcranial electrical stimulation, TES) or directly over the exposed motor cortex via strip electrodes (direct cortical stimulation, DCS). The responses are recorded from the target muscles (muscle MEPs) or (less frequent) with epidural electrodes (D wave) [3,4]. Intraoperative recording of muscle MEPs requires trains of stimuli to overcome the aesthetic inhibition of the lower motor neuron excitability by temporal and spatial summation of the excitatory postsynaptic potentials [5]. Stimulating scalp montages are derived from the 10/20 international system. Scalp stimulating arrays are placed at measured sites over the motor cortex to allow hemispheric stimulation (C3/Cz-1 and C4/Cz-1) or inter-hemispheric stimulation (C3/C4, C4/C3, C1/C2, and C2/C1) [6]. As classical stimulation intensity is applied slightly above the motor threshold, responses of several muscles can be recorded at the same time. Direct cortical and subcortical stimuli might be applied focal on the primary motor cortex or at the trajectory of the corticospinal tract (CST) and thus, elicit MEP in a few muscles of one anatomical territory [7,8].

Intraoperative MEP signal changes may result from an acutely disturbed nerve action potential conduction along the corticospinal axons because of compression, traction, ischemia, or mechanical injury [9]. However, MEP alterations may also be confounded by non-surgical factors. MEPs exhibit trial-to-trial variability and are susceptible to the effect of volatile anesthetic agents, neuromuscular blockade, systemic factors like hypotension and hypothermia, and focal factors like nerve conduction failure because of malpositioning [9]. Provided that non-surgical causes are excluded, MEP alterations should urge the surgical team to intervene or to stop in time while the impending neurological injury is still reversible.

Warning criteria represent a priori defined parameters. Optimally, they should alert the surgical team, and they prompt the implementation of corrective measures. Obviously, a false-negative reassurance will miss the neurological injury; however, a false-positive alarm may also indirectly harm the patient by stopping the surgery too early. The most common proposed MEP alarm criteria include the disappearance of MEP signal, amplitude reduction, threshold elevation, and morphology simplification [9,10,11]. Additional discussed warning criteria include latency increase [12,13], decrease in the Area Under the Curve (AUC) [14], and increase in potential width [15]. Further, different criteria are recommended for supratentorial surgery, compared to the brainstem, skull base, and spinal surgery [9]. Moreover, the magnitude of MEP change regarded as alarming varies substantially across neurosurgical centers and sometimes depends on previous institutional experience [16,17,18,19]. It becomes apparent that there is no consensus on the interpretation of MEP signal alterations and the selected cut-off values are often empirically derived [20,21].

The diagnostic accuracy of MEPs in supratentorial surgery for temporary and permanent postoperative motor deficits has not been adequately investigated, and the existing evidence provides controversial results [22,23]. Drawing overall conclusions is fraught with difficulty, as there is significant heterogeneity across primary studies in terms of methodological approach and reporting of outcomes. In light of all these considerations, we conducted a scoping review of MEP warning criteria in supratentorial neurosurgical procedures, including tumor, vascular, and epilepsy surgery. The objective was to assess the extent, range, and nature of primary studies that utilized intraoperative MEP warning criteria, summarize their findings within the context of postoperative motor outcomes, identify research gaps and provide implications for future research. Subsequently, we intended to perform a diagnostic accuracy analysis of MEPs as well as a correlation analysis between postoperative motor deficits and recovery of an intraoperative MEP alteration after successful interventions to investigate the value of MEPs as a surrogate marker.

## 2. Materials and Methods

The scoping review was conducted in accordance with the Preferred Reporting Items for Systematic reviews and Meta-Analyses extension for Scoping Reviews (PRISMA-ScR) [24] and was based on the methodological framework suggested by Arksey and O’Malley and refined by Levac et al. [25,26].

### 2.1. Search Strategy

The literature research was done by two independent researchers using the electronic databases PubMed (MEDLINE), Embase, Scopus, CINAHL, and the Cochrane Library. The included research articles ranged from the beginning of the databases until April 2021. There was no restriction on language. A detailed description of search terms and techniques is provided in Appendix A. The reference lists of retrieved articles and the sets of similar articles suggested by the database were screened in order to identify additional relevant citations. Additionally, the grey literature databases Open Grey, NTIS, British Library Direct Plus, York’s CRD, and Mednar were also searched.

### 2.2. Eligibility Criteria

Studies eligible for inclusion were all types of primary clinical studies, in which at least one MEP warning criterion was used intraoperatively in anesthetized patients during supratentorial surgery, including intrinsic brain tumors, metastases, aneurysms, vascular malformations, and other brain lesions, that are targets for epilepsy surgery. The utilized warning criteria had to be preoperatively (a priori) defined, and the authors had to report postoperative motor outcome data in conjunction with the intraoperative presence or absence of MEP alterations. MEPs had to be elicited by electrical stimulation and not transcranial magnetic stimulation. Our goal was to analyze MEP monitoring alarm criteria, but if D-wave recording or subcortical mapping were used as supplementary factors for warning signs, we included these aspects as well. However, we did not include studies solely with D-wave monitoring or mapping warning criteria without continuous muscle MEP monitoring. Studies with awake patients, infratentorial and spinal cord lesions were not included in our analysis unless the outcomes for asleep patients with supratentorial lesions were clearly described in a subgroup. In this case, only the data for supratentorial operations for anesthetized patients were extracted for further analysis. Studies with aneurysms located in arteries of the posterior circulation were included because the primary outcome of interest was the postoperative motor outcome and not ischemia.

### 2.3. Study Selection

The study selection was performed by two independent authors. Each author screened the titles and abstracts of all retrieved articles, defined a subset of relevant studies, and after full-text review, selected the eligible studies. The results of their individual search were compared, and a final list of eligible records was created. Some disagreements were resolved through discussion. If multiple publications from the same authors or overlapping study populations from the same institution were identified, the most recent paper was taken into consideration. The reason why specific articles did not meet the inclusion criteria can be found in Appendix B.

### 2.4. Data Extraction 

From each included study, the following data were extracted: authors, year of publication, study design, country, number of patients with MEP data, stimulation technique (transcranial electrical stimulation (TES), direct cortical stimulation (DCS), subcortical stimulation), and stimulation parameters, recorded muscles, MEP warning criterion/criteria, interventions in case of a warning sign, number of reversible and irreversible intraoperative MEP changes and number of patients with postoperative motor deficit immediately after surgery as well as during short-term and long-term follow-up. A pilot test of the data extraction protocol was initially performed with five citations and was afterward implemented for all included studies.

### 2.5. Data Analysis and Synthesis of Results

The extracted data were charted in tables with special emphasis on the number of patients with reversible or irreversible MEP changes who developed a postoperative motor deficit. A 2 × 2 contingency table was constructed for each study providing sufficient information to identify the true positive (TP), false positive (FP), false negative (FN), and true negative (TN) results. Subsequently, we performed a Diagnostic Test Accuracy (DTA) analysis of MEPs for postoperative motor deficits. Sensitivity, specificity, Positive Predictive Value (PPV), and Negative Predictive Value (NPV) were calculated using the RevMan calculator in the Review Manager software (RevMan, version 5.4) from the Cochrane Collaboration [27]. The forest plots displaying sensitivity, specificity, and the corresponding 95% Confidence Interval (CI) were generated for each study with the same software [27]. In order to visualize the values of diagnostic accuracy measures across all studies, heatmaps were constructed using MATLAB (version R2020b). The DTA analysis was divided into four sub-analyses and more specifically in the analysis of early-transient motor deficit (reported by authors as motor deficit immediately after surgery or at the day of the operation or resolved before the day of discharge), transient motor deficit (reported by authors as temporary or transient or present at discharge or short-term motor deficit), permanent motor deficit (reported by authors as permanent or persistent or long-term motor deficit) and all motor deficits regardless of the postoperative duration of the impairment. Given the differences in outcome reporting among the studies, this descriptive approach was deemed more appropriate than the use of the common cut-off time of 3 months to distinguish transient from permanent deficits. A postoperative motor deficit was defined as any new motor deficit or deterioration of an already compromised motor function with a decrease of ≥0.5 points on the Medical Research Council Scale (MRCS) or an increase of ≥0.5 points on the Modified Rankin Scale (mRS). In all sub-analyses, irreversible MEP changes according to the utilized warning criterion that did not recover until the end of the operation were considered as positive results, whereas reversible MEP changes and absence of MEP changes were considered as negative results. Detailed definitions for the DTA analysis are provided in Table S1. The DTA sub-analyses were performed separately for different stimulation modalities and warning criteria if it was possible to retrieve the relevant data from a primary study. Monitoring and mapping criteria were analyzed both separately and in combination, if applicable. We did not pool the data and did not undertake a meta-analysis of the results because of the heterogeneity in study populations, anesthetic regimens, stimulation techniques and parameters, recorded muscles, and utilized MEP warning criteria.

In addition to the DTA analysis, we carried out an analysis of the correlation between intraoperative MEP alterations that were reversed after successful intervention and new postoperative motor deficits. We sought to investigate the direction of association between these two variables in order to assess the significance of MEPs as surrogate endpoints. The calculations were performed based on the formulas and the methodology described by Holdefer et al. [28]. A 3 × 2 summary table was constructed for each study, and the proportion of reversible MEP changes after intraoperative intervention triggered by MEP warning criteria as well as the proportion of new motor deficits associated with MEP alterations were defined. All new motor deficits were included regardless of the postoperative duration. The correlation analysis was performed in R (version 4.0.2, R-project.org). The normality of the data was checked with a Shapiro–Wilk test, and Spearman’s rank correlation coefficients with corresponding *p*-values were computed. The plots were constructed using the R package ggplot2. Case reports were not included in the DTA analysis or in the correlation analysis.

## 3. Results

**Figure 1:**
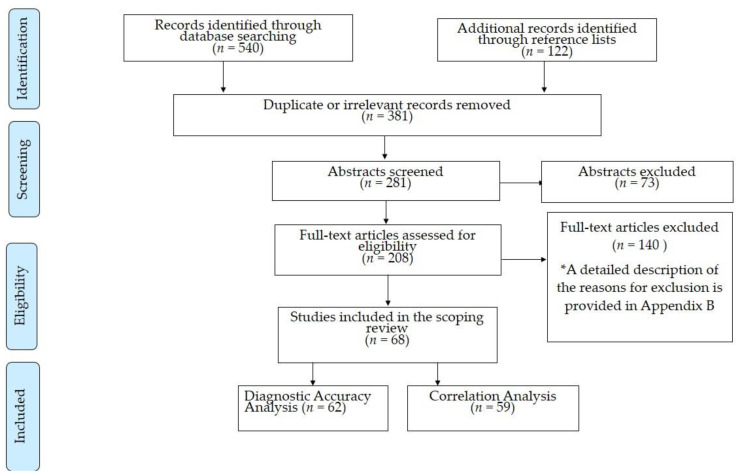
Flow diagram for study selection.

A total of 662 records were identified from the literature research. In particular, we extracted 540 references from electronic databases (204 from PubMed, 224 from Scopus, 63 from Embase, 25 from CINAHL, three from Cochrane Library, and 21 from grey literature databases), while the reference lists provided 122 additional citations. The titles were screened for relevance to our research question and for duplicate records. After exclusion of irrelevant or dual records, 281 abstracts were further screened, and subsequently, the full-texts of 208 articles were reviewed. Finally, 68 studies (31 studies for tumors and other brain lesions, two for epilepsy surgery, 28 for aneurysm clipping, five for endovascular aneurysm procedures, and two case reports) fulfilled the eligibility criteria and were included in our review. All of them were published in peer-reviewed journals, and no record from grey literature databases met the inclusion criteria. Figure 1 depicts the flow chart with the different phases of study selection. 

The included studies were 30 prospective and 28 retrospective case series, two case series with both prospective and retrospective design, six case series with unclear study design, and two case reports. The largest portion of evidence for tumors and other brain lesions was derived from Europe (especially Germany), whereas the main body of literature for aneurysm surgery consisted of studies from Asia (especially Japan, Korea, and China). An overview of MEP warning criteria utilized in supratentorial surgery and a summary of transient and permanent postoperative motor deficits in correlation with reversible and irreversible alarming MEP alterations for tumor surgery is provided in Table 1. The equivalent for vascular surgery can be found in Table 2. In all studies, MEP signal loss was considered a major warning sign. Additional information about the pathology of treated lesions, stimulation parameters, recorded muscles, and interventions following the appearance of warning criteria is provided in Table S2 in the Supplementary Material.

**Figure 2:**
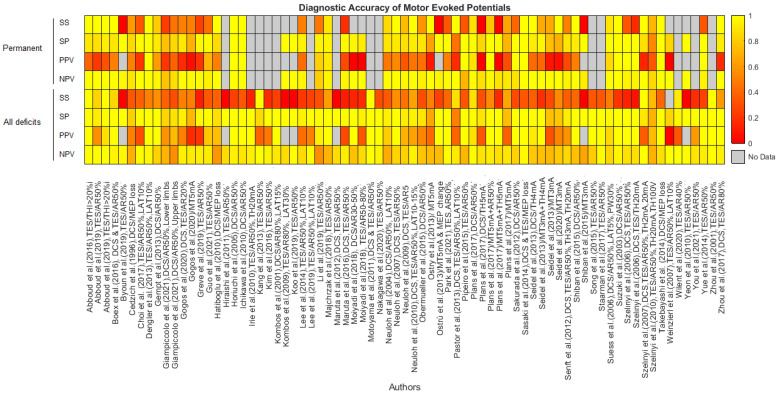
Heatmap depicting sensitivity (SS), specificity (SP), Positive Predictive Value (PPV), and Negative Predictive Value (NPV) estimates for permanent and all motor deficits (regardless of the time) across different studies. The scale ranges from 0 (red) to 1 (yellow). If the study did not provide sufficient data for the calculation of an estimate, the corresponding area is colored grey. Irreversible MEP changes were considered as positive results, whereas reversible MEP changes and the absence of MEP changes were considered as negative results.

**Figure 3:**
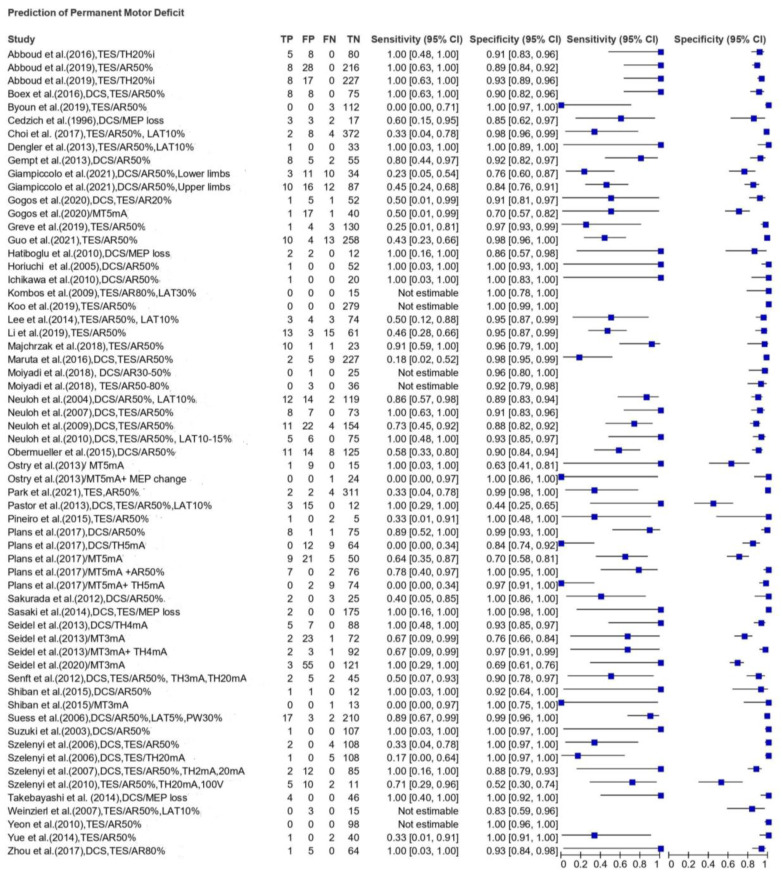
Forest plot of sensitivity and specificity estimates for permanent motor deficits AR: Amplitude criterion; DCS: Direct Cortical Stimulation; FN: False Negative; FP: False Positive; i: ipsilateral; LAT: Latency criterion; MEP: Motor Evoked Potential; MT: Motor Threshold/Mapping criterion; PW: Pulse Width Increase; TES: Transcranial Electrical Stimulation; TH: Threshold criterion; TN: True Negative; TP: True Positive.

**Figure 4:**
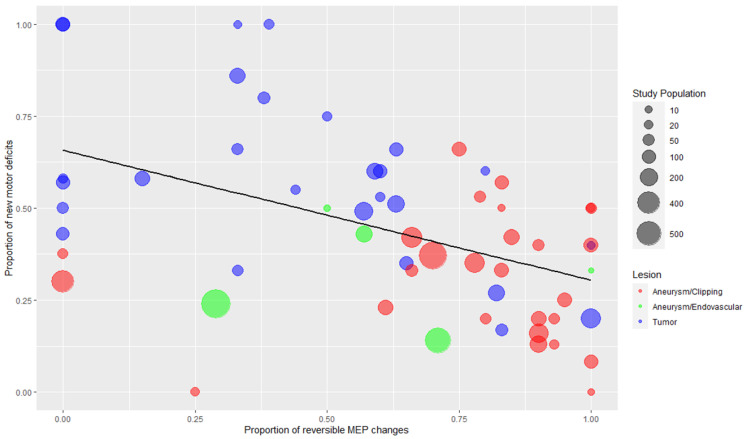
Bubble plot with regression line depicting the negative correlation between the proportion of reversible MEP changes and proportion of new motor deficits associated with MEP changes. Each dot represents one study, and the color corresponds to the type of the lesion (blue: tumor, red: aneurysm/clipping, green: aneurysm/endovascular procedures). The two studies of epilepsy surgery are depicted together with tumors (blue color). The third dimension added is the study population size, which is displayed by the size of each dot. Spearman’s rank correlation coefficient = −0.498 (*p* < 0.001).

The overall results of the DTA analysis are presented in a heatmap in Figure 2. The corresponding numerical values are described in detail in Tables S3–S6. Sensitivity and specificity estimates of MEPs regarding permanent postoperative motor deficits and their CIs are depicted in Figure 3. The forest plots for transient, early-transient, and all motor deficits can be found in Figures S1–S3, and separate heatmaps for permanent, transient, early-transient, and all postoperative motor deficits are provided in Figures S4–S7 in the Supplementary Material. The relative rates of MEP changes as well as the rates of reversible and irreversible MEP changes and permanent deficits are summarized for all studies in Table 3. Table S7 additionally depicts the total number of early-transient, transient, and permanent motor deficits in conjunction with MEP changes and the relative rate of all motor deficits in all studies. Figure 4 illustrates the results of the correlation analysis in a bubble plot, and the corresponding scatterplot can be found in Figure S8.

Overall, the results obtained from the data analysis suggest the following:

- Reversible MEP changes did not result in a postoperative motor deficit in most cases. If a motor deficit occurred, it was more frequently transient than permanent. Irreversible MEP changes were associated with a higher number of permanent than transient motor deficits;
- In almost all studies of the scoping review, specificity and NPV were high regardless of the timing of postoperative assessment. MEPs can reliably identify the true negative cases, and if no irreversible MEP alterations are observed, then it is not probable that the patient suffers a motor deficit immediately after surgery, in the short-term follow-up or in the long-term follow-up;
- Sensitivity and PPV varied across the studies and were rather low or modest in most of them, whereas some individual studies reported a 100% sensitivity and others a 100% PPV. The sensitivity estimates appeared to be higher for permanent motor deficits compared with the early-transient and transient deficits and for the threshold criterion compared with the amplitude criterion. PPV seemed to be higher for the prediction of any motor deficit regardless of the postoperative duration of the deficit. The low and modest values are impacted by the low prevalence of motor deficits;
- There was no remarkable difference in the diagnostic accuracy measures between TES and DCS in the included studies;
- In most cases, the combination of mapping and monitoring yielded higher PPV for all type of deficits compared with monitoring criteria alone;
- The CIs were narrow and indicated high precision of the specificity estimates, but the CIs of the sensitivity estimates were wide, implying greater uncertainty. The wider CIs for sensitivity are also attributed to the low incidence of postoperative deficits;
- The summary of events for each study demonstrated that the rate of postoperative motor deficits and intraoperative MEP changes is low. Regarding MEP changes, reversible alterations appeared to be more frequent than irreversible;
- The correlation analysis revealed a negative correlation between the proportion of reversible MEP changes and the proportion of new postoperative motor deficits associated with MEP changes (r_spearman_ = −0.498, *p* < 0.001).

## 4. Discussion

### 4.1. Type and Range of Available Evidence

This scoping review included 68 primary clinical studies and, more specifically, 31 studies for tumors and other brain lesions, two for epilepsy surgery, 28 for aneurysm clipping, five for endovascular aneurysm procedures, and two case reports. Except for the two case reports, all the studies were observational case series, either prospective (47%) or retrospective (44%), with a high number of patients. Small-scale studies with less than 20 patients were rare (12%), while non-randomized and randomized controlled trials (RCTs) were not identified. Obviously, the lack of RCTs from the body of the existing literature is attributed to ethical considerations that hinder the implementation of this study design [87]. As intraoperative monitoring of MEPs provides data that necessitates a rescue intervention, it would be unethical to ignore the intraoperative alarms in a group of patients or not to use them at all in operation within highly eloquent regions [88].

However, the clinical and methodological heterogeneity across the studies was remarkable, and the main sources of this heterogeneity were patients’ characteristics, intraoperative monitoring and mapping techniques, utilized MEP warning criteria, and outcome assessment. Undoubtedly, the varying study protocols and definitions render evidence synthesis quite challenging. This accounts for the relatively few reviews and meta-analyses of MEP warning criteria in supratentorial surgery, although some authors attempted to synthesize the existing research and estimate summary effects for vascular surgeries [89,90,91,92]. Due to those factors, we applied a descriptive approach using the systematic format of a scoping review, and we tried, in particular, visualizing instead of pooling the data.

### 4.2. Study Population and Type of Lesions

In many case series, children and adolescents were included in the study population. Except for patients in late adolescence, whose neuroanatomical and neurophysiological characteristics do not differ substantially from adults, the inclusion of young patients may confound the results. Due to incomplete myelination, modifications of intraoperative neurophysiological techniques might be necessary [93]. Especially in children younger than 10 years old, longer stimulating pulse trains and higher stimulation thresholds might be needed to elicit an MEP response [94,95]. However, the evidence regarding the optimal stimulation parameters in younger ages is scarce. It remains unclear if MEP warning criteria that are commonly used in supratentorial surgery of adults are equal in the pediatric population.

Similar MEP warning criteria were applied in surgeries for a broad spectrum of brain lesions, such as intrinsic glioma of different grades, extrinsic metastases, vascular malformations, cortical dysplasia, and aneurysms located in different arteries. Because of the obvious different surgical strategies and approaches, we did analyze studies for tumor surgeries separately from them for aneurysm surgeries (Table 1 and Table 2, Figure 4). During tumor surgeries, additional warning criteria provided by subcortical mapping have been suggested by 12% of the included studies, and this aspect will be discussed in detail below in the paragraph “The mapping-monitoring crosstalk and the warning sign hierarchy”. Aneurysm surgeries presented a higher tendency of reversible MEP changes than tumor surgeries, which will be discussed further in the paragraph “MEPs as surrogate markers”.

Concerning tumor location, Abboud et al. [33] observed a significant correlation of postoperative motor deterioration with tumor location in the insula. This fact might be attributed to the higher incidence of vascular injury during insular glioma surgery [30]. Krieg et al. [96] found a statistically significant increase in postoperative motor deficits in cases where the tumor was located in the precentral cortex compared with the postcentral and anterior frontal cortex. During resection of tumors located in the precentral gyrus, MEP monitoring might be inferior to mapping methods as either the stimulating electrode interferes with the surgical approach or the MEP might be generated in the corticospinal tract distal to the resected area [97]. Giampiccolo et al. [29] performed a lesion analysis, which showed that motor-evoked potential-related long-term motor deficits were associated with direct or ischaemic damage to the corticospinal tract, whereas muscle motor-evoked potential-unrelated deficits occurred when supplementary motor areas were resected in conjunction with dorsal premotor regions and the anterior cingulate. This important observation illustrates the fact that MEP represents the integrity of the primary motor cortex (M1) and the corticospinal tract but not associative motor areas.

### 4.3. Stimulation Techniques and Parameters

Continuous MEP monitoring was performed either with TES (via scalp electrodes) or DCS (electrodes directly placed on the precentral gyrus), but many authors used both stimulation techniques simultaneously or applied them in subgroups of patients in their series. The high-frequency multipulse stimulation technique introduced by Taniguchi et al. [5] was applied in all studies, but there were differences in stimulation parameters, which were more apparent in stimulation intensity. The stimulation intensity has special importance when interpreting the amplitude criterion, as MEP exhibits trial-to-trial variability, and MEP amplitude alterations may be caused by non-surgical factors [9]. There has been much debate regarding the use of TES in supratentorial surgery because TES induces less focal stimulation, and at high intensity, the activation site might be located deeper than the actual level of the lesion [17,77,98]. Rothwell et al. [99] stated that strong stimulation currents may activate the CST even at the foramen magnum. Hence, depending on the area of interest, there might be the risk of stimulating the white matter more caudally than the site of neurological damage, leading to a higher rate of false-negative results. Furthermore, loss of cerebrospinal fluid after dural opening leads to brain shift and subdural air accumulation. That fact may interfere with the reliability and evaluation of TES-MEP warning criteria. Further, TES may cause a higher rate of patient movement [100]. Because of these drawbacks, many neurosurgeons opt for DCS, which needs lower stimulation intensities and allows a focal and superficial stimulation of corticospinal neurons [8]. Nonetheless, DCS is not applicable in patients with scar tissue from previous operations. It may also interfere with MEP monitoring due to electrode dislocation on the cortex [101]. Szelényi et al. reported that TES and DCS do not differ in their ability to detect an impending neurological injury. Both paradigms may be alternatively applied during the same surgical procedure, provided if lateral TES montages are not used, and near-threshold stimulation intensities are applied [77]. Our scoping review supports this observation if applied as a general rule. However, a more thorough comparison between the two stimulation modalities depending on the type of surgery, pathology, and tumor location (especially in the precentral area) would be clinically meaningful and may contribute to an optimized implementation of warning criteria.

The inclusion criterion for our review was the use of MEP alarm criteria during continuous MEP monitoring, eventually complemented by a secondary test such as subcortical mapping. Consequently, studies, which applied mapping techniques (such as Penfield or high-frequency stimulation) without MEP monitoring were excluded. In all included studies, the high-frequency multipulse stimulation technique was utilized for subcortical motor mapping, and the stimulation parameters were similar to the ones in MEP monitoring. For subcortical mapping, monopolar cathodal stimulation was applied in all studies except for the study by Kombos et al. [49], who used anodal stimulation. In a comparative study by Shiban et al. [102], the authors pointed out that cathodal stimulation was superior and lower stimulation intensities were required. Gogos et al. [30] utilized bipolar stimulation in a subset of 20 patients in addition to monopolar stimulation and reported that bipolar stimulation elicited MEPs in only 30% of patients as opposed to the monopolar stimulation, which identified the descending motor tracts in 86.4% of patients. These results are in concordance with the findings of Szelényi et al. [103], who concluded that monopolar cathodal stimulation is more efficient than bipolar cathodal stimulation in subcortical motor mapping. Applying the short train monopolar paradigm, the current spreads radially towards a distal reference electrode [104]. This pattern of electric current spreading enables the estimation of the distance between stimulation point and CST based on the rule that 1 mA increase in subcortical stimulation threshold corresponds to 1 mm increase in the distance towards the CST. Up to now, no definitive statement on this relationship is possible; however, the vague rule of thumb “1 mA correlates to 1 mm” is increasingly used when performing subcortical short train monopolar stimulation with five 0.5 ms cathodal constant-current pulses. Three groups performed continuous (dynamic) subcortical mapping, with two of them integrating the monopolar stimulation probe in the surgical suction device [32,35] and one of them in the ultrasonic aspirator [39]. In the remaining studies, subcortical mapping was performed intermittently with a handheld probe. Allowing an uninterrupted and procedure-driven stimulation [32,105], continuous dynamic mapping via a surgical instrument may improve awareness of the intraoperative conditions and may facilitate immediate reaction to warning signs, but more studies addressing this aspect are needed to make a definitive statement.

### 4.4. The Spectrum of MEP Warning Criteria

The utilized warning criteria varied across the included studies, but in all cases, the disappearance of MEP signal was considered as a major alarming sign that required re-assessment of intraoperative settings and an adjustment of surgical strategy. Apart from MEP loss, the most commonly used warning criteria were amplitude reduction and threshold elevation with cut-off values that differed among the authors. Notably, a >50% amplitude reduction was regarded in the vast majority of studies as a significant change, although other magnitudes like >80% and >20% have also been applied [10,11,30,37,49]. Threshold cut-off values that have been used during TES were >100 V or >20 mA [16,45,48,75,77] and during DCS >3 mA [45], ≥4 mA [8,32], and ≥5 mA [36]. Interestingly, Ostry et al. [42] used during supratentorial tumor surgery a threshold increase >2 mA not as an indicator for surgical intervention but for the performance of subcortical mapping. The definition of a minimum MEP amplitude to be monitored varied significantly among the papers from 10 to 100 μV with an average of 30–50 μV [8,10,11,29,32,33,35,38,42,48,53,66,73,74,76,78]. However, in many, it was not clearly defined.

Recently, Abboud et al. [17] introduced a novel threshold criterion and suggested that a TES threshold increase on the affected side of more than 20% beyond the percentage increase on the unaffected side should be considered as a warning sign. In contrast to the conventional approach of a threshold increase beyond the baseline level, this modification incorporates changes on the unaffected side, which can serve as a negative control for MEP alterations caused by factors other than damage to the CST and highlights the significance of bilateral MEP monitoring. Indeed, the results in their series were highly promising, as none of the patients without threshold increase greater than 20% beyond the unaffected side suffered a postoperative motor deficit, but further studies are needed for the definite establishment of the novel threshold criterion. Latency increase has never been used as a sole criterion in supratentorial surgery and was always an adjunct to the amplitude criterion. A significant latency increase not accompanied by a consistent amplitude decrease was unusual. This might be obvious, as latency shift would indicate a demyelination process, which is not an expected acute type of injury in supratentorial surgery. Morphology simplification is also a poorly studied warning criterion that remains controversial and may be susceptible to subjectivity.

A significant flaw was that the majority of authors did not report the duration of reversible changes or if they applied a threshold for the duration, above which duration the alteration was considered as irreversible. This clarification is important, as a longer duration of the reversible MEP alteration might explain why some patients develop a postoperative motor deficit despite the successful MEP recovery after the intervention while others do not. Krieg et al. [96] noted that the mean duration of amplitude reduction and latency increase was significantly higher in patients with permanent motor deficit than those with no deficit. In aneurysm surgery, Li et al. [61] proposed 13 min as the cut-off duration of MEP deterioration for higher risk of ischemic damage, while Guo et al. [56] suggested 8.5 min and Kameda et al. [59] an even shorter duration of 5 min. The determination of a critical threshold to delineate the duration above which an MEP change should be regarded as irreversible and is associated with a higher risk for postoperative motor deficit would guide the neurosurgeon more efficiently and is worth being investigated. Similarly, the borderline between MEP amplitude reduction and MEP loss is still vague, as the maximal stimulation-intensity value has not been defined in many studies.

### 4.5. The Mapping-Monitoring “Crosstalk” and the Warning Sign Hierarchy

Regarding assistant mapping warning criteria, monopolar cathodal subcortical motor thresholds (MT) ≤5 mA and ≤3 mA were the most widely used thresholds that provided an alarming sign for proximity to CST. It has been suggested that the critical MTs might be even lower, but the exact threshold has not been identified yet [8].

As mentioned above, this scoping review included only those subcortical mapping studies that utilized mapping warning criteria combined with MEP monitoring criteria. Due to methodological reasons, subcortical mapping studies, which performed a post-hoc analysis to investigate the critical MT (without using an a priori defined MT as a surrogate to stop resection), studies that did not correlate motor outcome exactly to intraoperative alarm criteria or did not describe precisely the criteria when combining subcortical mapping with continuous MEP monitoring, were excluded [106,107,108,109,110,111].

Mapping and monitoring warning signs may present a hierarchical “crosstalk” between them [8]. Each method has distinct shortcomings and limitations, and their simultaneous use during surgery may lead to mutual reinforcement. MEP monitoring can assess the functional integrity of the primary motor pathways in real-time and detect potential damage caused by mechanical or vascular injury, providing a trigger for actions to reverse it. Nevertheless, no guarantee is provided that the motor pathway has not already been irreversibly damaged by the time the alarming sign occurs. In other words, the warning criterion may appear after the critical event, and hence, its value is undermined by the eventually irreversible nature of the damage. This fact is also illustrated in Table 3 and in Figure 4. During tumor compared to vascular surgery, a higher rate of irreversible compared to reversible MEP alterations was described. The reason might be the different patterns of injury, especially mechanically induced injury during tumor surgery. If the surgeon injures fibers of the CST, there might be no way back to reverse the damage.

Contrariwise, the mission of subcortical mapping is to localize the motor tracts and provide information about their distance from the operation site. A warning sign obtained from mapping denotes proximity to CST and not functional compromise of the CST. Subcortical motor mapping warns about an impending neurologic injury that has not necessarily taken place but is likely to occur if surgical maneuvers are further continued. Therefore, the surgeon is aware of working close to eloquent structures, meaning that meticulous maneuvers or reappraisal of surgical strategy might be necessary. For mechanical alteration of the CST, the mapping warning signs seem to precede those from monitoring, which may occur at a later moment with low mapping thresholds [8]. However, motor mapping is limited by its inability to detect remote vascular injury, critical end-artery blood supply, and ischemia due to brain retraction that can be detected by MEP monitoring [8]. The advantages of this combinatorial approach with these two neurophysiological techniques during tumor surgery came into focus during the past few years [8,30,32,35,36,39,49,108,109,110,111]. Our DTA analysis (Figure 2 and Figure 3) indicated that their combination results in a more powerful tool. Yet, more evidence is needed to confirm that the combination may achieve the ultimate goal of maximizing resection and minimizing debilitating motor deficits.

### 4.6. Different Patterns of Injury-Neurophysiological and Neurosurgical Considerations

Especially during resection of motor eloquent tumors, permanent motor deficits might be caused by different patterns of injury: direct (mechanical) injury of the primary motor cortex or the corticospinal tract, ischemia due to coagulation of critical perforator arteries, or lesion of multiple supplementary motor areas. In our scoping review, 54.8% of the included tumor papers did differentiate between injury patterns. If considering the described cases of permanent motor deficits, seven groups [30,38,39,40,44,47,51] described exclusive vascular injury and one group [55] sole mechanical injury as a cause. Nine groups [8,15,23,32,36,41,46,48,50] did observe different injury patterns in their tumor patients with permanent motor deficits with different ratios of vascular, mechanical, or other causes (see also Table S8 in the Supplementary Material).

In our own initial series of 100 motor eloquent tumors, we did describe five patients (5%) with a postoperative new or worsened motor deficit at three-month follow-up consultation [8]. In all five patients, DCS MEP monitoring alterations were documented (two sudden irreversible threshold increases and three sudden irreversible MEP losses). Of these five patients, two had ischemic vascular lesions, and three had mechanical CST damage. After the introduction of the dynamic mapping method (continuous stimulation via the surgical suction device), the permanent motor deficit rate was 3%, with direct mechanical injury in three of these patients (1.7%) [32]. In our series, DCS MEP alterations did occur abruptly, but they could be influenced in 60% of cases [8]. Further, the stability of DCS-MEPs did provide real-time feedback about the functional integrity of the CST and supported us to continue tumor removal at even very low mapping thresholds. This observation is supported by the recent publication of Gogos et al. [30], where the senior author M. Berger reports in his previous series of 700 motor mapping cases applying bipolar mapping that 41% of permanent motor deficits had to been attributed to “direct transgression of the motor system” [112]. The authors even concluded that adding monopolar (short train) stimulation and MEP monitoring as an additional neurophysiological tool could significantly reduce permanent motor deficits within the present series being reported at 3.4%. Giampiccolo et al. [29] observed MEP-related deficits in vascular territories (insular cortex and post-central gyrus) and anatomical territories (internal capsule and precentral gyrus) of the CST. However, they also described MEP-unrelated motor deficits in cases of SMA resection in conjunction with damage to the dorsal premotor and anterior cingulate cortex. Finally, they concluded that MEP drop predicts a permanent, severe motor deficit, which is associated with disconnection of the CST, and they did support MEP monitoring as an important neurophysiological marker.

As already discussed, MEP monitoring may detect different types of vascular damage such as direct vascular damage or injury of perforating vessels, critical end-artery blood supply (for example, in the lenticulostriate territory) due to hypoperfusion, and ischemia due to brain retraction. Those different mechanisms might explain why some MEP alterations can be reversed and others not. This fact is also in accordance with our findings in this scoping review (Table 3 and Figure 4). In the included papers of aneurysm surgery, a higher rate of reversible MEP changes attributed to temporary clipping (mean rate 16.2% of all temporary clipping cases corresponding to 78% of all MEP changes during temporary clipping) compared to irreversible MEP changes (mean rate 4.6%) was described, which demonstrates that MEP monitoring may successfully guide temporary clipping before definitive aneurysm repair.

Non-surgical factors, such as global or local hypoperfusion, may also affect the MEP amplitude. The autoregulation range of mean arterial pressure (MAP) varies from as low as 55 mmHg to rarely, as high as 113 mmHg [113]. However, the pathology itself or the clinical diseases of the patient may affect the capability of autoregulation, and already small MAP alterations might not be tolerated. In general, it has been described that the MEP amplitude starts to decrease if the cerebral blood flow falls below the threshold of 16 mL/min/100 g [114]. In those cases, raising the intraoperative blood pressure could restore blood flow and consequently MEP amplitudes [7].

### 4.7. MEP Warning Criteria and Postoperative Motor Deficit

The DTA analysis revealed that irreversible alterations of MEPs have high specificity and NPV. Sensitivity and PPV varied across the studies, and definite conclusions cannot be drawn, although in most studies, they tended to be low or moderate. This suggests that MEPs cannot always reliably detect the true positive cases. For intraoperative decision-making, an important aspect is the confidence in the test’s ability to distinguish patients who are likely to develop a motor deficit from those who are not, and in this regard, the high NPV of MEPs is meaningful [115]. In other words, if MEPs remain stable or an MEP change signified by a warning criterion is successfully reversed, then the M1 and the CST are expected to be intact after surgery. Stable or reversed MEPs may reassure the surgeon to continue and, in tumor surgery, achieve a higher extent of tumor resection. If an irreversible MEP change occurs, it does not mean per se that the patient will suffer a new motor impairment, although it is highly possible. Skepticism about false positive alarms and putting the alarm in the right context are important. The ensuing compromise of the surgical goal is reasonable. False alarms may indirectly harm the patient by stopping the surgery. However, too early termination of the surgery may be compensated through a reoperation, whereas a debilitating neurological injury cannot.

Nonetheless, the low prevalence of postoperative deficits has a significant impact on the PPV and may account for the observed low values. Thus, even minor test errors have a considerable effect on the performance of the test. This signifies that a refinement of the alarm criteria cut-off values may not significantly upgrade the PPV. In view of this limitation, the focus should be moved to a multimodal approach and, more specifically, to the combination of MEP monitoring with other intraoperative modalities such as subcortical mapping (see paragraph “*The warning sign hierarchy*”) and intraoperative imaging. Our DTA analysis provides implications for this perspective. The combination of monitoring and mapping criteria seemed to yield higher PPV estimates in tumor surgery. In the future, artificial intelligence and machine learning algorithms may markedly contribute to a better outcome prediction, counterbalancing the interrater variability and the inherent subjectivity of MEP evaluation. However, intraoperative decision-making based on MEP alarms should not be regarded as a rigidly mechanistic process. Thus, the neurophysiologist’s/neurosurgeon’s contextualization and intraoperative judgment are indispensable.

Secondary postoperative events such as delayed ischemia due to vasospasm, hemorrhage, and edema may lead to motor deficits that will not be detected intraoperatively with MEP monitoring. Therefore, those events cannot be regarded as false-negative outcomes [45]. Moreover, motor function compromise following supratentorial surgery may result from resection of associative motor areas such as the supplementary motor area (SMA) [45]. The classical described SMA syndrome is characterized by impaired ability to initiate voluntary movements (or speech) and resembles muscle weakness after injury of the CST [116]. The disturbances are, in most cases, temporary and may resolve within some weeks after surgery [116]. Given that the SMA is not assessed by MEP intraoperatively, SMA syndrome should not be considered false-negative [50,116,117]. These elucidations have been scarcely provided by authors, and this may imply that the real number of false-negative cases in the body of literature might be lower than previously thought.

### 4.8. MEPs as Surrogate Markers

Intraoperative MEPs have a dual function with regard to postoperative motor deficits. Firstly, they may serve as a diagnostic tool for the detection of neurological injury and prediction of postoperative motor status [28]. Secondly, they may contribute to the prevention of motor impairment, acting as surrogate endpoints that trigger a rescue intervention [28]. In the studies of our review, a reversible MEP change did not result in a postoperative motor deficit in most cases, and if a motor deficit manifested, it was transient, with only a few patients suffering a permanent deficit. Irreversible MEP changes were associated with a higher number of transient deficits compared with the reversible ones. Additionally, patients with irreversible alterations were more likely to develop permanent motor deficits. These observations suggest that in case of a successful intervention and reversal of MEP changes, the fatal damage is avoided, and if motor deficits occur, they are expected to resolve during the short-term follow-up. On the contrary, if the intervention was not effective and MEP changes were not reversed by the end of the surgery, postoperative motor deficits are more likely to occur and persist.

Table 3 provides an overview of intraoperative events and the rate of permanent motor deficits across different studies. Five percent of the studies had a high number of MEP changes related to all monitored patients, indicating that either these studies had a tendency towards (too) early alarms or alternatively high-risk surgeries were performed. Thirty-eight percent of the studies had a high rate of reversible related to all MEP changes, suggesting that an intraoperative intervention was successful in most cases. On the contrary, 14% of studies had a high rate of irreversible MEP changes indicating that the warning sign appeared rather (too) late or that it has been impossible by the surgeon to reverse the injury. Remarkably, no study in which the number of permanent motor deficits in the whole study population was high. Further, the bubble plot (Figure 4) illustrates that aneurysm procedures tend to cluster at the right-bottom part of the plot, signifying that vascular surgeries have a higher rate of reversible MEP changes compared with tumor surgery. This observation may be attributed to the fact that the rescue interventions in the two types of surgeries have a different potential for success. Admittedly, ischemia caused by a temporary clip can be reversed more easily than a mechanical injury of the CST.

The characterization of reversible MEP changes followed by postoperative motor deficit either as false-negative or as true-positive is a controversial point. In our DTA analysis, we regarded only irreversible MEP changes as positive results. A question that arises is whether the reversible alterations followed by motor deficit should be considered as true-positive cases that were partially reversed after successful intervention and the postoperative deficit as the residue of a partially reversed injury. This issue is more prominent in cases of immediate postoperative (early-transient) motor deficits that resolve in a short time. Indeed, the rescue intervention that takes place between the occurrence of the intraoperative alarm and the time of motor function evaluation may alter the outcome and confounds the assessment of MEPs as diagnostic tools. However, the diagnostic performance of MEPs in itself does not encompass the rescue intervention. Undoubtedly, the rescue intervention can be triggered by MEP alarms, but this is rather a matter of MEP performance as a surrogate and not as diagnostic markers. Thus, reversible MEP changes should be evaluated under the concept of MEP surrogacy [92].

MEPs may function as surrogate endpoints in the sense of substituting the postoperative motor deficit, which is the clinical endpoint but cannot be assessed on the anesthetized patient intraoperatively [28,92]. Although similar, surrogacy is not identical with diagnosis, and useful diagnostic markers are not necessarily useful surrogate markers and vice versa [118]. The diagnostic performance of MEPs is more related to the accuracy in prediction of the neurological deficit after surgery as a post-hoc event, whereas the surrogate performance is more related to the indication of the neurological injury during surgery as an intraoperative event that warrants intervention. The link to the postoperative condition lies in the effect of the triggered intervention on the onset of new deficits at that time [92]. Therefore, MEPs can be regarded as useful surrogate endpoints if successful intervention and reversal of MEP change are correlated with a lower number of postoperative motor deficits [28]. Holdefer et al. [28] reported this significant negative correlation between the proportion of reversible MEP alterations and the proportion of new motor deficits associated with MEP changes in vascular surgery (r_pearson_ = −0.81, *p* < 0.05). This negative association was also found during our own correlation analysis (r_spearman_ = −0.5, *p* < 0.001, Figure 4). Our results are in concordance with those by Holdefer et al. [28], but the difference is that in their study, a total of ten studies with intracranial aneurysm surgeries were included, while in our own correlation analysis, we included 59 studies (25 tumor surgery, 2 epilepsy surgery, 27 aneurysm/clipping, and 5 aneurysm/endovascular). The fact that this finding was replicated with a higher number of studies should corroborate this observation and suggest that reversible MEP changes following intervention indicate a successful reversal of an impending neurological injury and motor function preservation postoperatively.

### 4.9. Implications for Research

This scoping review summarized the existing evidence on MEP warning criteria in supratentorial surgery. The appraisal of this heterogeneous literature should provide some insights into research gaps and concepts. The sources of evidence on MEP warning criteria are observational studies, and it is unlikely that RCTs will be implemented in the future due to ethical considerations. Efforts for evidence synthesis are hindered by the heterogeneity of primary studies that lessens the power of summary estimates. In this regard, the interest should be focused on strategies to upgrade the evidence provided by observational studies and to mitigate the heterogeneity across them in order to enable evidence synthesis with more robust results in the future. First, basic concepts in the field of MEP warning criteria need to be clarified, and a standard terminology needs to be utilized by authors when reporting outcomes. To define the exact time-points at which a motor deficit is registered as early-transient, transient, or permanent, as well as which MEP alterations are considered reversible and irreversible, will facilitate a consensus among authors of future studies. The anesthetic regimen, stimulation protocols, minimal MEP amplitude, and recorded muscles should be described in detail. If more than one warning criteria or stimulation techniques are utilized, it is essential that the outcomes are clearly described separately so that the contribution of each criterion can be assessed. Data should be presented in such a way that the calculation of diagnostic accuracy measures is feasible. For this purpose, documentation protocols of intraoperative events with standard terminology might be established to facilitate intra-institutional as well as inter-institutional comparisons.

In this context, the sequence of actions undertaken when an alarm criterion occurs during surgery may be emphasized. Although the interventions after a warning sign were mentioned in most of the studies in our review, it was unclear how these actions were prioritized, if technical troubleshooting preceded the surgical measures and which intervention finally managed to reverse the alteration. These clarifications are essential to assess the efficacy of interventions and could contribute to the development of algorithms for the efficient management of intraoperative events signified by MEP alarms.

### 4.10. Limitations

We did not perform a risk of bias assessment of the included studies, as our objective was not to assess the quality of the existing studies but rather to provide a broad overview of the available evidence to identify gaps and clarify concepts. Secondly, in a few studies of the DTA analysis, MEP changes were not reported as irreversible but were characterized as significant. These MEP changes were regarded as irreversible in our analysis, although they were not clearly defined as such. Moreover, the discrimination between transient and permanent deficit was based on reporting provided by the authors and not on a specific time-point because of the heterogeneity in outcome reporting. Therefore, in a few studies, there were some deviations from the most commonly utilized cut-off time of 3 months. Further, we did not analyze studies, which performed subcortical mapping without simultaneous MEP monitoring as this was not within the scope of our review. Finally, it is important to highlight that correlation analysis should not be confounded by causality, especially when analyzing reversible MEP alterations.

Due to the severe heterogeneity of the included studies, we have not been able to pool the data, which would either allow a systematic review or even be the first step in preparing big data analysis. To train models in machine learning algorithms and thus, extract meaningful patterns or predict future classes relies on the way the data is collected. Further, it depends on the amount of data available. Small data has meaningful information but also contains a lot of noise. If we would train any machine learning model on such data as reported in our review, the chances that it will learn the noise “too well” might be huge, and when applied for deployment on new data, it will fail at making predictions due to “overfitting”. However, our scoping review may raise awareness to solve this limitation in future research studies.

## 5. Conclusions

In conclusion, the existing evidence for MEP warning criteria in supratentorial surgery derives from observational case series with high heterogeneity in terms of the study population, intraoperative neuromonitoring settings, utilized warning criteria, and outcome reporting. MEP signal loss was always considered as a major warning sign that triggered a cascade of actions in order to reverse impending motor damage. Additional common MEP warning criteria were amplitude reduction followed by threshold elevation. Irreversible MEP alterations were associated with a higher number of transient deficits compared with the reversible MEP changes and a higher likelihood that these motor deficits did persist.

In almost all studies, MEPs showed high specificity and NPV. Thus, the absence of an irreversible alteration may reassure the surgeon that the patient will not suffer a motor deficit in the short-term and long-term follow-up. On the contrary, less consistency was found for sensitivity estimates and PPV, which were rather low to modest, which could probably be attributed to the low prevalence of events. Further, in tumor surgery, the combination with subcortical mapping warning criteria did increase the test accuracy. Moreover, the role of neurophysiologist/neurosurgeon contextualization and intraoperative judgment are essential. MEPs seem to perform well as surrogate markers, and successful intervention followed by a reversal of MEP deterioration indicates postoperative motor function preservation.

In future studies, a consensus regarding the definitions of MEP alteration, critical duration of alterations, and outcome reporting should be established. Documentation protocols with standard terminology could facilitate comparisons and combinations of patient datasets to enable evidence synthesis.

## Figures and Tables

**Table 1:** Overview of the included studies with tumor and epilepsy surgery. Number of included patients, study design, stimulation parameters, MEP warning criteria, number of patients who met these criteria, as well as the relation with postoperative motor deficits are presented. *: The results are presented as the total number and percentage of patients with the indicated MEP warning criterion who developed a transient and permanent motor deficit. In studies with outcome reporting at distinct time-points, the results are presented as the total number and percentage of patients with the indicated MEP warning criterion who had a new postoperative motor deficit still present at the indicated time-point that resolved thereafter. #: Permanent motor deficit in 13/25 = not assessable. Transient motor deficit in 1/25 = not assessable. (The absence of cases with a deficit is indicated by the symbol “-”).

Authors	No. of Patients	Study Design/ Country	STT	Warning Criterion	MEP Changes	Postoperative Motor Deficit in Relation to MEP Change	
						Transient *	Permanent *
					**TUMORS AND OTHER BRAIN LESIONS**		
Giampiccolo et al. (2021) [29]	125	Retrospective Italy	DCS	AR > 50%	Upper Limbs AR/loss (*n* = 26)	2d: 3/26 (11.5%) 5 d: 6/26 (23%)	10/26 (38.5%)
					Lower Limbs AR/loss (*n* = 14)	2 d: 2/14 (14.3%) 5 d: 3/14 (21.4%)	3/14 (21.4%)
Gogos et al. (2020) [30]	58	Prospective USA	DCS TES ScS	AR > 20% MT ≤ 5 mA	IRR AR (*n* = 6)	2/6 (33.3%)	1/6 (16.6%)
					MT ≤ 5 mA (*n* = 18)	2/18 (11.1%)	1/18 (5.5%)
Mammadk-hanli et al. (2020) [31]	145	Retrospective Turkey	DCS	AR > 50% LTI > 10%	REV changes (*n* = 7)	4/7 (57.1%), not specified if transient or permanent	
					IRR changes (*n* = 14)	14/14 (100%), not specified if transient or permanent	
Seidel et al. (2020) [32]	182	Prospective Switzerland	DCS ScS	THI ≥ 4 mA MT ≤ 3 mA	MT ≤ 3 mA (*n* = 58)	24 h: 13/58 (22.4%) discharge: 14/58 (24.1%)	3/58 (5.1%)
					MT ≤ 3 mA+ IRR THI/loss (*n* = 3)	-	3/3 (100%)
Abboud et al. (2019) [33]	126	Prospective Germany	TES	AR > 50% THI > 20%i	REV AR (*n* = 2)	-	-
					IRR AR (*n* = 36)	24 h: 6/36 (16.7%) discharge: 6/36 (16.7%)	8/36 (22.2%)
					REV THI (*n* = 9)	-	-
					IRR THI (*n* = 25)	24 h: 7/25 (28.0%) discharge: 7/25 (28.0%)	8/25 (32.0%)
Majchrzak et al. (2018) [34]	35	Prospective Poland	TES	AR > 50%	REV AR (*n* = 7)	6/7 (85.7%)	1/7 (14.3%)
					IRR AR (*n* = 11)	1/11 (9.1%)	10/11 (90.9%)
Moiyadi et al. (2018) [35]	39	Prospective India	DCS TES ScS	TES:AR > 50–80% DCS:AR > 30–50% MT ≤ 10 mA	TESIRR AR (*n* = 1) IRR loss (*n* = 2)	- ½ (50.0%)	- -
					DCS REV AR (*n* = 1) REV loss (*n* = 1) IRR loss (*n* = 1)	- - 1/1 (100%)	- - -
					MT ≤ 10 mA (*n* = 13)	4/13 (30.8%)	-
					MT ≤ 10 mA + AR/loss (*n* = 3)	2/3 (66.6%)	-
Plans et al. (2017) [36]	92	Retrospective Spain	DCS ScS	AR > 50% THI ≥ 5 mA MT ≤ 5 mA	IRR THI (*n* = 12)	24 h: 4/12 (33.3%)	-
					IRR AR (*n* = 2)	-	2/2 (100%)
					IRR loss (*n* = 7)	-	6/7 (85.7%)
					MT ≤ 5 mA (*n* = 30)	24 h: 2/30 (6.7%)	9/30 (30.0%)
					MT ≤ 5 mA + AR (*n* = 7)	-	7/7 (100%)
					MT ≤ 5 mA + THI (*n* = 2)	24 h: 2/2 (100%)	-
					MT ≤ 5 mA + THI/AR (*n* = 9)	24 h: 2/9 (22.2%)	7/9 (77.8%)
Zhou et al. (2017) [37]	70	Retrospective China	DCS TES	AR > 80%	AR/loss (*n* = 6)	5/6 (83.3%)	1/6 (16.7%)
Abboud et al. (2016) [17]	93	Prospective Germany	TES	THI > 20%i	IRR THI (*n* = 13)	8/13 (61.5%)	5/13 (38.5%)
Boex et al. (2016) [38]	104	Retrospective Switzerland	DCS TES	AR > 50%	IRR AR/loss (*n* = 16)	1 d: 5/16 (31.3%) discharge: 3/16 (18.8%)	8/16 (50.0%)
Obermueller et al. (2015) [23]	105 gliomas	Retrospective Germany	DCS	AR > 50%	REV AR (*n* = 85)	14/85 (16.5%)	5/85 (5.9%)
					IRR AR (*n* = 11)	2/11 (18.2%)	8/11 (72.7%)
	53 metastases		DCS	AR > 50%	REV AR (*n* = 32)	5/32 (15.6%)	2/32 (6.3%)
					IRR AR/loss (*n* = 14)	-	3/14 (21.4%)
Shiban et al. (2015) [39]	14	Prospective Germany	DCS ScS	AR > 50% MT ≤ 3 mA	REV loss (*n* = 1)	1/1 (100%)	-
					IRR loss (*n* = 2)	1/2 (50.0%)	1/2 (50.0%)
Lee et al. (2014) [40]	84	Retrospective Korea	TES	AR > 50% LTI > 10%	IRR AR (*n* = 7)	-	3/7 (42.9%)
Gempt et al. (2013) [41]	70	Prospective Germany	DCS	AR > 50%	REV AR (*n* = 8)	2/8 (25.0%)	2/8 (25.0%)
					IRR AR (*n* = 13)	5/13 (38.5%)	8/13 (61.5%)
Ostrý et al. (2013) [42]	25	Prospective Czech Republic	DCS ScS	THI ≥ 2 mA MT ≤ 5 mA	THI (*n* = 6)	4/6 (66.6%)	-
					MT ≤ 5 mA (*n* = 10)	3/10 (30.0%)	1/10 (10%)
					MT ≤ 5 mA + MEP alteration (*n* = 2)	2/2 (100%)	-
Pastor et al. (2013) [43]	30	Prospective Spain	DCS TES	AR > 50% LTI > 10%	TES (*n* = 16)	1 w: 4/16 (25.0%)	3/16 (18.8%)
					DCS (*n* = 2)	-	-
Seidel et al. (2013) [8]	100	Prospective Switzerland	DCS ScS	THI ≥ 4 mA MT ≤ 3 mA	THI ≤ 15 min/unspecific changes (*n* = 18)	24 h: 5/18 (27.8%) Discharge: 2/18 (11.1%)	-
					THI ≥ 15 min (*n* = 8)	24 h: 2/8 (25.0%) Discharge: 3/8 (37.5%)	2/8 (25.0%)
					Loss ≥15 min (*n* = 4)	Discharge: 1/4 (25.0%)	3/4 (75.0%)
					MT ≤ 3 mA (*n* = 25)	24 h: 4/25 (16.0%) Discharge: 2/25 (8.0%)	2/25 (8.0%)
					MT ≤ 3 mA+ THI ≥ 15 min/Loss ≥15 min (*n* = 5)	24 h: 1/5 (20.0%) Discharge: 2/5 (40.0%)	2/5 (40.0%)
Sakurada et al. (2012) [44]	30	Retrospective Japan	DCS	AR > 50%	REV AR (*n* = 2)	1/2 (50.0%)	-
					IRR AR (*n* = 2)	-	2/2 (100%)
Senft et al. (2012) [45]	54	Retrospective Germany	TESDCS	AR > 50% THI ≥ 20 mA (TES) THI ≥ 3 mA (DCS)	MEP alterations (*n* = 7: 2 THI, 1 AR, 1 loss, 3 N/A)	4/7 (57.1%)	2/7 (28.6%)
Hatiboglu et al. (2010) [46]	16	Retrospective USA	DCS	MEP loss	Loss (*n* = 4)	1/4 (25.0%)	2/4 (50.0%)
Ichikawa et al. (2010) [47]	21	Retrospective Japan	DCS	AR > 50%	REV AR (*n* = 3)	1/3 (33.3%)	-
					REV loss (*n* = 1)	1/1 (100%)	-
					IRR loss (*n* = 1)	-	1/1 (100%)
Szelényi et al. (2010) # [48]	25	Prospective Germany	TES	AR > 50% THI > 20 mA or >100 V	REV AR (*n* = 3)	-	-
					IRR AR (*n* = 2)	2/2 (100%)	-
					REV loss (*n* = 6)	2/6 (33.3%)	2/6 (33.3%)
					IRR loss (*n* = 5)	2/5 (40.0%)	2/5 (40.0%)
					REV THI (*n* = 3)	-	-
					IRR THI (*n* = 8)	2/8 (25.0%)	3/8 (37.5%)
Kombos et al. (2009) [49]	15	Prospective Germany	TESScS	AR > 80% LTI > 30% MT ≤ 3 mA	REV AR + LTI (*n* = 5)	2/5 (40.0%)	-
Neuloh et al. (2009) [50]	191	Prospective Germany	DCS TES	AR > 50%	REV AR (*n* = 50)	19/50 (38.0%)	1/50 (2.0%)
					REV loss (*n* = 7)	2/7 (28.6%)	1/7 (14.3%)
					IRR AR (*n* = 26)	11/26 (42.3%)	5/26 (19.2%)
					IRR loss (*n* = 7)	1/7 (14.3%)	6/7 (85.7%)
Neuloh et al. (2007) [51]	88	Prospective Germany	DCS TES	AR > 50%	REV AR/loss (*n* = 26)	12/26 (46.2%)	-
					IRR AR (*n* = 8)	7/8 (87.5%)	1/8 (12.5%)
					IRR loss (*n* = 7)	-	7/7 (100%)
Suess et al. (2006) [15]	232	Unclear Germany	DCS	AR > 50% LTI > 5% PWI > 30%	REV changes(*n* = 27)	6/27 (22.2%)	-
					IRR changes (*n* = 20)	-	17/20 (85.0%)
Neuloh et al. (2004) [11]	159	Prospective Germany	DCS	AR > 50% LTI > 10%	REV AR (*n* = 16)	8/16 (50.0%)	1/16 (6.3%)
					IRR AR (*n* = 16)	7/16 (43.8%)	4/16 (25.0%)
					REV loss (*n* = 22)	8/22 (36.4%)	1/22 (4.5%)
					IRR loss (*n* = 10)	2/10 (20.0%)	8/10 (80.0%)
Kombos et al. (2001) [10]	70	Prospective Germany	DCS	AR > 80% LTI > 15%	REV LTI (*n* = 3)	-	-
					IRR LTI (*n* = 1)	1/1 (100%), not specified if transient orpermanent	
					REV loss (*n* = 7)	-	-
					IRR loss (*n* = 1)	-	1/1 (100%)
Zhou et al. (2001) [52]	50	Prospective USA	TES	AR > 50%	REV AR (*n* = 4)	-	
					IRR AR/loss (*n* = 8)	8/8 (100%), not specified if transient orpermanent	
Cedzich et al. (1996) [53]	25	Prospective Germany	DCS	MEP loss	REV loss (*n* = 9)	4/9 (44.4%)	1/9 (11.1%)
					IRR loss (*n* = 6)	-	3/6 (50.0%)
**EPILEPSY SURGERY**							
Koo et al. (2019) [54]	279	Prospective Korea	TES	AR > 50%	REV AR (*n* = 6)	1/6 (16.7%)	-
					REV loss (*n* = 4)	1/4 (25.0%)	-
Neuloh et al. (2010) [55]	86	Prospective Germany	DCSTES	AR > 50% LTI > 10–15%	REV changes (*n* = 20)	4/20 (20.0%)	-
					IRR changes (*n* = 11)	2/11 (18.2%)	5/11 (45.5%)

Abbreviations: AR: Amplitude Reduction; DCS: Direct Cortical Stimulation; i: ipsilateral; IRR: irreversible; LTI: Latency Increase; MT: Motor Threshold (=MEP threshold); N/A: Not available; PWI: Pulse Width Increase; REV: Reversible; ScS: Subcortical Simulation; STT: Stimulation Technique; TES: Transcranial Electrical Stimulation; THI: Stimulation Threshold Increase.

**Table 2:** Overview of included studies with aneurysm clipping and endovascular procedures for aneurysms. Number of included patients, study design, stimulation parameters, MEP warning criteria, number of patients who met these criteria as well as the relation with postoperative motor deficits are presented. Two case reports are also summarized in this table. *: The results are presented as the total number and percentage of patients with the indicated MEP warning criterion who developed a transient and permanent motor deficit. In studies with outcome reporting at distinct time-points, the results are presented as the total number and percentage of patients with the indicated MEP warning criterion who had a new postoperative motor deficit still present at the indicated time-point that resolved thereafter. £: 3/116 cases = not assessable. The absence of cases with a deficit is indicated by the symbol “-”.

Authors	No. of Patients	Study Design	STT	Warning Criterion	MEP Changes	Postoperative Motor Deficit in Relation to MEP Change	
						Transient *	Permanent *
**ANEURYSM CLIPPING**							
Guo et al. (2021) [56]	285	Retrospective China	TES	AR > 50%	REV AR/loss (*n* = 49)	5/49 (10.2%)	6/49 (12.2%)
					IRR AR/loss (*n* = 14)	1/14 (7.1%)	10/14 (71.4%)
Park et al. (2021) [57]	319	Retrospective Korea	TES	AR > 50%	REV AR (*n* = 1)	1/1 (100%)	-
					IRR AR (*n* = 3)	2/3 (66.6%)	1/3 (33.3%)
					IRR loss (*n* = 1)	-	1/1 (100%)
You et al. (2021) [58]	138	Retrospective China	TES	AR > 50%	REV AR (*n* = 28)	11/28 (39.3%), not specified if transient or permanent	
					IRR loss (*n* = 5)	3/5 (60.0%), not specified if transient or permanent	
Kameda et al. (2020) [59]	42	Retrospective Japan	DCS TES	AR > 50%	REV AR (*n* = 2)	1/2 (50.0%)	-
Byoun et al. (2019) [22]	115	Retrospective Korea	TES	AR > 50%	REV AR (*n* = 5)	-	2/5 (40.0%)
Greve et al. (2019) [60]	133	Retrospective Germany	TES	AR > 50%	REV AR (*n* = 8)	1/8 (12.5%)	1/8 (12.5%)
					IRR AR (*n* = 4)	-	-
					IRR AR (*n* = 1)	-	1/1 (100%)
Li et al. (2019) [61]	92	Retrospective China	TES	AR > 50%	REV AR/loss (*n* = 76)	24 h: 3/76 (3.9%) 1 w: 19/76 (25%)	15/76 (19.7%)
					IRR AR (*n* = 11)	1 w: 1/11 (9.1%)	9/11 (81.8%)
					IRR loss (*n* = 5)	1 w: 1/5 (20.0%)	4/5 (80.0%)
Choi et al. (2017) [12]	386	Retrospective Korea	TES	AR > 50% LTI > 10%	REV AR (*n* = 4)	-	-
					REV loss (*n* = 5)	1/5 (20.0%)	1/5 (20.0%)
					IRR loss (*n* = 1)	-	1/1(100%)
Komatsu et al. (2017) [62]	9	Retrospective Japan	DCS	AR > 50%	REV AR (*n* = 5)	-	-
Staarman et al. (2017) [63]	123	Retrospective USA	TES	AR > 50%	REV AR (*n* = 9 aneurysms)	1/9 (11.1%)	N/A
					IRR AR (*n* = 1 aneurysm)	1/1 (100%)	N/A
Kim et al. (2016) [64]	685	Retrospective Korea	TES	AR > 50%	REV AR (*n* = 30)	10/30 (33.3%), not specified if transient or permanent	
					IRR AR (*n* = 13)	6/13 (46.2%), not specified if transient or permanent	
Maruta et al. (2016) [65]	243	Retrospective Japan	DCS TES	AR > 50% (sMEP + mMEP)	REV AR (*n* = 47)	2/47 (4.2%)	1/47 (2.1%)
					IRR AR (*n* = 5)	-	-
					REV loss (*n* = 19)	3/19 (15.7%)	4/19 (21.0%)
					IRR loss (*n* = 2)	-	2/2 (100%)
Song et al. (2015) [66]	11	Unclear China	TES	AR > 50%	REV AR (*n* = 5)	2/5 (40.0%)	N/A
					IRR AR (*n* = 1)	1/1 (100%)	N/A
Sasaki et al. (2014) [67]	177	Prospective Japan	DCS TES	MEP loss	REV loss (*n* = 20)	1/20 (5.0%)	-
					IRR loss (*n* = 2)	-	2/2 (100%)
Takebayashi et al. (2014) [68]	50	Unclear Japan	DCS	MEP loss	REV loss (*n* = 15)	6/15 (40.0%)	-
					IRR loss (*n* = 4)	-	4/4 (100%)
Yue et al. (2014) [69]	43	Prospective China	TES	AR > 50%	REV AR (*n* = 5)	Immediate: 1/5 (20.0%)	-
					REV loss (*n* = 9)	-	1/9 (11.1%)
					IRR loss (*n* = 1)	-	1/1 (100%)
Dengler et al. (2013) [70]	30	Prospective Germany	TES	AR > 50% LTI > 10%	REV changes (*n* = 14 cases)	1/14 (7.1%)	-
					IRR changes (*n* = 1 case)	-	1/1 (100%)
Kang et al. (2013) [71]	37	Unclear China	TES	AR > 50%	AR (*n* = 8)	3/8 (37.5%)	N/A
Maruta et al. (2012) [72]	22	Prospective Japan	DCS TES	AR > 50%	REV AR (*n* = 1)	-	-
					REV loss (*n* = 3)	2/3 (66.6%)	-
Shi et al. (2012) [73]	68	Unclear China	TES	AR > 50%	REV AR (*n* = 6)	-	-
					IRR AR (*n* = 3)	-	3/3 (100%)
Motoyama et al. (2011) [74]	48	Retrospective Japan	DCSTES	AR > 50%	REV AR (*n* = 2)	-	N/A
					REV loss (*n* = 2)	-	N/A
					IRR loss (*n* = 1)	1/5 (20.0%), < 24 h	N/A
Irie et al. (2010) [75]	110	Retrospective Japan	TES	AR > 50% THI > 20 mA	REV AR (*n* = 2)	-	-
					REV +IRR loss (*n* = 4)	2/4 (50.0%)	-
Yeon et al. (2010) [76]	98	Prospective Japan	TES	AR > 50%	REV AR (*n* = 4)	-	-
					REV loss (*n* = 8)	1/8 (12.5%)	-
Szelényi et al. (2007) [77]	108	Prospective and Retrospective Germany/USA	DCS TES	AR > 50% THI > 20 mA(TES) THI > 2 mA(DCS)	TES changes (*n* = 9: 1 IRR THI, 1 REV AR, 6 REV loss, 1 IRR loss)	3/9 (33.3%)	-
					DCS changes (*n* = 13: 1 REV THI, 3 IRR THI, 2 REV AR, 6 REV loss, 1 IRR loss)	-	2/13 (15.4%)
Weinzierl et al. (2007) [78]	18	Prospective Germany	TES	AR > 50% LTI > 10%	REV AR (*n* = 8)	-	-
					IRR AR (*n* = 3)	-	-
Szelényi et al. (2006) £ [16]	116	Prospective and Retrospective Germany	DCS TES	AR > 50% THI > 20 mA	REV loss (*n* = 8)	1/8 (12.5%)	3/8 (37.5%)
					IRR loss (*n* = 2)	-	2/2 (100%)
					REV THI (*n* = 1)	1/1 (100%)	-
					IRR THI (n = 1)	-	1/1 (100%)
Horiuchi et al. (2005) [79]	53	Prospective Japan	DCS	AR > 50%	REV AR (*n* = 3)	-	-
					REV loss (*n* = 6)	3/6 (50.0%)	-
					IRR loss (*n* = 1)	-	1/1 (100%)
Suzuki et al. (2003) [19]	108	Prospective Japan	DCS	AR > 50%	REV changes (*n* = 19)	4/19 (21.0%): * < 24 h, REV loss	-
					IRR loss (*n* = 1)	-	1/1 (100%)
**ENDOVASCULAR PROCEDURES FOR ANEURYSMS**							
Nakagawa et al. (2020) [80]	164	Retrospective Japan	TES	AR > 50%	REV AR (*n* = 3)	-	N/A
					IRR AR (*n* = 2)	2/2 (100%)	N/A
					REV loss (*n* = 1)	-	N/A
					IRR loss (*n* = 1)	1/1 (100%)	N/A
Wilent et al. (2020) [81]	763	Retrospective USA	TES	AR >40%	IRR AR (*n* = 36)	12/36 (33.3%)	N/A
					REV AR (*n* = 15)	-	N/A
Lee et al. (2019) [82]	561	Retrospective Korea	TES	AR > 50% LTI > 10%	REV AR (*n* = 5)	-	N/A
					IRR AR (*n* = 1)	-	N/A
					IRR loss (*n* = 1)	1/1 (100%)	N/A
Piñeiro et al. (2015) [83]	8	Prospective Spain	TES	AR > 50%	REV AR (*n* = 1)	-	-
					IRR AR (*n* = 1)	-	1/1 (100%)
Hiraishi et al. (2011) [84]	7	Unclear Japan	TES	AR > 50%	REV AR (*n* = 3)	1/3 (33.3%)	N/A
**CASE REPORTS**							
Iwasaki et al. (2013) [85]	2 (1 awake)	Case report Japan	TES	AR > 50%	Absence of MEP AR > 50% for >10 min after clipping as an indicator for the preservation of the long insular artery		
Szelényi et al. (2003) [86]	1	Case report Germany	DCS TES	MEP loss	Arteriosclerotic aneurysm wall preventing the complete closure of the clip. REV loss and immediate postoperative hemiplegia still present at discharge (1 month).		

Abbreviations: AR: Amplitude Reduction; DCS: Direct Cortical Stimulation; IRR: irreversible; LTI: Latency Increase; mMEP: muscle motor-evoked potential; N/A: Not available; REV: Reversible; sMEP: spinal motor-evoked potential; STT: Stimulation Technique; TES: Transcranial Electrical Stimulation; THI: Stimulation Threshold Increase.

**Table 3:** Summary of events. The table summarizes the relative rates of all MEP changes as well as the rates of reversible and irreversible MEP changes and permanent postoperative motor deficits in each study.

Authors	Total No. of Patients (*n*)		No. of All MEP Changes (*n*)	No. of All New Motor Deficits (*n*)	No. of Permanent Motor Deficits (*n*)	No. of All MEP Changes/Total No of Patients (%)	No. of Reversible MEP Changes/No of All MEP Changes (%)	No. of Irreversible MEP Changes/No of All MEP Changes (%)	No. of Permanent Motor Deficits/Total No of Patients (%)
						**TUMORS AND OTHER BRAIN LESIONS**			
Giampiccolo et al. (2021) [29]	125	UL	26	63	22	21%	N/A	N/A	18%
	125	LL	14	21	13	11%	N/A	N/A	10%
Gogos et al. (2020) [30]	58		6	6	2	10%	0%	100%	3%
Mammadk-hanli et al. (2020) [31]	145		21	N/A	N/A	14%	33%	67%	N/A
Seidel et al. (2020) [32]	182		N/A	56	3	N/A	N/A	N/A	2%
* Abboud et al. (2019) [33]	126		72	44	18	14%	15%	85%	4%
Majchrzak et al. (2018) [34]	35		18	18	11	51%	39%	61%	31%
Moiyadi et al. (2018) [35]	39		6	7	0	15%	33%	67%	0%
** Plans et al. (2017) [36]	92		21	18	9	23%	0%	100%	11%
Zhou et al. (2017) [37]	70		6	10	1	9%	N/A	N/A	1%
Abboud et al. (2016) [17]	93		13	13	5	13%	0%	100%	5%
Boex et al. (2016) [38]	104		16	19	8	18%	0%	100%	8%
Obermueller et al. (2015) [23]	158		142	43	19	90%	82%	18%	12%
Shiban et al. (2015) [39]	14		3	5	1	21%	33%	67%	7%
Lee et al. (2014) [40]	84		7	14	6	8%	0%	100%	7%
Gempt et al. (2013) [41]	70		21	23	10	30%	38%	62%	14%
Ostrý et al. (2013) [42]	25		6	8	1	24%	N/A	N/A	4%
Pastor et al. (2013) [43]	30		12	8	3	40%	0%	100%	10%
Seidel et al. (2013) [8]	100		30	30	5	30%	60%	40%	5%
Sakurada et al. (2012) [44]	30		4	7	5	13%	50%	50%	17%
Senft et al. (2012) [45]	54		7	11	4	13%	N/A	N/A	7%
Hatiboglu et al. (2010) [46]	16		4	8	2	25%	N/A	N/A	13%
Ichikawa et al. (2010) [47]	21		5	3	1	24%	80%	20%	5%
* Szelényi et al. (2010) [48]	25		27	15	7	96%	44%	56%	25%
Kombos et al. (2009) [49]	15		5	2	0	33%	100%	0%	0%
Neuloh et al. (2009) [50]	191		90	52	15	47%	63%	37%	8%
Neuloh et al. (2007) [51]	88		41	27	8	47%	63%	37%	9%
Suess et al. (2006) [15]	232		47	35	19	20%	57%	43%	8%
Neuloh et al. (2004) [11]	159		64	61	14	40%	59%	41%	9%
Kombos et al. (2001) [10]	70		12	8	N/A	17%	83%	17%	N/A
Zhou et al. (2001) [52]	50		12	8	N/A	24%	33%	67%	N/A
Cedzich et al. (1996) [53]	25		15	9	5	60%	60%	40%	20%
						**EPILEPSY SURGERY**			
Koo et al. (2019) [54]	279		10	2	0	4%	100%	0%	0%
Neuloh et al. (2010) [55]	86		31	11	5	36%	65%	35%	6%
						**ANEURYSM CLIPPING**			
Guo et al. (2021) [56]	285		63	32	23	22%	78%	22%	8%
Park et al. (2021) [57]	319		12	10	6	4%	67%	33%	2%
You et al. (2021) [58]	138		33	17	N/A	24%	85%	15%	N/A
Kameda et al. (2020) [59]	42		2	2	0	5%	100%	0%	0%
Byoun et al. (2019) [22]	115		5	3	3	4%	100%	0%	3%
Greve et al. (2019) [60]	133		13	9	4	9%	61%	39%	3%
Li et al.(2019) [61]	92		92	52	28	100%	83%	17%	30%
Choi et al. (2017) [12]	386		10	8	6	3%	0%	100%	2%
Komatsu et al. (2017) [62]	9		5	0	0	56%	100%	0%	0%
Staarman et al. (2017) [63]	123		10	3	N/A	8%	90%	10%	N/A
Kim et al. (2016) [64]	685		43	36	N/A	6%	70%	30%	N/A
Maruta et al. (2016) [65]	243		73	18	11	30%	90%	10%	5%
Song et al. (2015) [66]	11		6	3	N/A	55%	83%	17%	N/A
Sasaki et al. (2014) [67]	177		22	6	2	12%	90%	10%	1%
Takebayashi et al. (2014) [68]	50		19	10	4	38%	79%	21%	8%
Yue et al. (2014) [69]	43		15	4	3	35%	93%	7%	7%
* Dengler et al. (2013) [70]	30		15	2	1	44%	93%	7%	3%
Kang et al. (2013) [71]	37		8	3	N/A	22%	0%	100%	N/A
Maruta et al. (2012) [72]	22		4	3	N/A	18%	100%	0%	N/A
Shi et al. (2012) [73]	68		9	N/A	N/A	13%	67%	33%	N/A
Motoyama et al. (2011) [74]	48		5	1	N/A	11%	80%	20%	N/A
Irie et al. (2010) [75]	110		6	8	N/A	5%	83%	17%	N/A
Yeon et al. (2010) [76]	98		12	1	0	12%	100%	0%	0%
Szelényi et al. (2007) [77]	108		22	5	2	13%	73%	27%	2%
Weinzierl et al. (2007) [78]	18		4	0	0	22%	25%	75%	0%
Szelényi et al. (2006) [16]	116		12	15	6	11%	75%	25%	5%
Horiuchi et al. (2005) [79]	53		10	4	1	19%	90%	10%	2%
Suzuki et al. (2003) [19]	108		20	5	1	19%	95%	5%	1%
						**ENDOVASCULAR PROCEDURES FOR ANEURYSMS**			
Nakagawa et al. (2020) [80]	164		7	10	N/A	4%	57%	43%	N/A
Wilent et al. (2020) [81]	763		51	13	N/A	7%	29%	71%	N/A
Lee et al. (2019) [82]	561		7	4	N/A	1%	71%	29%	N/A
Piñeiro et al. (2015) [83]	8		2	3	3	25%	50%	50%	38%
Hiraishi et al. (2011) [84]	7		3	1	N/A	43%	100%	0%	N/A

Color scale:   0–25%;   26–50%;   51–75%;   76–100%;   Not available/Not applicable. * In these studies, the calculations were done based on the number of total cases/events. ** Only 85 patients were available for analysis at 3 months.

## Supplementary Materials

The following are available online at https://www.mdpi.com/article/10.3390/cancers13112803/s1, Figure S1: Forest plot of sensitivity and specificity estimates for all motor deficits, Figure S2: Forest plot of sensitivity and specificity estimates for transient motor deficits, Figure S3: Forest plot of sensitivity and specificity estimates for early-transient motor deficits, Figure S4: Heatmap depicting the diagnostic accuracy measures of MEPs for permanent motor deficits, Figure S5: Heatmap depicting the diagnostic accuracy measures of MEPs for transient motor deficits, Figure S6: Heatmap depicting the diagnostic accuracy measures of MEPs for early-transient motor deficits, Figure S7: Heatmap depicting the diagnostic accuracy measures of MEPs for all motor deficits, Figure S8: Scatterplot with regression line and 95% confidence intervals depicting the negative correlation between the proportion of reversible MEP changes and the proportion of new motor deficits associated with MEP changes. Each dot represents one study. Spearman’s rank correlation coefficient = −0.498 (*p* < 0.001) Table S1: Definitions of true positive (TP), false positive (FP), false negative (FN), and true negative (TN) outcomes across the four different DTA sub-analyses, Table S2: Patient characteristics, neuromonitoring settings and interventions, Table S3: Diagnostic accuracy measures for all postoperative motor deficits, Table S4: Diagnostic accuracy measures for permanent postoperative motor deficits, Table S5: Diagnostic accuracy measures for transient postoperative motor deficits, Table S6: Diagnostic accuracy measures for early-transient postoperative motor deficits, Table S7: The table summarizes the relative rates of all motor deficits as well as the total number of early-transient, transient and permanent motor deficits in conjunction with MEP changes, Table S8: Causes of injury in tumor patients with permanent motor deficits.

## Appendix A

**Database and Search Strategy**
**PubMed** Key concepts Concept 1: Motor evoked potentials Keywords: “motor evoked potential*”[tw], MEP[tw] MeSH terms: “Evoked Potentials, Motor”[Mesh] Query 1: ((“motor evoked potential*”[tw]) OR (MEP[tw])) OR (“Evoked Potentials, Motor”[Mesh]) Concept 2: warning criteria Keywords: “warning criteri*”[tw], warning [tw], alarm [tw], alert [tw], “alarm criteri*”[tw], mapping[tw], monitoring[tw] MeSH terms: “Intraoperative Neurophysiological Monitoring”[Mesh], “Brain Mapping”[Mesh] Query 2: ((((((((“warning criteri*”[tw]) OR (warning [tw])) OR (alarm [tw])) OR (alert [tw])) OR (“alarm criteri*”[tw])) OR (mapping[tw])) OR (monitoring[tw])) OR (“Intraoperative Neurophysiological Monitoring”[Mesh])) OR (“Brain Mapping”[Mesh]) Concept 3: motor deficit Keywords: “motor deficit”[tw], paresis[tw], hemiparesis[tw], paralysis[tw] MeSH terms: “Predictive Value of Tests”[MeSH], “Paresis/prevention and control”[Mesh], “Paralysis/prevention and control”[Mesh], “Neurologic Manifestations/injuries”[Mesh], “Neurologic Manifestations/prevention and control”[Mesh], “Neurologic Manifestations/surgery”[Mesh] Query 3: ((((“Predictive Value of Tests”[Mesh]) AND “Paresis/prevention and control”[Mesh]) AND “Paralysis/prevention and control”[Mesh]) AND (“Neurologic Manifestations/injuries”[Mesh] OR “Neurologic Manifestations/prevention and control”[Mesh] OR “Neurologic Manifestations/surgery”[Mesh])) OR (“motor deficit”[tw])) OR (paresis[tw])) OR (hemiparesis[tw])) OR (paralysis[tw]) Concept 4: supratentorial brain surgery Keywords: supratentorial [tw], brain surgery[tw], “supratentorial surgery”[tw], tumor*[tw], aneurysm*[tw], epilepsy[tw] MeSH terms: “Neurosurgical Procedures”[Mesh], “Brain Injuries/diagnosis”[Mesh], “Intracranial Aneurysm/surgery”[Mesh], “Brain Neoplasms/surgery”[Mesh], “Epilepsy/surgery”[Mesh], “Central Nervous System Vascular Malformations/surgery”[Mesh] Query 3: ((((((((((((supratentorial[tw]) OR (brain surgery[tw])) OR (“supratentorial surgery”[tw])) OR (tumor*[tw])) OR (aneurysm*[tw])) OR (epilepsy[tw])) OR (“Neurosurgical Procedures”[Mesh])) OR (“Brain Injuries/diagnosis”[Mesh])) OR (“Intracranial Aneurysm/surgery”[Mesh])) OR (“Brain Neoplasms/surgery”[Mesh]))) OR (“Epilepsy/surgery”[Mesh])) OR (“Central Nervous System Vascular Malformations/surgery”[Mesh]) **Combined query** (((((((((((“warning criteri*”[tw]) OR (warning [tw])) OR (alarm [tw])) OR (alert [tw])) OR (“alarm criteri*”[tw])) OR (mapping[tw])) OR (monitoring[tw])) OR (“Intraoperative Neurophysiological Monitoring”[Mesh])) OR (“Brain Mapping”[Mesh])) AND (((((“Predictive Value of Tests”[Mesh]) AND “Paresis/prevention and control”[Mesh]) AND “Paralysis/prevention and control”[Mesh]) AND (“Neurologic Manifestations/injuries”[Mesh] OR “Neurologic Manifestations/prevention and control”[Mesh] OR “Neurologic Manifestations/surgery”[Mesh])) OR (“motor deficit”[tw])) OR (paresis[tw])) OR (hemiparesis[tw])) OR (paralysis[tw])) AND (((((((((((((supratentorial[tw]) OR (brain surgery[tw])) OR (“supratentorial surgery”[tw])) OR (tumor*[tw])) OR (aneurysm*[tw])) OR (epilepsy[tw])) OR (“Neurosurgical Procedures”[Mesh])) OR (“Brain Injuries/diagnosis”[Mesh])) OR (“Intracranial Aneurysm/surgery”[Mesh])) OR (“Brain Neoplasms/surgery”[Mesh]))) OR (“Epilepsy/surgery”[Mesh])) OR (“Central Nervous System Vascular Malformations/surgery”[Mesh]))) AND (((“motor evoked potential*”[tw]) OR (MEP[tw])) OR (“Evoked Potentials, Motor”[Mesh]))
**Embase, Scopus, CINAHL, Cochrane Library** (“warning criteri*” OR warning OR alarm OR alert OR “alarm criteri*” OR mapping OR monitoring OR “Intraoperative Neurophysiological Monitoring” OR “Brain Mapping”) AND (“motor deficit” OR paresis OR hemiparesis OR paralysis) AND (supratentorial OR brain surgery OR “supratentorial surgery” OR tumor* OR aneurysm* OR epilepsy OR arteriovenous malformation) AND (“motor evoked potential*” OR MEP)
**Grey literature databases (OpenGrey, NTIS, British Library Direct Plus, York’s CRD, Mednar)** (“warning criteri*” OR warning OR alarm OR alert OR “alarm criteri*” OR mapping OR monitoring OR “Intraoperative Neurophysiological Monitoring” OR “Brain Mapping”) AND (“motor deficit” OR paresis OR hemiparesis OR paralysis) AND (supratentorial OR brain surgery OR “supratentorial surgery” OR tumor* OR aneurysm* OR epilepsy OR arteriovenous malformation) AND (“motor evoked potential*” OR MEP)

## Appendix B

**Authors**	**Reason for Exclusion**
After full-text review (*n* = 140)	
Bidkar et al. (2021) [119]	Not a primary study
Keeble et al. (2021) [120]	No preoperatively defined MEP warning criteria
Lee et al. (2021) [121]	No MEP warning criteria
Simon et al. (2021) [122]	No MEP warning criteria
Wang et al. (2021) [123]	No motor outcome analysis
Bander et al. (2020) [124]	No MEP warning criteria
Brage et al. (2020) [125]	No sufficient motor outcome data in conjunction with MEP warning criteria
Fekete et al. (2020) [126]	Only spinal and brainstem lesions
Hayashi et al. (2020) [127]	No MEP warning criteria
Jahodová et al. (2020) [128]	No preoperatively defined cutoff value for MEP warning criteria
Kim et al. (2020) [129]	No MEP warning criteria
Lee et al. (2020) [130]	No MEP warning criteria
Porčnik et al. (2020) [131]	Motor outcome data of asleep patients cannot be distinguished from those of awake patients
Roth et al. (2020) [132]	No cutoff values for MEP warning criteria
Balaji et al. (2019) [133]	No MEP warning criteria
Chung et al. (2019) [134]	Only analysis of false-positive and false-negative cases
Hu et al. (2019) [135]	No MEP warning criteria
Kanaya et al. (2019) [136]	No MEP warning criteria
Rossi et al. (2019) [137]	No sufficient motor outcome data in conjunction with MEP warning criteria
Wang et al. (2019) [138]	No MEP warning criteria
Della Puppa et al. (2018) [139]	No MEP warning criteria
Han et al. (2018) [140]	No MEP warning criteria
Silverstein et al. (2018) [141]	No MEP warning criteria
Skrap et al. (2018) [142]	No clear MEP monitoring warning criteria and no sufficient motor outcome data in conjunction with MEPs-mixture with SSEP
Umemura et al. (2018) [143]	Motor outcome data of patients with supratentorial lesions were not clearly reported and could not be extracted
Wakui et al. (2018) [144]	No MEP warning criteria
Abboud et al. (2017) [100]	Predefined analysis of patients without a postoperative deficit and without MEP warning criteria to investigate pneumocephalus with MRI
Akiyama et al. (2017) [145]	No preoperatively defined MEP warning criteria
Lv X et al. (2017) [146]	No MEP monitoring
Pintea et al. (2017) [147]	No MEP warning criteria
Takagaki et al. (2017) [148]	No MEP monitoring
Carrabba et al. (2016) [149]	No MEP warning criteria
Gripp et al. (2016) [150]	No MEP warning criteria
Grossauer et al. (2016) [151]	No preoperatively defined MEP warning criteria
Ikedo et al. (2016) [152]	Evacuation of hematoma and control of the presence of MEPs after evacuation -not tumor, vascular or epileptogenic lesion
Imai et al. (2016) [153]	No motor outcome analysis
Isozaki et al. (2016) [154]	No MEP warning criteria
Koenig et al. (2016) [155]	No preoperatively defined MEP warning criteria
Nakagomi et al. (2016) [156]	No MEP warning criteria
Rossetto et al. (2016) [157]	No MEP warning criteria
Zhuang et al. (2016) [158]	Data for patients with supratentorial lesions cannot be extracted with certainty
Zhukov et al. (2016) [159]	No preoperatively defined MEP warning criteria
Wang et al. (2016) [160]	No MEP warning criteria
Eldin et al. (2015) [161]	No MEP warning criteria
Erdoğan et al. (2015) [162]	“Presence or absence” warning criterion but only the spinal and brainstem cases are adequately related to postoperative motor outcome
Jo et al. (2015) [163]	No MEP warning criteria
Joksimovic et al. (2015) [164]	No MEP warning criteria
Okamoto et al. (2015) [165]	No MEP warning criteria
Quan et al. (2015) [166]	No MEP warning criteria
Rashad et al. (2015) [167]	No MEP warning criteria
Shiban et al. (2015) [102]	No clear MEP warning criteria
Udaka et al. (2015) [168]	No MEP warning criteria
Raabe et al. (2014) [105]	Overlapping series from the same institution
Sahaya et al. (2014) [169]	Only 3 cases with MEP monitoring and no reporting of motor outcome for them
Schucht et al. (2014) [170]	No sufficient motor outcome data in conjunction with MEP warning criteria and mapping thresholds
Bulusu et al. (2013) [171]	No MEP warning criteria
Krieg et al. (2013) [172]	Overlapping series from the same institution
Krieg et al. (2013) [173]	No sufficient motor outcome data in conjunction with MEP warning criteria, data for asleep patients, and postoperative motor deficit cannot be extracted with certainty
Shah P.A. (2013) [174]	No clear MEP warning criteria
Vassal et al. (2013) [175]	No MEP warning criteria
Chen et al. (2012) [176]	No clear MEP warning criteria
Horton et al. (2012) [177]	No cutoff values for MEP warning criteria
Krieg et al. (2012) [96]	Overlapping series from the same institution
Ohue et al. (2012) [107]	No preoperatively defined MEP warning criteria
Ritzl EK. (2012) [178]	Not a primary study
Schucht et al. (2012) [179]	No sufficient motor outcome data in conjunction with MEP warning criteria
Seidel et al. (2012) [97]	No MEP monitoring warning criteria, only mapping warning criteria
Uchino et al. (2012) [180]	No sufficient motor outcome data in conjunction with MEP warning criteria
Zhu et al. (2012) [181]	No clear MEP warning criteria
Chang et al. (2011) [182]	No MEP warning criteria
Chen et al. (2011) [183]	No MEP monitoring
Fukaya et al. (2011) [184]	No MEP warning criteria
González-Darder(2011) [185]	No MEP warning criteria
Li et al. (2011) [186]	No sufficient motor outcome data in conjunction with MEP warning criteria
Lin et al. (2011) [187]	No cutoff values for MEP warning criteria
Nossek et al. (2011) [108]	No clear MEP monitoring warning criteria. No sufficient motor outcome data in conjunction with mapping warning criteria for the asleep patients.
Prabhu et al. (2011) [106]	No preoperatively defined MEP warning criteria
Szelényi et al. (2011) [103]	No MEP warning criteria
Tanaka et al. (2011) [188]	Numbers of MEP changes reported in percentages. Only motor palsy <2/5 MMRC and not postoperative motor deterioration is reported.
von Der Brelie et al. (2011) [189]	No MEP monitoring
Walter et al. (2011) [190]	No MEP warning criteria
Bello et al. (2010) [191]	No preoperatively defined MEP warning criteria
Bozzao et al. (2010) [192]	No MEP warning criteria
Feigl et al. (2010) [193]	No sufficient motor outcome data in conjunction with MEP warning criteria
Juretschke et al. (2010) [194]	No MEP warning criteria
Maesawa et al. (2010) [195]	No preoperatively defined MEP warning criteria
Sala et al. (2010) [196]	Not a primary study
Sanai et al. (2010) [197]	Not a primary study
Talacchi et al. (2010) [1]	No MEP monitoring warning criteria
Tanaka et al. (2010) [198]	Earlier series from the same institution
Yang et al. (2010) [199]	No MEP warning criteria
Gorji et al. (2009) [200]	No clear MEP warning criteria
Hattingen et al. (2009) [201]	Data for patients with supratentorial lesions cannot be extracted with certainty
Kamada et al. (2009) [109]	No preoperatively defined MEP warning criteria
Kombos et al. (2009) [202]	No sufficient motor outcome data in conjunction with MEP warning criteria
Krammer et al. (2009) [203]	Motor outcome data of patients with supratentorial lesions were not clearly reported and could not be extracted
Ozawa et al. (2009) [204]	No MEP warning criteria
Simon et al. (2009) [205]	No MEP warning criteria
Sugita et al. (2009) [206]	No MEP warning criteria
Von Lehe et al. (2009) [207]	No MEP warning criteria
Yamaguchi et al. (2009) [208]	No motor outcome analysis
Calancie et al. (2008) [209]	Only spinal cases
Berman et al. (2007) [210]	No MEP warning criteria
Mikuni et al. (2007) [211]	No MEP warning criteria
Neuloh et al. (2007) [212]	Overlapping series from the same institution
Szelényi et al. (2007) [6]	No MEP warning criteria
Yamaguchi et al. (2007) [213]	No motor outcome analysis
Fujiki et al. (2006) [214]	No clear preoperatively defined MEP warning criteria
Okada et al. (2006) [215]	No MEP warning criteria
Kamada et al. (2005) [216]	No MEP warning criteria
Szelényi et al. (2005) [101]	No motor outcome data in conjunction with MEP warning criteria
Keles et al. (2004) [217]	No MEP warning criteria
Kombos et al. (2004) [218]	No MEP warning criteria
Neuloh et al. (2004) [219]	No clear and preoperatively defined MEP warning criteria
Quiñones-Hinojosa et al. (2004) [220]	No sufficient motor outcome data in conjunction with MEP warning criteria
Sakuma et al. (2004) [221]	No MEP warning criteria
Signorelli et al. (2004) [222]	No MEP warning criteria
Yamamoto et al. (2004) [223]	Only D-wave recording
Duffau et al. (2003) [224]	No MEP monitoring warning criteria
Fukaya et al. (2003) [225]	Inclusion criterion in the study that the patient did not exhibit MEP amplitude decrease of >50% (warning criteria) intraoperatively
Sala et al. (2003) [111]	No sufficient motor outcome data in conjunction with MEP warning criteria
Suess et al. (2002) [226]	No MEP warning criteria
Kombos et al. (2000) [227]	No MEP warning criteria
Kofler et al. (1999) [228]	No MEP warning criteria
Kombos et al. (1999) [229]	No MEP warning criteria
Rohde et al. (1999) [230]	MEPs elicited through Transcranial Magnetic Stimulation(TMS)
Yingling et al. (1999) [231]	No MEP warning criteria
Krombach et al. (1998) [232]	No MEP warning criteria
Zentner et al. (1998) [233]	No correlation of MEPs with postoperative but with preoperative motor deficit
Zentner et al. (1996) [117]	No MEP warning criteria
Kawaguchi et al. (1996) [234]	No clear MEP warning criteria
Maertens et al. (1996) [235]	No clear MEP warning criteria
Pechstein et al. (1996) [236]	No MEP warning criteria
Rodi et al. (1996) [237]	No MEP warning criteria
Skirboll et al. (1996) [238]	No MEP warning criteria
Taniguchi et al. (1993) [5]	No MEP warning criteria
Ebeling et al. (1992) [239]	No MEP warning criteria
Schramm et al. (1990) [240]	Only SSEP monitoring
Zentner et al. (1988) [241]	No clear MEP warning criteria
After abstract screening (n = 73)	
Chen et al. (2021) [242]	Τechnical report, presentation of a new technique
Machetanz et al. (2021) [243]	MEPs elicited through Transcranial Magnetic Stimulation(TMS)
Cattaneo et al. (2020) [244]	Use of MEPs to investigate brain connectivity
Kang et al. (2020) [245]	Not a primary study
Policicchio et al. (2020) [246]	Not a primary study
Shibata et al. (2020) [247]	Awake craniotomy
Wang et al. (2020) [248]	Only SSEP analysis
Zuo et al. (2020) [249]	Not a primary study
NCT04178395(2019) [250]	Protocol for clinical trial
Hiruta et al. (2019) [251]	Τechnical report (cortical and subcortical stimulation ratio), no motor outcome data
Zhu et al. (2019) [91]	Not a primary study
Rajan et al. (2018) [252]	Not a primary study
Valci et al. (2018) [253]	No MEP monitoring to avoid a postoperative deficit, no MEP warning criteria
Abdulrauf et al. (2017) [254]	Awake surgery
Benavides et al. (2017) [255]	Not a clinical study; study in pigs
Bharadwaj et al. (2017) [256]	Τechnical report, application, and feasibility of a new monitoring system
Calancie B. (2017) [257]	Not a primary study
Grasso et al. (2017) [258]	Not a primary study
Hemmer et al. (2017) [259]	Not a primary study
Journée et al. (2017) [20]	Not a primary study
Ku et al. (2017) [260]	Case report of a patient with vestibular schwannoma
Liu et al. (2017) [21]	Spinal surgery
MacDonald DB. (2017) [9]	Not a primary study
Moser et al. (2017) [261]	MEPs elicited through Transcranial Magnetic Stimulation (TMS)
Sanmillan et al. (2017) [98]	Not a primary study
Schucht et al. (2017) [104]	Not a primary study
Thomas et al. (2017) [89]	Not a primary study
Alimohamadi et al. (2016) [262]	Awake craniotomy
Coppola et al. (2016) [263]	Not a primary study
Holdefer et al. (2016) [28]	Not a primary study
König, R. (2016) [264]	Not a primary study
Raabe et al. (2016) [265]	Not a primary study
Yao et al. (2016) [266]	The term MEP referred to Meningiomas-en-plaque, no MEP monitoring
Holdefer et al. (2015) [92]	Not a primary study
Ottenhausen et al. (2015) [267]	Not a primary study
Sala et al. (2015) [87]	Not a primary study
Nakamura et al. (2014) [268]	Only abstract available
Suzuki et al. (2014) [269]	Awake aneurysm clipping
Yang et al. (2014) [270]	Intraoperative neuromonitoring used as a mapping technique to find the corticospinal projections
Landazuri et al. (2013) [271]	Not a primary study
MacDonald et al. (2013) [7]	Not a primary study
Rajapakse et al. (2013) [272]	Not a primary study
Yamashita et al. (2013) [273]	Only abstract available
Bacigaluppi et al. (2012) [274]	Not a primary study
De Witt Hamer et al. (2012) [275]	Not a primary study
Emerson et al. (2012) [276]	Not a primary study
Hotson et al. (2012) [277]	Electrocorticography analysis
Ito et al. (2012) [278]	Spinal surgery
Guo et al. (2011) [90]	Review/Not a primary study
Guo et al. (2011) [279]	Letter to the Editor/not a primary study
Li et al. (2011) [280]	Case report of a patient with high-grade brainstem glioma
Deiner S. (2010) [281]	Spinal surgery
Pabon et al. (2010) [282]	Only abstract available
Lefaucheur et al. (2009) [283]	Electrode placement for neuropathic pain treatment
Sun et al. (2009) [284]	MEPs elicited through Transcranial Magnetic Stimulation (TMS)
Duffau, H. (2008)-1 [285]	Not a primary study
Duffau, H. (2008)-2 [286]	Not a primary study
Takashima et al. (2008) [287]	Not a primary study
Duffau H. (2007) [288]	Not a primary study
Tharin et al. (2007) [289]	Not a primary study
Duffau, H. (2006) [290]	Not a primary study
Schramm et al. (2006) [291]	Only abstract available
Shinoura et al. (2006) [292]	Awake surgery
Kuzniecky et al. (2005) [293]	Not a primary study
Binder et al. (2004) [294]	Correlation of intraoperative neuromonitoring and imaging with Kernohan’s notch syndrome
Hashiguchi et al. (2004) [295]	Only abstract available
Kondo et al. (2004) [296]	Only abstract available
Neuloh et al. (2004) [297]	Not a primary study
Sala et al. (2002) [93]	Not a primary study
Di Lazzaro et al. (1999) [298]	Neurological and not neurosurgical patients
Calancie et al. (1998) [299]	Spinal lesions
Reinhardt et al. (1996) [300]	Τechnical report, presentation of an optical navigation system
Newlon et al. (1984) [301]	MEPs in diagnosis/prognosis/follow-up. Not intraoperatively.
**After title screening: Duplicate or irrelevant records (*n* = 381)**	
**** Additional clarifications** - Not a primary study: review/meta-analysis/letter to the editor/technical report. - No MEP warning criteria: MEP monitoring may have been applied, but no MEP warning criteria were defined. - No clear MEP warning criteria: The authors reported that intraoperative MEP changes were considered, but it was not specified which criteria (amplitude, threshold, latency, and other) or which cut-off values were used. - No sufficient motor outcome data in conjunction with MEP warning criteria: The occurrence of MEP warning criteria and/or motor deficits were reported for the overall study population without specifying how many patients with intraoperative MEP alterations had postoperative motor deficits or vice versa, which was the outcome of interest for the scoping review. - No preoperatively defined MEP warning criteria: MEP warning criteria had to be determined a priori, namely before the operation. Postoperative reviews of monitoring datasets with post-hoc defined cut-off values as warning criteria, and analysis of motor outcomes were not eligible. A preoperatively determined warning criterion could be used to trigger intervention that may have altered the final results of the study. In the case of a postoperatively defined warning criterion, the intraoperative actions were not driven by alarm criteria, and the final results should not be regarded as comparable with those of studies with a priori defined alarm criteria. - No preoperatively defined cutoff value for MEP warning criteria: The cut-off values for MEP warning criteria (e.g., amplitude reduction >50%, threshold elevation >20 mA, latency increase >10%, etc.) had to be preoperatively defined. - No motor outcome analysis: Postoperative motor outcome was not reported. The study reported postoperative radiologic findings or sensory/cognitive/other deficits but no motor deficits. - Motor outcome data of anesthetized patients with supratentorial lesions were not clearly reported and could not be extracted: In studies with mixed populations (namely, anesthetized patients with supratentorial lesions and awake patients or infratentorial/spinal lesions). The data for supratentorial/anesthetized patients were not separately tabulated or reported in a way that they could be clearly distinguished from those with infratentorial/spinal/awake patients and extracted. - No MEP monitoring warning criteria, only mapping warning criteria: Our aim was to analyze MEP alarm criteria during continuous muscle MEP monitoring. Mapping criteria as complementary criteria were taken into consideration but only in studies that combined mapping with MEP monitoring. Studies with Penfield mapping without MEP monitoring were not included in the scoping review.	
