# Supplementary material for: Motor Evoked Potential Warning Criteria in Supratentorial Surgery: A Scoping Review
Source: Cancers (Basel). 2021 Jun 4;13(11):2803. doi: 10.3390/cancers13112803 (PMC8200078; doi:10.3390/cancers13112803)
Supplement: Supplementary file 1 [file cancers-13-02803-s001.zip › cancers-1223250-supplementary.pdf]

Supplementary Material

Supplementary Table S1: Definitions of true positive (TP), false positive (FP), false negative (FN), and true negative (TN) outcomes across the four different DTA sub-analyses.

	**True Positive(TP)**	**False Positive(FP)**	**False Negative(FN)**	**True Negative(TN)**
**Early-Transient Motor Deficit Analysis**	Irreversible MEP change and early-transient motor deficit	Irreversible MEP change without early-transient motor deficit	Reversible MEP change/no MEP change and early-transient motor deficit	Reversible MEP change/no MEP change without early-transient motor deficit
**Transient Motor Deficit Analysis**	Irreversible MEP change and transient motor deficit	Irreversible MEP change without transient motor deficit	Reversible MEP change/no MEP change and transient motor deficit	Reversible MEP change/no MEP change without transient motor deficit
**Permanent Motor Deficit Analysis**	Irreversible MEP change and permanent motor deficit	Irreversible MEP change without permanent motor deficit	Reversible MEP change/no MEP change and permanent motor deficit	Reversible MEP change/no MEP change without permanent motor deficit
**All Motor Deficits Analysis**	Irreversible MEP change and motor deficit	Irreversible MEP change without any motor deficit	Reversible MEP change/no MEP change and motor deficit	Reversible MEP change/no MEP change without any motor deficit

Supplementary Table S2: Patient characteristics, neuromonitoring settings and interventions

**Authors**	**Type of lesion (Age of patients)**	**Stimulation Parameters**	**Recorded Muscles**	**Intervention after warning**
**TUMORS AND OTHER BRAIN LESIONS**				
Giampiccolo et al.(2021)	glioma (low/high grade)<br>cavernoma<br>ependymoma<br>metastasis<br>meningioma<br>lymphoma<br>DNET<br><br>(mean age =49y, SD =15)	-DCS<br>- Multi-train<br>(p=5, ISI=2ms, pd=0.5 ms)	APB,ED, QUAD,AH,TA	N/A
Gogos et al.(2020)	glioma<br>ependymoma<br><br>(17-81y)	-DCS & TES<br>-m/bDsCS<br>-Multi-train<br>(p=0-9, pd=0.05-0.5 ms, ISI= 1-4 ms, si/TES=0–200 V,si/DCS=0-20mA,si/DsCS≤20mA)	Contralateral:<br>face, upper arm, forearm, hand, upper leg, lower leg, and foot muscles	Halt of resection
Mammadk-hanli et al. (2020)	glioma<br>(low/high grade)<br>metastasis<br>cavernoma<br>meningioma<br>AVM	-DCS<br>-Multi-train<br>(p=5, f=500 Hz, pd=0.3 ms, si=180V)	N/A	-Halt of resection and waiting for 10 minutes<br>-60 Hz interference control<br>-Steroid
Seidel et al.(2020)	glioma<br>metastasis<br>vascular lesions<br><br>(mean age =48y, SD =15)	-DCS<br>- mDsCS<br>- Multi-train<br>(p=5, pd=0.5 ms ,f=250 Hz, ISI=4 ms,si/DsCS≤20mA)	Contralateral:<br>face, distal and proximal upper and lower limbs	-Halt of resection(stop of resection if ≥15 min MEP change)<br>-Ruling out technical confounders<br>-Normalizing anesthesia<br>-Increase of CPP<br>-Nimodipine<br>-Removal of brain retractor
Abboud et al.(2019)	unilateral supratentorial lesions not involving the PMC<br><br>(mean age =52y, SD =16)	-TES<br>- Multi-train<br>(p=5, ISI=2ms, pd=0.5 ms, si≤400V)	Bilateral:<br>APB,TA,OO	-Halt of resection(stop of resection if ≥15 min MEP change)<br>-Warm irrigation<br>-Reposition of brain retractor

Majchrzak et al.(2018)	thalamic tumors<br><br>(20–65y,mean age=37y)	-TES<br>- Multi-train<br>(p=4-6, ISI=3–4 ms, pd=0.5 ms, si≤220 mA)	-Contralateral: thenar,hypothenar, triceps surae,TA<br><br>-Ipsilateral thenar (negative control)	N/A
Moiyadi et al.(2018)	glioma<br>(low/high grade)<br>metastasis<br>ependymoma<br>embryonal tumors<br><br>(3-70y, mean age =38.3y)	-DCS &TES<br>-mDsCS<br>-TES(Multi-train, p= 5, ISI=3ms, pd=0.5ms)<br>-DCS(Multi-train, p= 4, ISI=4ms, pd=0.5ms)	-Contralateral: FCR,APB,TA,EDB<br><br>-One ipsilateral muscle (negative control)	-Halt of resection<br>-Ruling out hemodynamic, systemic and anesthetic factors and strip electrode displacement<br>-Release of brain retraction<br>-Warm irrigation(5-10 min)
Plans et al.(2017)	glioma<br><br>(17–73 y, mean age =47.29 y)	-DCS<br>-mDsCS<br>- Multi-train (p=5, ISI=4ms, pd=0.5ms, si/DCS≤25mA, si/DsCS≤25mA)	Bilateral: APB,ED,AH,TA	-Halt of resection<br>-Ruling out strip electrode displacement<br>-Increase of BP<br>-Papaverine<br>-Warm irrigation
Zhou et al.(2017)	AVM<br><br>(6–76y,mean age= 34.9 y)	-DCS &TES<br>- Multi-train<br>(p=3-5, ISI=1-5ms)	N/A	-Increase of BP<br>-Cooling<br>-Inducing burst suppression<br>-Removal of the clip or retractor
Abboud et al.(2016)	gliomas not involving the PMC<br><br>(25–81 y,mean age=56 y)	-TES<br>- Multi-train<br>(p=5, ISI=2ms, pd=0.5 ms,si≤400V)	Bilateral: APB,TA,OO	-Halt of resection<br>-Reposition of brain retractors<br>-Warm irrigation
Boex et al.(2016)	glioma<br>meningioma<br>metastasis<br>dysplasia<br><br>(1-79y,median age =53y)	-DCS &TES<br>- Multi-train<br>(p=5, f=350 Hz, ISI=2.9 ms, pd= 0.4ms, si/DCS≤ 12 mA, si/TES≤180 mA)	-Contralateral: BB,FCR,thenar,lumbrical QUAD,TA,GAST, AH<br><br>-Ipsilateral hand and foot muscle (negative control)	-Halt of resection<br>-Verification of MEP decrease with a stimulation increase(max 5%)<br>-Imaging and neuronavigation reappraisal
Obermueller et al.(2015)	glioma<br>metastasis	-DCS<br>- Multi-train<br>(p=5, f=350 Hz, pd=0.2-0.3 ms, si=6-30mA)	-Contralateral: BB,FCR,APB,ADM,TA	-Halt of resection<br>-Removal of spatulas<br>-Irrigation(warm Ringer's solution or nimodipine)

	(glioma:16-84y,mean age=53.3y)<br><br>(metastasis:24-89y,mean age=61y)			
Shiban et al.(2015)	glioma<br>metastasis<br><br>(32–78y,mean age=53y)	-DCS<br>-mDsCS<br>- Multi-train<br>(p=5, f=500 Hz, pd=0.4ms, si/DCS=14-26mA,si/DsCS≤20 mA)	BB,FCR, thenar, hypothenar,TA	-Halt of resection<br>-Removal of spatulas<br>-Irrigation(warm Ringer's solution)
Lee et al.(2014)	glioma<br>meningioma<br>metastasis<br>craniopharyngi-oma<br>granuloma<br>cavernoma<br><br>(24–80 y,median age=58y)	-TES<br>- Multi-train<br>(p=3–5, ISI=1-3ms,pd=0.5ms, si=200-400 V)	Bilateral:<br>FCR,FCU, thenar, hypothenar,TA,GAST, AH,ADM	-Halt of resection<br>-Release of brain retraction<br>-Restoration of BP
Gempt et al.(2013)	glioma (low/high grade)<br><br>(18–80 y, mean age =56y,SD=16)	-DCS<br>- Multi-train<br>(p=5, f=350 Hz,pd= 0.2-0.3 ms, si=6-30mA)	Contralateral:<br>BB,FCR,APB,ADM,TA	N/A
Ostrý et al.(2013)	solitary lesion compressing or infiltrating the CST<br><br>(19-73y,mean age= 48.8y)	-DCS<br>-mDsCS<br>- Multi-train<br>(p=5 ,f=500 Hz, pd=0.4 ms, si/DCS=5-10% above ST, si/DsCS≤30mA)	Contralateral:<br>BB,APB,ADQ,ED,QUAD, GAST,TA,AH	Resection interruption and subcortical mapping (If threshold increase ≥2 mA)
Pastor et al.(2013)	primary brain tumors<br><br>(mean age= 49.8y, SD=2.4)	-DCS &TES<br>- Multi-train<br>(DCS:p=4–6 ,f= 500 Hz, pd= 0.15ms ,si=4-30mA TES: p=5–6, f=500 Hz, pd=0.05ms,si=100–320 V)	Contralateral:<br>OO, DELT,BB,TRI,FC,ED, thenar,TA,AH,ADM	-Changing of resection area<br>-Halt of resection<br>- Irrigation(warm saline)
Seidel et al.(2013)	intrinsic brain tumors, metasta-ses and vascular lesions not involving the PMC	-DCS<br>- mDsCS<br>- Multi-train<br>(p=5,f=250 Hz ISI=4 ms, pd=0.5 ms, si/DsCS≤22 mA)	Contralateral:<br>face, distal and proximal upper and lower limbs	-Halt of resection(stop of resection if ≥15 min MEP change)<br>-Ruling out technical confounders<br>-Normalizing anesthesia

	(mean age= 50y, SD=17)			-Increase of CPP<br>-Nimodipine<br>-Removal of brain retractor (rarely)
Sakurada et al.(2012)	glioma	-DCS<br>-N/A simulation parameters	N/A	N/A
Senft et al.(2012)	glioma (low/high grade)<br><br>(mean age=47.7y, SD=13.3)	-DCS &TES<br>- Multi-train<br>(p=5, ISI=4ms, pd= 0.5ms)	N/A	-Halt of resection<br>-Irrigation(warm saline)<br>-Reappraisal of surgical strategy
Hatiboglu et al.(2010)	Glioma<br><br>(age range of the 16 patients N/A)	-DCS<br>-Multi-train<br>(p=5, f=500Hz, pd=0.3-0.5 ms, si≤20 mA)	Contralateral: forearm, hand, leg and foot muscles	N/A
Ichikawa et al.(2010)	AVM<br><br>(9-56y)	-DCS<br>-Multi-train<br>(p=5, f=500Hz, pd=0.2ms, si=10-35 mA)	Contralateral: FCR,thenar,TA	-Stop of surgical maneuvers<br>-Hemostasis
Szelényi et al.(2010)	glioma<br>metastasis<br>meningioma<br>hemangioblastoma<br>colloid cyst<br><br>(7-70, mean age=42.8, SD=18.2)	-TES<br>-Multi-train<br>(p=5, ISI=4ms, pd=0.5 ms, si/TES=ST+10-20%ST)	Bilateral:<br>APB, TA<br>Contralateral:<br>BB,ED<br>Sometimes QUAD,AH	-Ruling out technical failures and anesthetic/ systemic confounders<br>-Reappraisal of surgical step<br>-Halt of resection<br>-Replacement of retractor
Kombos et al.(2009)	intrinsic insular tumors<br><br>(23-61y)	-TES<br>-mDsCS<br>- Multi-train<br>(p=5, f=500 Hz,pd=0.3 ms, si/TES≤400 V, si/DsCS≤10mA)	Contralateral: forearm flexor, thenar, QUAD	-Halt of resection<br>-Papaverine
Neuloh et al.(2009)	glioma<br>metastasis<br>cortical dysplasia	-DCS &TES<br>- Multi-train<br>(p=5-7 ,f=250-500Hz, ISI= 2-4 ms, pd= 0.3-1msec<br>DCS /si≤30mA, TES /si≤100mA)	forearm flexor, thenar, hypothenar,TA	-Halt of resection<br>-Cessation of vascular manipulation<br>-Readjustment of brain retractors<br>-Papaverine
Neuloh et al.(2007)	insular glioma<br><br>(9-73y, median age=39y)	-DCS &TES<br>- Multi-train	distal UE muscles<br>distal LE muscles	-Halt of resection<br>-Readjustment of brain retractors<br>-Papaverine

		(p=4-7 ,f=250-500Hz, ISI= 2-4 ms, pd= 0.2-0.7msec<br>DCS /si≤30mA, TES /si≤100mA)		
Suess et al.(2006)	glioma<br>metastasis<br>meningioma<br>cavernoma<br>AVM<br>PNET<br>chondroma<br>epidermoid cyst<br><br>(16–87y, mean age=57.3y)	-DCS<br>- Multi-train<br>(p=5-7,f=400-500 Hz, pd=0.3 ms, si≤25mA)	Contralateral:<br>FCR,FCU,APB,PL, FDS,QUAD,GAST	-Release of spatulas<br>-Stop of electrocoagulation<br>-Check of strip electrode position
Neuloh et al.(2004)	glioma<br>meningioma<br>metastasis<br>AVM<br><br>(11–79 y,median age=35y)	- DCS<br>- Multi-train<br>(p=4-7,f=250-500Hz, ISI= 2-4 ms, pd=0.2-0.7msec si≤30mA)	Contralateral:<br>forearm flexor, thenar, hypothenar, sometimes TA,OO,QUAD	- Exclusion of technical reasons<br>- Increase of stimulus intensity up to maximum(if persisting MEP loss from all muscles in a limb)<br>- Readjustment of brain retractors<br>-Papaverine<br>-AVM: tamponade instead of electrocoagulation, increase of BP
Kombos et al.(2001)	glioma<br>meningioma<br>metastasis<br>AVM<br>epidermoid cyst<br><br>(16–79y,mean age= 56.2y]	-DCS<br>-Multi-train<br>(p=7–10, f=400-500 Hz, pd=0.2-0.7 ms,<br>si= 16.9 ± 7.76 mA)	Contralateral:<br>forearm flexor, thenar, QUAD	- Check of electrode position and equipment<br>-Release of brain retractor
Zhou et al. (2001)	glioma<br>metastasis<br>meningioma<br>ependymoma<br><br>(6-63y, mean age=43, SD=12.9)	-TES<br>-Multi-train<br>(p=5, pd=0.5 ms, si=40–160 mA)	Contralateral:<br>BB,APB,TA,OO<br><br>Ipsilateral:<br>APB(negative control)	-Repeat stimulation with increased si or number of stimuli (train of seven)<br>-Ruling out nonsurgical factors<br>-Cessation of dissection<br>-Reposition of the retractor

Cedzich et al.(1996)	glioma<br>meningioma<br>metastasis<br>angioma<br>cavernoma<br><br>(4-74y, mean age=45.5y)	DCS<br>(p=1-5,f=500 Hz, pd=0.2-0.4ms, si=6-20 mA/ 30-110 V)	Contralateral:<br>forearm flexor, thenar, hypothenar,sometimes TA	N/A
**EPILEPSY SURGERY**				
Koo et al.(2019)	Temporal lobe epilepsy surgery<br><br>(26–47 years, mean age= 33.3y,SD=8.6)	-TES<br>-Multi-train<br>(p=5, ISI=2ms, pd=0.05-0.2 ms, si≤400V)	Bilateral:<br>APB,ADM,TA,AH	-Halt of resection<br>-Release of brain retraction<br>-Increase of BP<br>-Irrigation(warm saline, nimodipine, papaverine)<br>-Reappraisal of surgical strategy
Neuloh et al.(2010)	Intractable focal epilepsy-lesionectomy<br><br>(2-66 y,median age= 25.5y)	-DCS &TES<br>-Multi-train<br>(p=5-7, ISI= 2-4 ms, pd=0.3-1 ms, DCS/si≤30mA, TES /si≤100 mA)	Distal limb muscles	Technical and surgical troubleshooting
**ANEURYSM CLIPPING**				
Guo et al. (2021)	MCA aneurysms<br><br>(14-77y,mean age=53.93y, SD= 10.75)	-TES<br>-Multi-train<br>(p=8,f=250-500Hz, ISI=0.05-0.5ms, si=100-400V)	APB,AH	- Exclusion of anesthetic/physiological effects<br>-Release of temporary clip<br>-Reposition of permanent clip
Park et al.(2021)	ACA,ICA, MCA, AChA AcoA,PcoA aneurysms<br><br>(mean age=61.27y, SD=9.04)	-TES<br>-Multi-train<br>(p=5,ISI=1-4ms, pd=0.5 ms,si=200–350 V)	Bilateral:<br>APB,FCR,TA,AH	-Release of temporary clip<br>-Release / reposition of permanent clip<br>-Relief of brain retractor<br>- Ruling out changes of BP, BT and anesthesia
You et al.(2021)	ICA aneurysms<br><br>(mean age=53.7y, SD=10.6)	- TES<br>-Multi-train<br>(p=5-8 ,f=250-500Hz, pd=0.05-0.5ms,si=100-400V)	Bilateral:<br>APB,AH	N/A

Kameda et al.(2020)	MCA aneurysms<br><br>(age:N/A)	-DCS & TES<br>-Multi-train<br>(p=5, pd= 0.5ms, si=20% above ST)	Bilateral:<br>thenar	-Release of temporary clip<br>-Reposition of permanent clip
Byoun et al.(2019)	AChA aneurysms<br><br>(mean age=54.66y, SD=9.12)	-TES<br>-Multi-train<br>(p=5,ISI=2-4ms, pd=0.05 ms,si=250-500V)	Contralateral:<br>APB, ADM,TA,AH	Increase of stimulation intensity within 30% of baseline
Greve et al.(2019)	ACA,ICA, MCA Aneurysms<br><br>(20–77y,mean age=57y,SD= 11)	-TES<br>-Multi-train<br>(p=4-5, ISI= 2-4msec, pd=0.5ms)	Bilateral:<br>APB,TA	Technical, surgical, and anesthesiological troubleshooting
Li et al.(2019)	Anterior and posterior circulation aneurysms<br><br>(18–77y, mean age=54y)	-TES<br>-Multi-train<br>(p=8, pd=0.5-1ms, ISI=2-5ms,si= 100–400 V)	Bilateral:<br>APB,AH	-Loosening the clip<br>-Unblocking the occlusion
Choi et al.(2017)	ACA, AchA,MCA, PCA,PcoA aneurysms<br><br>(27–86y,mean age=57.5y,SD= 9.3)	-TES<br>- Multi-train<br>at 300-350V	Bilateral:<br>DELT,thenar,TA,AH	-Stop of dissection<br>-Relief of brain retractor<br>-Release / reposition of aneurysm clips
Komatsu et al. (2017)	AChA,ICA, LSA.MCA aneurysms (temporal occlusion of the parent artery)	-DCS<br>-si=5-15mA	Bilateral:<br>APB,BB,DELT QUAD,GAST,TA	Release of temporary clip
Staarman et al.(2017)	ACA,ICA, MCA,PCA AChA AcoA,PcoA, ophthalmic, BA, VA, SCA, PICA aneurysms	-TES<br>-Multi-train<br>(p≤7, pd=0.3ms, si=30-125V)	APB, ADM,FCU, GAST,TA,AH	N/A
Kim et al.(2016)	Cerebral aneurysms<br><br>(26–82y, mean age=56.8y)	-TES<br>-Multi-train<br>(p=6, pd=0.5ms, ISI=3ms,si= 265.6±72.67 V)	Bilateral:<br>APB,TA,AH	Reposition of clip
Maruta et al.(2016)	ACA, ICA, MCA, AcoA aneurysms<br><br>30-89y, mean age=61.1y, SD=11.1)	-DCS & TES<br>-Multi-train<br>(p=5, pd=0.2-0.5ms, f=500Hz,si/DCS=ST+2mA si/TES= ST+2V)	APB,SOL,TA,AH	N/A

Song et al.(2015)	Giant intracranial ICA aneurysms<br><br>(24–60y, mean age=44y,SD=12.1)	-TES<br>-Multi-train (p=5, pd=0.1ms, ISI=2ms,si= 300-400V)	Bilateral:<br>APB,AH<br><br>*ipsilateral as negative control	-Release of temporary clip<br>-Increase of BP
Sasaki et al.(2014)	AchA,LSA,MCA aneurysms<br><br>(age:N/A)	-DCS & TES<br>-N/A simulation parameters	N/A	Reposition of clip
Takebayashi et al. (2014)	ICA, MCA large and giant aneurysms<br><br>(14-79 years (mean age=53.2y, SD= 8.9)	-DCS<br>-Multi-train<br>(p=5, f=500Hz,pd=0.2ms)	Contralateral:<br>thenar	-Correction of clip position<br>-Readjustment of retractors<br>-Increase of BP<br>-Papaverine
Yue et al.(2014)	MCA aneurysms<br><br>(17-67y, mean age=48.1y,SD=10.6)	-TES<br>-Multi-train<br>(p=5, ISI=2ms,pd=0.1ms, si=100-400V)	Bilateral:<br>APB,AH	-Relief of brain retractor<br>-Release of temporary clip<br>-Reposition of permanent clip
Dengler et al.(2013)	EC-IC bypass surgery (giant intracranial aneurysms)<br><br>(14-76 y, mean age=50.2y)	-TES<br>-Multi-train<br>(p=5, f=500Hz,pd=0.3ms, si≤400V)	Contralateral:<br>forearm flexor, thenar, TA	-Ruling out nonsurgical factors<br>-Release of temporary clip<br>-Increasing the speed of suturing<br>-Increase of BP
Kang et al.(2013)	ACA, ICA, MCA, AcoA,PcoA aneurysms<br><br>(33-74y, mean age=48y,SD=8.7y)	-TES<br>- Multi-train (p=5, ISI=2 ms, pd=0.2ms)	Contralateral:<br>BR,APB,TA,AH	-Nimodipine<br>-Papaverine
Maruta et al.(2012)	ACA, ICA, MCA, AcoA aneurysms (3 meningiomas, 1GBM)<br><br>(40-85y, mean age= 63y,SD= 10.8)	-DCS & TES<br>- Multi-train<br>(p=5,pd=0.5-1ms,ISI=2ms,si=ST+2mA and ≤30mA)	SOL,TA,AH	N/A
Shi et al.(2012)	ACA, ICA, MCA, PCA,AcoA,PcoA, aneurysms	-TES<br>Multi-train (p=3-7,pd=0.05,ISI=1-2ms,si=100-400V)	OO,APB,AH	-Adjustment of temporary clip<br>-Papaverine<br>-Relief of brain retractor

Motoyama et al. (2011)	ACA, ICA, MCA aneurysms<br><br>(42-79y, mean age= 62.1y,SD= 8.9)	-DCS & TES<br>- Multi-train<br>(p=5, ISI=2ms, si/TES= ST +20V, si/DCS= ST +2 mA and ≤30mA)	Bilateral:<br>APB	Reposition/Release of the clip
Irie et al. (2010)	ICA,MCA,AchA, PCoA,ophthalmic/ paraclinoid artery aneurysms<br><br>(mean age=66y, SD=11)	-TES<br>- Multi-train<br>(p= 5, ISI=0.5 ms, si=500 V)	Bilateral:<br>APB,thenar	Reposition/ Release of the clip
Yeon et al.(2010)	ACA,ICA, MCA, AChA AcoA,PcoA aneurysms<br><br>(mean age=55y)	-TES<br>- Multi-train<br>(p=5, ISI=2ms, si=300–400 V)	Bilateral:<br>APB,ADM,TA,AH	N/A
Szelényi et al.(2007)	ACA,ICA, MCA, AcoA, posterior circulation aneurysms<br><br>(mean age=51.5y, SD=14.7)	-DCS & TES<br>-Multi-train<br>(p=5, ISI=4ms, pd=0.5 ms, si/TES=ST+5%ST, si/DCS≤ 25mA)	Bilateral:<br>APB, TA,AH<br><br>Contralateral:<br>BB,ED	-Ruling out non-surgical confounders by recording TES-MEPs from both hemispheres<br>-Reappraisal of surgical strategy
Weinzierl et al.(2007)	ICA, MCA, PcoA aneurysms<br><br>(age range of the 18 aneurysm-patients N/A)	-TES<br>-Multi-train<br>(p=4, ISI=2ms, pd=0.2 ms, si=100-700 V)	Bilateral:<br>APB, TA	-Ruling out hemodynamic/systemic factors and technical confounders<br>-Release of temporary clip<br>-Release of retraction<br>-Reposition of permanent clip
Szelényi et al.(2006)	ACA, ICA, MCA, PCA,AcoA,PcoA, AchA, ophthalmic Artery,BA, PICA,SCA aneurysms<br><br>(9-85y, mean age=51.2y, SD=15.8)	-DCS & TES<br>-Multi-train<br>(p=5, ISI=4ms, pd=0.5 ms, si/TES≤ 240 mA, si/DCS≤ 33mA)	Bilateral:<br>APB, TA<br><br>Contralateral:<br>BB,ED	-Reposition of brain retractor<br>-After permanent clip: Doppler U/S, reposition of clip<br>-After temporary clip: clip reopening, duration of clipping as short as possible<br>-DCS MEP loss: Ruling out strip electrode displacement and

				attempt to elicit MEP with TES
Horiuchi et al.(2005)	LSA and MCA aneurysms<br><br>(age N/A for all patients)	-DCS<br>-Multi-train (p=5, f=500 Hz,pd=0.2 ms, si=ST+2mA)	Contralateral: thenar	N/A
Suzuki et al. (2003)	ICA(AchA, MCA,PcoA) aneurysms<br><br>(age N/A for all patients)	-DCS<br>- Multi-train (p=5, f=500 Hz, pd=0.2ms,si= ST+2mA)	Contralateral: thenar	Adjustment of surgical maneuvers
**ENDOVASCULAR PROCEDURES FOR ANEURYSMS**				
Nakagawa et al.(2020)	Anterior and posterior circulation aneurysms<br><br>(mean age=61y, SD=14)	-TES<br>-Multi-train (p=5, ISI=2ms, pd= 1.5-2 ms., si=supramaximal)	Bilateral:<br>APB, TA, GAST,AH	-Balloon deflation<br>-Guiding catheter reposition<br>-Intraarterial antithrombotic drug injection<br>-Coil retrieval
Wilent et al.(2020)	aneurysm<br>AVM<br>fistula<br>acute ischemic stroke<br>stenosis<br>vasospasm<br><br>(average patient age=57.1y)	-TES<br>-N/A simulation parameters	N/A	-Adjustment of instruments<br>-Increase of BP<br>-Active hemostasis<br>-Thrombolysis/ Thrombectomy
Lee et al.(2019)	Cerebral artery aneurysms<br>AVM<br>Arteriovenous/ Carotid-cavernous fistula<br>Stenosis<br><br>(11–87y, mean age=59.5y)	-TES<br>-Multi-train (p=8, ISI=0.75ms,si=350-450 V)	Bilateral:<br>APB,QUAD,TA,AH	N/A
Piñeiro et al.(2015)	ICA,MCA,BA aneurysms<br>AVM<br><br>(38-74y)	-TES<br>-Multi-train (p=5-7,ISI=4ms, pd=0.5ms,si≤300V)	Bilateral:<br>APB,TA,AH	-Stop embolization<br>- Optimizing hemodynamic/systemic conditions

Hiraishi et al.(2011)	AChA aneurysms	-TES<br>-Multi-train<br>(p=5, ISI=2ms,si=230-550 V)	BR, APB, AT,AH	Extraction of the coils

Abbreviations:
ACA: Anterior Cerebral Artery ; **AChA**: Anterior Choroidal Artery ; **AcoA**: Anterior Communicating Artery ; **ADM:**Abductor Digiti Minimi **; ADQ:** Abductor Digiti Quinti ; **AH**:Abductor Hallucis ; **APB:** Abductor Pollicis Brevis ; **AVM**: Arteriovenous Malformation ; **BA:** Basilar Artery ; **BB**:Brachial Biceps ; **bDsCS**: bipolar Direct Subcortical Simulation ; **BP**:Blood Pressure **; BR:**Brachioradialis ; **BT:**Body Temperature ; **CPP**: Cerebral Perfusion Pressure ; **DCS**: Direct Cortical Stimulation; **DELT**:Deltoid ; **DNET:** Dysembryoplastic Neuroepithelial Tumor **ED**:Extensor Digitorum ; **EDB:**Extensor Digitorum Brevis ; **FC:** Flexor Carpi ; **FCR**: Flexor Carpi Radialis ; **FCU:** Flexor Carpi Ulnaris ; **FDS**: Flexor Digitorum Superficialis **; f:**frequency ; **GAST:**Gastrocnemius ; **ICA:**Internal Carotid Artery ; **ISI**: Interstimulus Interval ; **LE**:Lower Extremities ; **LSA:** Lenticulostriate Artery ; **MCA**:Middle Cerebral Artery ; **mDsCS**:monopolar Direct Subcortical Simulation **; MEP:** Motor Evoked Potential ; **N/A:** Not available ; **OO:** Orbicularis Oris ; **p**:Number of pulses ; **PcoA**: Posterior Communicating Artery**; pd:**pulse duration ; **PICA:**Posterior Inferior Cerebellar Artery ; **PL:** Palmaris Longus ; **PMC:**Primary Motor Cortex **; PNET**: Primitive Neuroectodermal Tumor ;**QUAD**:Quadriceps Femoris ; **SCA**:Superior Cerebellar Artery ; **SD:**Standard Deviation ; **SOL:**Soleus ; **ST**: Stimulation Threshold ; **TA:**Tibialis Anterior ; **TES:**Transcranial Electrical Stimulation ; **TRI**:Triceps ; **UE:**Upper Extremities ; **U/S:** ultrasonography ; **VA:**Vertebral Artery ; **y**:years

Supplementary Figure S1: Forest plot of sensitivity and specificity estimates for all motor deficits

Study	TP	FP	FN	TN	Sensitivity (95% CI)	Specificity (95% CI)
Abboud et al.(2016),TES/TH20%i	13	0	0	80	1.00 [0.75, 1.00]	1.00 [0.95, 1.00]
Abboud et al.(2019),TES/AR50%	20	16	4	212	0.83 [0.63, 0.95]	0.93 [0.89, 0.96]
Abboud et al.(2019),TES/TH20%i	22	3	0	227	1.00 [0.85, 1.00]	0.99 [0.96, 1.00]
Boex et al.(2016),DCS,TES/AR50%	16	0	3	72	0.84 [0.60, 0.97]	1.00 [0.95, 1.00]
Byoun et al.(2019),TES/AR50%	0	0	3	112	0.00 [0.00, 0.71]	1.00 [0.97, 1.00]
Cedzich et al.(1996),DCS/MEP loss	3	3	6	13	0.33 [0.07, 0.70]	0.81 [0.54, 0.96]
Choi et al. (2017),TES/AR50%, LAT10%	3	7	5	371	0.38 [0.09, 0.76]	0.98 [0.96, 0.99]
Dengler et al.(2013),TES/AR50%,LAT10%	1	0	1	32	0.50 [0.01, 0.99]	1.00 [0.89, 1.00]
Gempt et al.(2013),DCS/AR50%	13	0	10	47	0.57 [0.34, 0.77]	1.00 [0.92, 1.00]
Giampiccolo et al.(2021),DCS/AR50%,Lower limbs	8	6	13	31	0.38 [0.18, 0.62]	0.84 [0.68, 0.94]
Giampiccolo et al.(2021),DCS/AR50%,Upper limbs	19	7	44	55	0.30 [0.19, 0.43]	0.89 [0.78, 0.95]
Gogos et al.(2020),DCS,TES/AR20%	3	3	3	50	0.50 [0.12, 0.88]	0.94 [0.84, 0.99]
Gogos et al.(2020)/MT5mA	3	15	3	38	0.50 [0.12, 0.88]	0.72 [0.58, 0.83]
Greve et al.(2019),TES/AR50%	1	4	8	125	0.11 [0.00, 0.48]	0.97 [0.92, 0.99]
Guo et al.(2021),TES/AR50%	11	3	21	250	0.34 [0.19, 0.53]	0.99 [0.97, 1.00]
Hatiboglu et al.(2010),DCS/MEP loss	3	1	5	7	0.38 [0.09, 0.76]	0.88 [0.47, 1.00]
Hiraishi et al.(2011),TES/AR50%	0	0	1	6	0.00 [0.00, 0.97]	1.00 [0.54, 1.00]
Horiuchi et al.(2005),DCS/AR50%	1	0	3	49	0.25 [0.01, 0.81]	1.00 [0.93, 1.00]
Ichikawa et al.(2010),DCS/AR50%	1	0	2	18	0.33 [0.01, 0.91]	1.00 [0.81, 1.00]
Irie et al.(2010),TES/AR50%,TH20mA	1	0	7	102	0.13 [0.00, 0.53]	1.00 [0.96, 1.00]
Kang et al.(2013),TES/AR50%	3	5	0	29	1.00 [0.29, 1.00]	0.85 [0.69, 0.95]
Kim et al.(2016),TES/AR50%	6	7	30	642	0.17 [0.06, 0.33]	0.99 [0.98, 1.00]
Kombos et al.(2001),DCS/AR80%,LAT15%	2	0	6	62	0.25 [0.03, 0.65]	1.00 [0.94, 1.00]
Kombos et al.(2009),TES/AR80%,LAT30%	0	0	2	13	0.00 [0.00, 0.84]	1.00 [0.75, 1.00]
Koo et al.(2019),TES/AR50%	0	0	2	277	0.00 [0.00, 0.84]	1.00 [0.99, 1.00]
Lee et al.(2014),TES/AR50%, LAT10%	3	4	11	66	0.21 [0.05, 0.51]	0.94 [0.86, 0.98]
Lee et al.(2019),TES/AR50%,LAT10%	1	1	3	556	0.25 [0.01, 0.81]	1.00 [0.99, 1.00]
Li et al.(2019),TES/AR50%	15	1	34	42	0.31 [0.18, 0.45]	0.98 [0.88, 1.00]
Majchrzak et al.(2018),TES/AR50%	11	0	7	17	0.61 [0.36, 0.83]	1.00 [0.80, 1.00]
Maruta et al.(2012),DCS,TES/AR50%	0	0	3	19	0.00 [0.00, 0.71]	1.00 [0.82, 1.00]
Maruta et al.(2016),DCS,TES/AR50%	2	5	16	220	0.11 [0.01, 0.35]	0.98 [0.95, 0.99]
Moiyadi et al.(2018), DCS/AR30-50%	1	0	6	19	0.14 [0.00, 0.58]	1.00 [0.82, 1.00]
Moiyadi et al.(2018), TES/AR50-80%	1	2	5	31	0.17 [0.00, 0.64]	0.94 [0.80, 0.99]
Motoyama et al.(2011),DCS,TES/AR50%	1	0	9	43	0.10 [0.00, 0.45]	1.00 [0.92, 1.00]
Nakagawa et al.(2020),TES/AR50%	3	0	7	154	0.30 [0.07, 0.65]	1.00 [0.98, 1.00]
Neuloh et al.(2004),DCS/AR50%, LAT10%	21	5	40	81	0.34 [0.23, 0.48]	0.94 [0.87, 0.98]
Neuloh et al.(2007),DCS,TES/AR50%	15	0	12	61	0.56 [0.35, 0.75]	1.00 [0.94, 1.00]
Neuloh et al.(2009),DCS,TES/AR50%	23	10	29	129	0.44 [0.30, 0.59]	0.93 [0.87, 0.96]
Neuloh et al.(2010),DCS,TES/AR50%, LAT10-15%	7	4	4	71	0.64 [0.31, 0.89]	0.95 [0.87, 0.99]
Obermueller et al.(2015),DCS/AR50%	13	12	30	103	0.30 [0.17, 0.46]	0.90 [0.82, 0.94]
Ostry et al.(2013)/ MT5mA	4	6	4	11	0.50 [0.16, 0.84]	0.65 [0.38, 0.86]
Ostry et al.(2013)/MT5mA+ MEP change	2	0	6	17	0.25 [0.03, 0.65]	1.00 [0.80, 1.00]
Park et al.(2021),TES,AR50%	4	0	6	309	0.40 [0.12, 0.74]	1.00 [0.99, 1.00]
Pastor et al.(2013),DCS,TES/AR50%,LAT10%	7	11	1	11	0.88 [0.47, 1.00]	0.50 [0.28, 0.72]
Pineiro et al.(2015),TES/AR50%	1	0	2	5	0.33 [0.01, 0.91]	1.00 [0.48, 1.00]
Plans et al.(2017),DCS/AR50%	8	1	1	82	0.89 [0.52, 1.00]	0.99 [0.93, 1.00]
Plans et al.(2017),DCS/TH5mA	4	8	9	71	0.31 [0.09, 0.61]	0.90 [0.81, 0.96]
Plans et al.(2017)/MT5mA	11	19	6	56	0.65 [0.38, 0.86]	0.75 [0.63, 0.84]
Plans et al.(2017)/MT5mA +AR50%	7	0	6	79	0.54 [0.25, 0.81]	1.00 [0.95, 1.00]
Plans et al.(2017)/MT5mA+ TH5mA	2	0	11	79	0.15 [0.02, 0.45]	1.00 [0.95, 1.00]
Sakurada et al.(2012),DCS/AR50%	2	0	5	23	0.29 [0.04, 0.71]	1.00 [0.85, 1.00]
Sasaki et al.(2014),DCS,TES/MEP loss	2	0	4	171	0.33 [0.04, 0.78]	1.00 [0.98, 1.00]
Seidel et al.(2013),DCS/TH4mA	11	1	19	69	0.37 [0.20, 0.56]	0.99 [0.92, 1.00]
Seidel et al.(2013)/MT3mA	8	17	20	53	0.29 [0.13, 0.49]	0.76 [0.64, 0.85]
Seidel et al.(2013)/MT3mA+ TH4mA	5	0	23	70	0.18 [0.06, 0.37]	1.00 [0.95, 1.00]
Seidel et al.(2020)/MT3mA	30	28	26	98	0.54 [0.40, 0.67]	0.78 [0.70, 0.85]
Senft et al.(2012),DCS,TES/AR50%, TH3mA,TH20mA	4	3	7	40	0.36 [0.11, 0.69]	0.93 [0.81, 0.99]
Shiban et al.(2015),DCS/AR50%	2	0	3	9	0.40 [0.05, 0.85]	1.00 [0.66, 1.00]
Shiban et al.(2015)/MT3mA	0	0	5	9	0.00 [0.00, 0.52]	1.00 [0.66, 1.00]
Song et al.(2015),TES/AR50%	1	0	2	8	0.33 [0.01, 0.91]	1.00 [0.63, 1.00]
Staarman et al.(2017),TES/AR50%	1	0	2	130	0.33 [0.01, 0.91]	1.00 [0.97, 1.00]
Suess et al.(2006),DCS/AR50%,LAT5%,PW30%	17	3	18	94	0.49 [0.31, 0.66]	0.97 [0.91, 0.99]
Suzuki et al.(2003),DCS/AR50%	1	0	4	103	0.20 [0.01, 0.72]	1.00 [0.96, 1.00]
Szelenyi et al.(2006),DCS,TES/AR50%	2	0	13	99	0.13 [0.02, 0.40]	1.00 [0.96, 1.00]
Szelenyi et al.(2006),DCS,TES/TH20mA	1	0	14	99	0.07 [0.00, 0.32]	1.00 [0.96, 1.00]
Szelenyi et al.(2007),DCS,TES/AR50%,TH2mA,TH20mA	5	9	0	85	1.00 [0.48, 1.00]	0.90 [0.83, 0.96]
Szelenyi et al.(2010),TES/AR50%,TH20mA,TH100V	11	4	4	9	0.73 [0.45, 0.92]	0.69 [0.39, 0.91]
Takebayashi et al. (2014),DCS/MEP loss	4	0	6	40	0.40 [0.12, 0.74]	1.00 [0.91, 1.00]
Weinzierl et al.(2007),TES/AR50%,LAT10%	0	3	0	15	Not estimable	0.83 [0.59, 0.96]
Wilent et al.(2020),TES/AR40%	12	24	1	726	0.92 [0.64, 1.00]	0.97 [0.95, 0.98]
Yeon et al.(2010),TES/AR50%	0	0	1	97	0.00 [0.00, 0.97]	1.00 [0.96, 1.00]
You et al.(2021),TES/AR50%	3	2	14	119	0.18 [0.04, 0.43]	0.98 [0.94, 1.00]
Yue et al.(2014),TES/AR50%	1	0	3	39	0.25 [0.01, 0.81]	1.00 [0.91, 1.00]
Zhou et al.(2001),TES/AR50%	8	0	0	42	1.00 [0.63, 1.00]	1.00 [0.92, 1.00]
Zhou et al.(2017),DCS,TES/AR80%	6	0	4	60	0.60 [0.26, 0.88]	1.00 [0.94, 1.00]

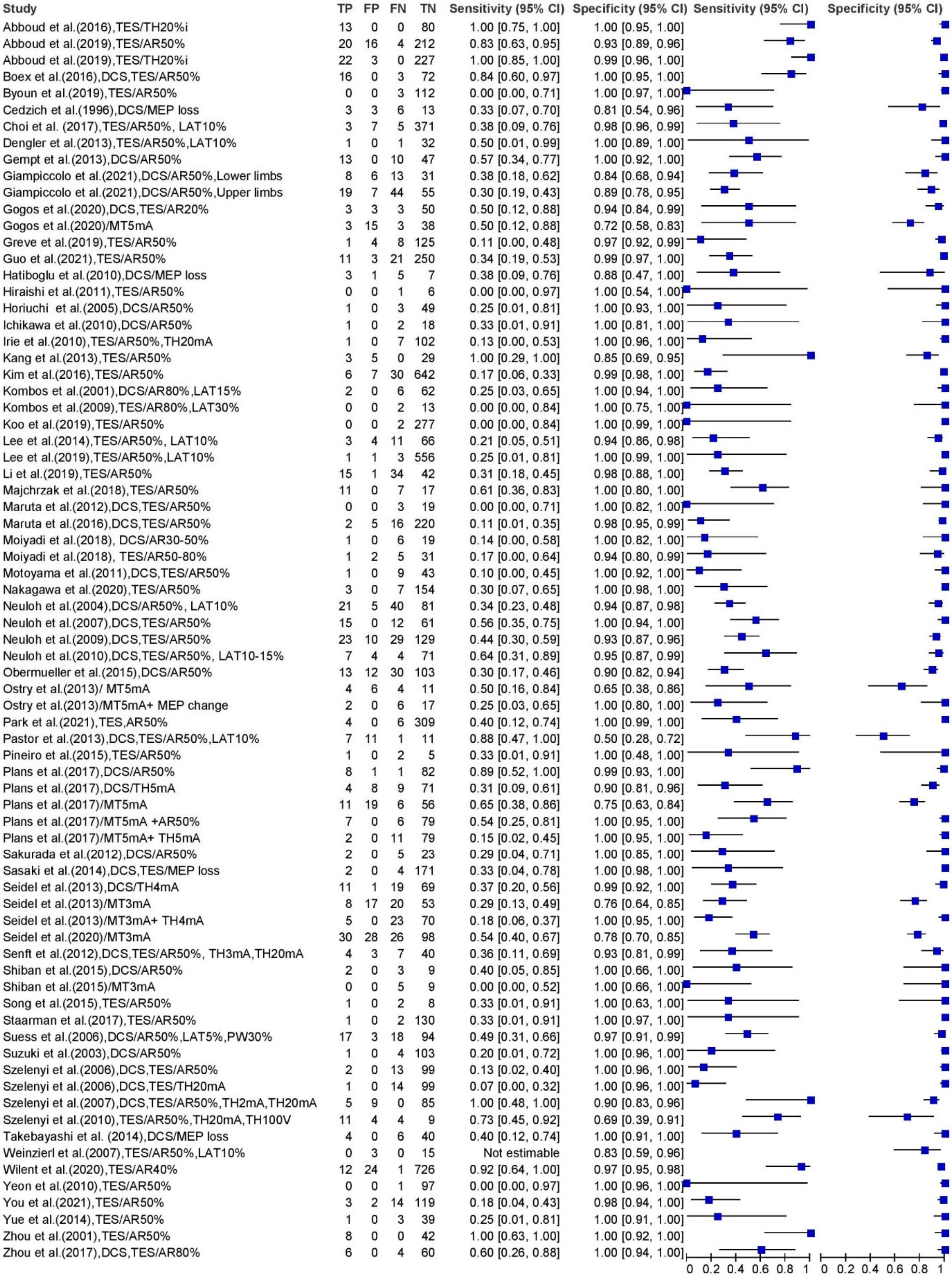

Supplementary Figure S2: Forest plot of sensitivity and specificity estimates for transient motor deficits

Study	TP	FP	FN	TN	Sensitivity (95% CI)	Specificity (95% CI)
Abboud et al.(2016),TES/TH20%i	0	13	0	80	Not estimable	0.86 [0.77, 0.92]
Abboud et al.(2019),TES/AR50%	6	30	1	215	0.86 [0.42, 1.00]	0.88 [0.83, 0.92]
Abboud et al.(2019),TES/TH20%i	7	18	0	227	1.00 [0.59, 1.00]	0.93 [0.89, 0.96]
Boex et al.(2016),DCS,TES/AR50%	3	13	2	73	0.60 [0.15, 0.95]	0.85 [0.76, 0.92]
Byoun et al.(2019),TES/AR50%	0	0	0	115	Not estimable	1.00 [0.97, 1.00]
Cedzich et al.(1996),DCS/MEP loss	0	6	4	15	0.00 [0.00, 0.60]	0.71 [0.48, 0.89]
Choi et al. (2017),TES/AR50%, LAT10%	1	9	1	375	0.50 [0.01, 0.99]	0.98 [0.96, 0.99]
Dengler et al.(2013),TES/AR50%,LAT10%	0	1	1	32	0.00 [0.00, 0.97]	0.97 [0.84, 1.00]
Gempt et al.(2013),DCS/AR50%	5	8	8	49	0.38 [0.14, 0.68]	0.86 [0.74, 0.94]
Giampiccolo et al.(2021),DCS/AR50%,Lower limbs	3	11	0	44	1.00 [0.29, 1.00]	0.80 [0.67, 0.90]
Giampiccolo et al.(2021),DCS/AR50%,Upper limbs	6	20	16	83	0.27 [0.11, 0.50]	0.81 [0.72, 0.88]
Gogos et al.(2020),DCS,TES/AR20%	2	4	2	51	0.50 [0.07, 0.93]	0.93 [0.82, 0.98]
Gogos et al.(2020)/MT5mA	2	16	2	39	0.50 [0.07, 0.93]	0.71 [0.57, 0.82]
Greve et al.(2019),TES/AR50%	0	5	5	128	0.00 [0.00, 0.52]	0.96 [0.91, 0.99]
Guo et al.(2021),TES/AR50%	1	13	8	263	0.11 [0.00, 0.48]	0.95 [0.92, 0.97]
Horiuchi et al.(2005),DCS/AR50%	0	1	3	49	0.00 [0.00, 0.71]	0.98 [0.89, 1.00]
Kombos et al.(2009),TES/AR80%,LAT30%	0	0	2	13	0.00 [0.00, 0.84]	1.00 [0.75, 1.00]
Koo et al.(2019),TES/AR50%	0	0	2	277	0.00 [0.00, 0.84]	1.00 [0.99, 1.00]
Lee et al.(2014),TES/AR50%, LAT10%	0	7	8	69	0.00 [0.00, 0.37]	0.91 [0.82, 0.96]
Li et al.(2019),TES/AR50%	2	1	19	42	0.10 [0.01, 0.30]	0.98 [0.88, 1.00]
Majchrzak et al.(2018),TES/AR50%	1	10	6	18	0.14 [0.00, 0.58]	0.64 [0.44, 0.81]
Maruta et al.(2012),DCS,TES/AR50%	0	0	3	19	0.00 [0.00, 0.71]	1.00 [0.82, 1.00]
Maruta et al.(2016),DCS,TES/AR50%	0	7	7	229	0.00 [0.00, 0.41]	0.97 [0.94, 0.99]
Moiyadi et al.(2018), DCS/AR30-50%	1	0	2	23	0.33 [0.01, 0.91]	1.00 [0.85, 1.00]
Moiyadi et al.(2018), TES/AR50-80%	0	3	4	32	0.00 [0.00, 0.60]	0.91 [0.77, 0.98]
Neuloh et al.(2004),DCS/AR50%, LAT10%	9	17	38	83	0.19 [0.09, 0.33]	0.83 [0.74, 0.90]
Neuloh et al.(2007),DCS,TES/AR50%	7	8	12	61	0.37 [0.16, 0.62]	0.88 [0.78, 0.95]
Neuloh et al.(2009),DCS,TES/AR50%	12	21	25	133	0.32 [0.18, 0.50]	0.86 [0.80, 0.91]
Neuloh et al.(2010),DCS,TES/AR50%, LAT10-15%	2	9	4	71	0.33 [0.04, 0.78]	0.89 [0.80, 0.95]
Obermueller et al.(2015),DCS/AR50%	2	23	22	111	0.08 [0.01, 0.27]	0.83 [0.75, 0.89]
Ostry et al.(2013)/ MT5mA	3	7	4	11	0.43 [0.10, 0.82]	0.61 [0.36, 0.83]
Ostry et al.(2013)/MT5mA+ MEP change	2	0	6	17	0.25 [0.03, 0.65]	1.00 [0.80, 1.00]
Park et al.(2021),TES,AR50%	2	2	2	313	0.50 [0.07, 0.93]	0.99 [0.98, 1.00]
Pastor et al.(2013),DCS,TES/AR50%,LAT10%	4	14	1	11	0.80 [0.28, 0.99]	0.44 [0.24, 0.65]
Sakurada et al.(2012),DCS/AR50%	0	2	2	26	0.00 [0.00, 0.84]	0.93 [0.76, 0.99]
Sasaki et al.(2014),DCS,TES/MEP loss	0	2	4	171	0.00 [0.00, 0.60]	0.99 [0.96, 1.00]
Seidel et al.(2013),DCS/TH4mA	4	8	2	86	0.67 [0.22, 0.96]	0.91 [0.84, 0.96]
Seidel et al.(2013)/MT3mA	2	23	4	69	0.33 [0.04, 0.78]	0.75 [0.65, 0.83]
Seidel et al.(2013)/MT3mA+ TH4mA	2	3	4	89	0.33 [0.04, 0.78]	0.97 [0.91, 0.99]
Seidel et al.(2020)/MT3mA	17	41	6	115	0.74 [0.52, 0.90]	0.74 [0.66, 0.80]
Senft et al.(2012),DCS,TES/AR50%, TH3mA,TH20mA	2	5	5	42	0.29 [0.04, 0.71]	0.89 [0.77, 0.96]
Shiban et al.(2015),DCS/AR50%	1	1	3	9	0.25 [0.01, 0.81]	0.90 [0.55, 1.00]
Shiban et al.(2015)/MT3mA	0	0	4	9	0.00 [0.00, 0.60]	1.00 [0.66, 1.00]
Suess et al.(2006),DCS/AR50%,LAT5%,PW30%	0	20	16	196	0.00 [0.00, 0.21]	0.91 [0.86, 0.94]
Suzuki et al.(2003),DCS/AR50%	0	1	4	103	0.00 [0.00, 0.60]	0.99 [0.95, 1.00]
Szelenyi et al.(2006),DCS,TES/AR50%	0	2	9	103	0.00 [0.00, 0.34]	0.98 [0.93, 1.00]
Szelenyi et al.(2006),DCS,TES/TH20mA	0	1	9	104	0.00 [0.00, 0.34]	0.99 [0.95, 1.00]
Szelenyi et al.(2007),DCS,TES/AR50%,TH2mA,TH20mA	3	11	0	85	1.00 [0.29, 1.00]	0.89 [0.80, 0.94]
Takebayashi et al. (2014),DCS/MEP loss	0	4	6	40	0.00 [0.00, 0.46]	0.91 [0.78, 0.97]
Weinzierl et al.(2007),TES/AR50%,LAT10%	0	3	0	15	Not estimable	0.83 [0.59, 0.96]
Yeon et al.(2010),TES/AR50%	0	0	1	97	0.00 [0.00, 0.97]	1.00 [0.96, 1.00]
Yue et al.(2014),TES/AR50%	0	1	0	42	Not estimable	0.98 [0.88, 1.00]
Zhou et al.(2017),DCS,TES/AR80%	5	1	4	60	0.56 [0.21, 0.86]	0.98 [0.91, 1.00]

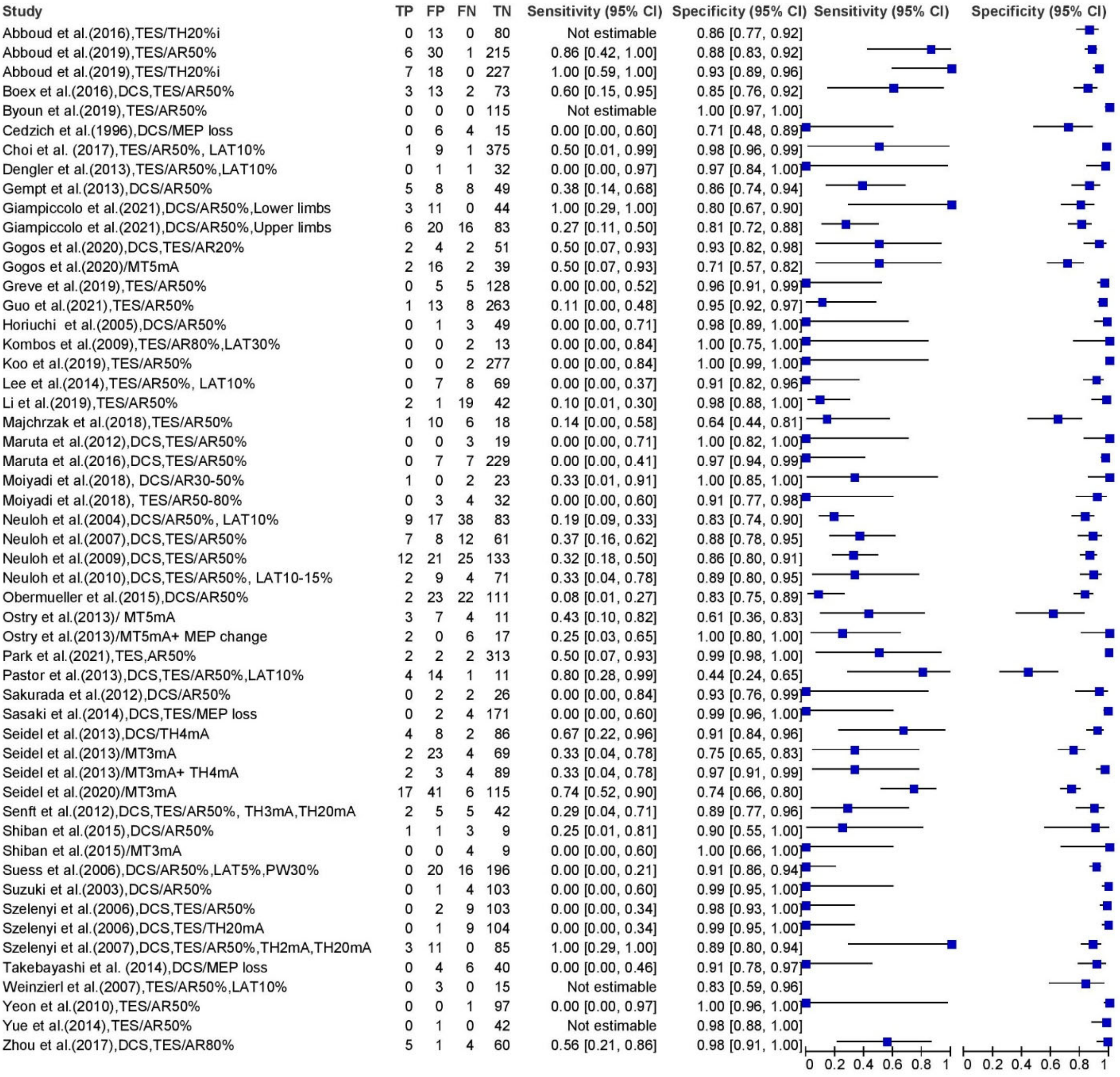

Supplementary Figure S3: Forest plot of sensitivity and specificity estimates for early-transient motor deficits

Study	TP	FP	FN	TN	Sensitivity (95% CI)	Specificity (95% CI)
Abboud et al.(2016),TES/TH20%i	8	5	0	80	1.00 [0.63, 1.00]	0.94 [0.87, 0.98]
Abboud et al.(2019),TES/AR50%	6	30	3	213	0.67 [0.30, 0.93]	0.88 [0.83, 0.92]
Abboud et al.(2019),TES/TH20%i	7	18	0	227	1.00 [0.59, 1.00]	0.93 [0.89, 0.96]
Boex et al.(2016),DCS,TES/AR50%	5	11	1	74	0.83 [0.36, 1.00]	0.87 [0.78, 0.93]
Giampiccolo et al.(2021),DCS/AR50%,Lower limbs	2	12	3	41	0.40 [0.05, 0.85]	0.77 [0.64, 0.88]
Giampiccolo et al.(2021),DCS/AR50%,Upper limbs	3	23	16	83	0.16 [0.03, 0.40]	0.78 [0.69, 0.86]
Hatiboglu et al.(2010),DCS/MEP loss	1	3	5	7	0.17 [0.00, 0.64]	0.70 [0.35, 0.93]
Hiraishi et al.(2011),TES/AR50%	0	0	1	6	0.00 [0.00, 0.97]	1.00 [0.54, 1.00]
Ichikawa et al.(2010),DCS/AR50%	0	1	2	18	0.00 [0.00, 0.84]	0.95 [0.74, 1.00]
Irie et al.(2010),TES/AR50%,TH20mA	1	0	7	102	0.13 [0.00, 0.53]	1.00 [0.96, 1.00]
Kang et al.(2013),TES/AR50%	3	5	0	29	1.00 [0.29, 1.00]	0.85 [0.69, 0.95]
Lee et al.(2019),TES/AR50%,LAT10%	1	1	3	556	0.25 [0.01, 0.81]	1.00 [0.99, 1.00]
Moiyadi et al.(2018), DCS/AR30-50%	0	1	4	21	0.00 [0.00, 0.60]	0.95 [0.77, 1.00]
Moiyadi et al.(2018), TES/AR50-80%	1	2	1	35	0.50 [0.01, 0.99]	0.95 [0.82, 0.99]
Motoyama et al.(2011),DCS,TES/AR50%	1	0	0	43	1.00 [0.03, 1.00]	1.00 [0.92, 1.00]
Nakagawa et al.(2020),TES/AR50%	3	0	7	154	0.30 [0.07, 0.65]	1.00 [0.98, 1.00]
Pastor et al.(2013),DCS,TES/AR50%,LAT10%	0	0	0	30	Not estimable	1.00 [0.88, 1.00]
Pineiro et al.(2015),TES/AR50%	0	1	0	7	Not estimable	0.88 [0.47, 1.00]
Plans et al.(2017),DCS/AR50%	0	9	4	79	0.00 [0.00, 0.60]	0.90 [0.81, 0.95]
Plans et al.(2017),DCS/TH5mA	4	8	0	80	1.00 [0.40, 1.00]	0.91 [0.83, 0.96]
Plans et al.(2017)/MT5mA	2	28	1	61	0.67 [0.09, 0.99]	0.69 [0.58, 0.78]
Plans et al.(2017)/MT5mA +AR50%	0	7	4	81	0.00 [0.00, 0.60]	0.92 [0.84, 0.97]
Plans et al.(2017)/MT5mA+ TH5mA	2	0	2	88	0.50 [0.07, 0.93]	1.00 [0.96, 1.00]
Seidel et al.(2013),DCS/TH4mA	2	10	17	71	0.11 [0.01, 0.33]	0.88 [0.78, 0.94]
Seidel et al.(2013)/MT3mA	4	21	15	58	0.21 [0.06, 0.46]	0.73 [0.62, 0.83]
Seidel et al.(2013)/MT3mA+ TH4mA	1	4	18	75	0.05 [0.00, 0.26]	0.95 [0.88, 0.99]
Seidel et al.(2020)/MT3mA	13	45	20	104	0.39 [0.23, 0.58]	0.70 [0.62, 0.77]
Song et al.(2015),TES/AR50%	1	0	2	8	0.33 [0.01, 0.91]	1.00 [0.63, 1.00]
Staarman et al.(2017),TES/AR50%	1	0	2	130	0.33 [0.01, 0.91]	1.00 [0.97, 1.00]
Szelenyi et al.(2010),TES/AR50%,TH20mA,TH100V	6	9	2	11	0.75 [0.35, 0.97]	0.55 [0.32, 0.77]
Yue et al.(2014),TES/AR50%	0	1	1	41	0.00 [0.00, 0.97]	0.98 [0.87, 1.00]

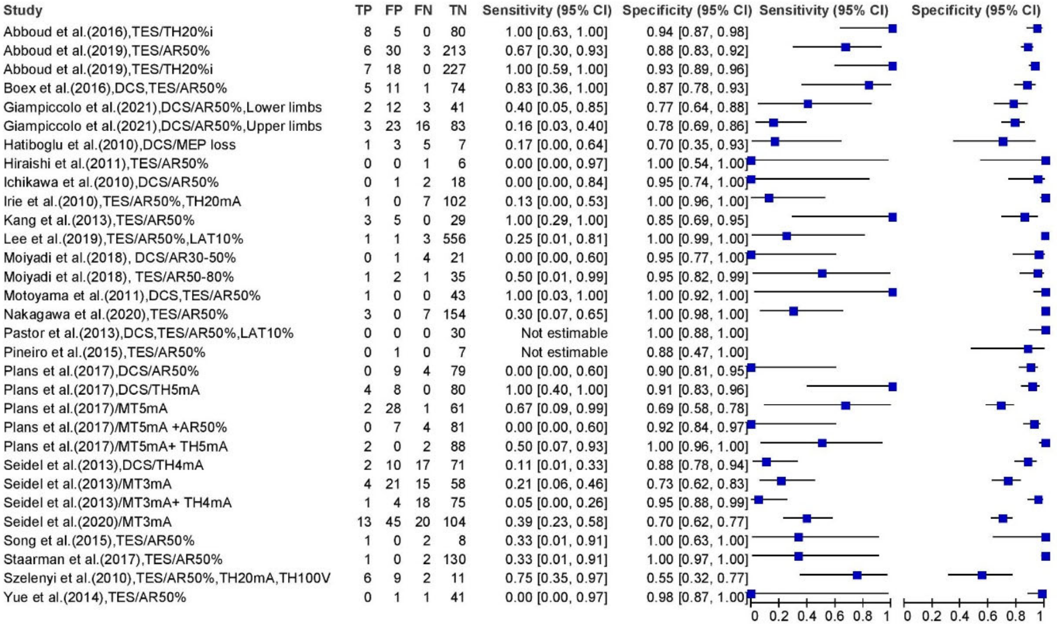

Abbreviations for Supplementary Figures S1,S2 and S3
**AR:** Amplitude criterion; **DCS:** Direct Cortical Stimulation; **FN:** False Negative; **FP:** False Positive; **i**:ipsilateral ; **LAT:** Latency criterion; **MEP:** Motor Evoked Potential ; **MT:** Motor Threshold/Mapping criterion; **PW:** Pulse Width Increase; **TES:** Transcranial Electrical Stimulation; **TH:** Threshold criterion; **TN:** True Negative; **TP:** True Positive

Supplementary Figure S4: Heatmap depicting the diagnostic accuracy measures of MEPs for permanent motor deficits

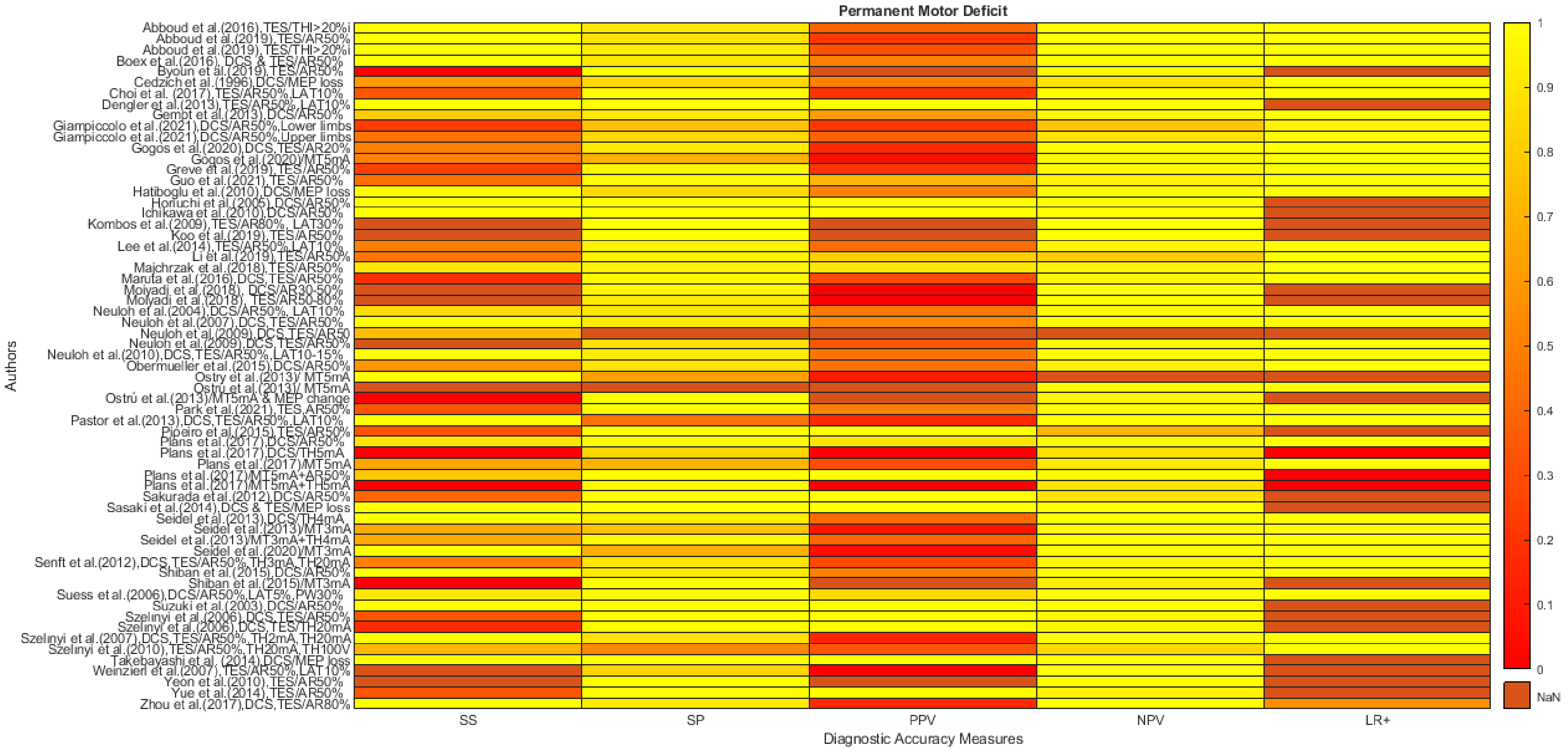

Supplementary Figure S5: Heatmap depicting the diagnostic accuracy measures of MEPs for transient motor deficits

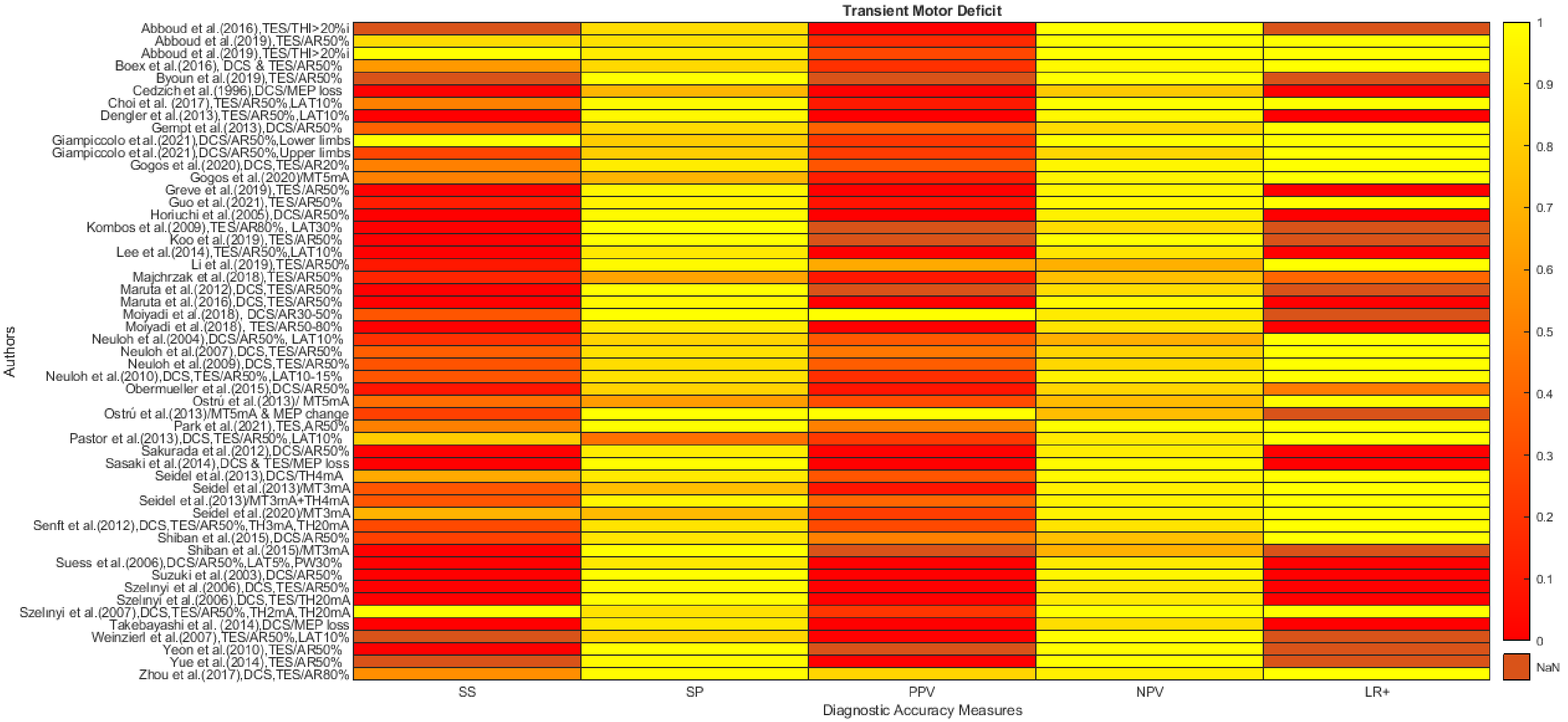

Supplementary Figure S6: Heatmap depicting the diagnostic accuracy measures of MEPs for early-transient motor deficits

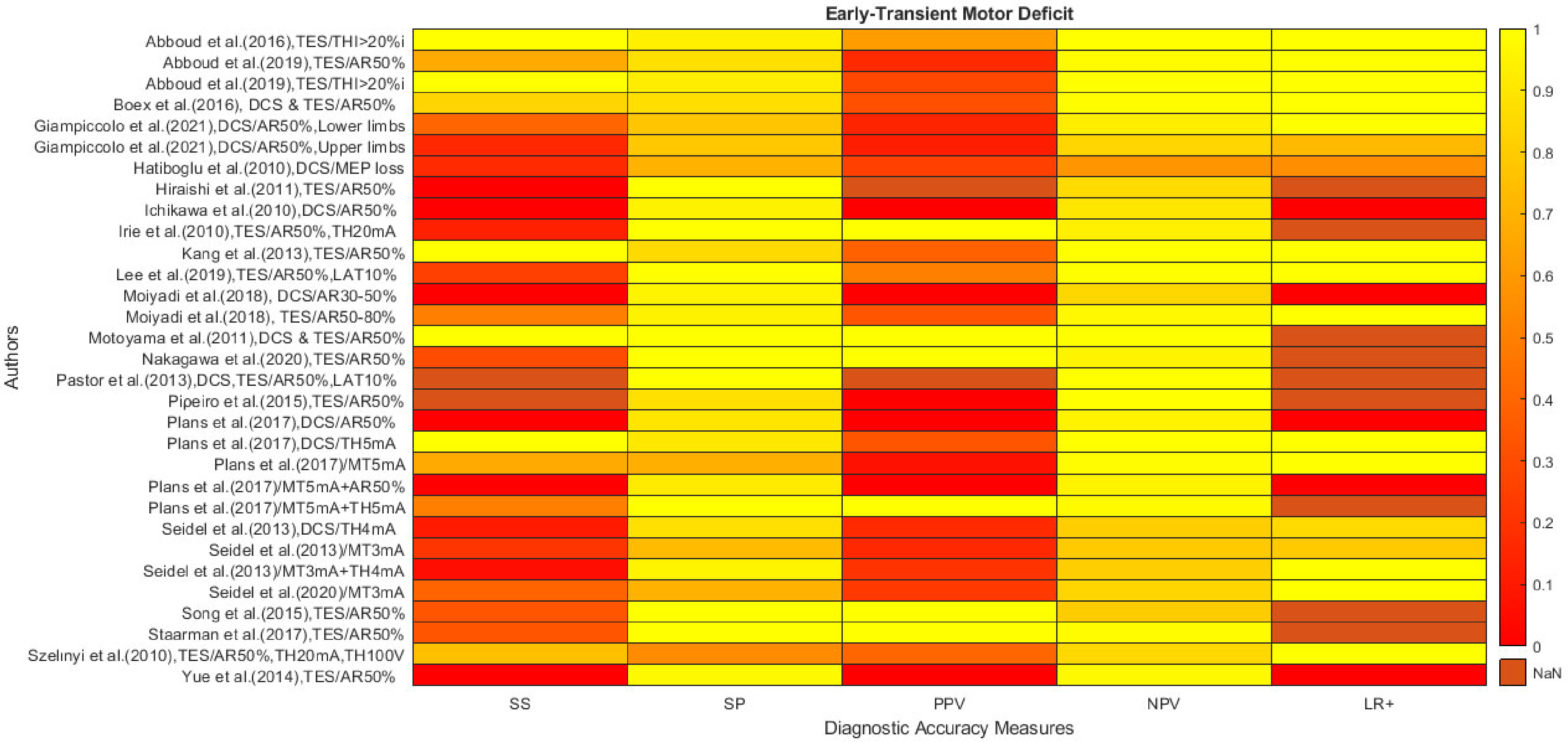

Supplementary Figure S7: Heatmap depicting the diagnostic accuracy measures of MEPs for all motor deficits

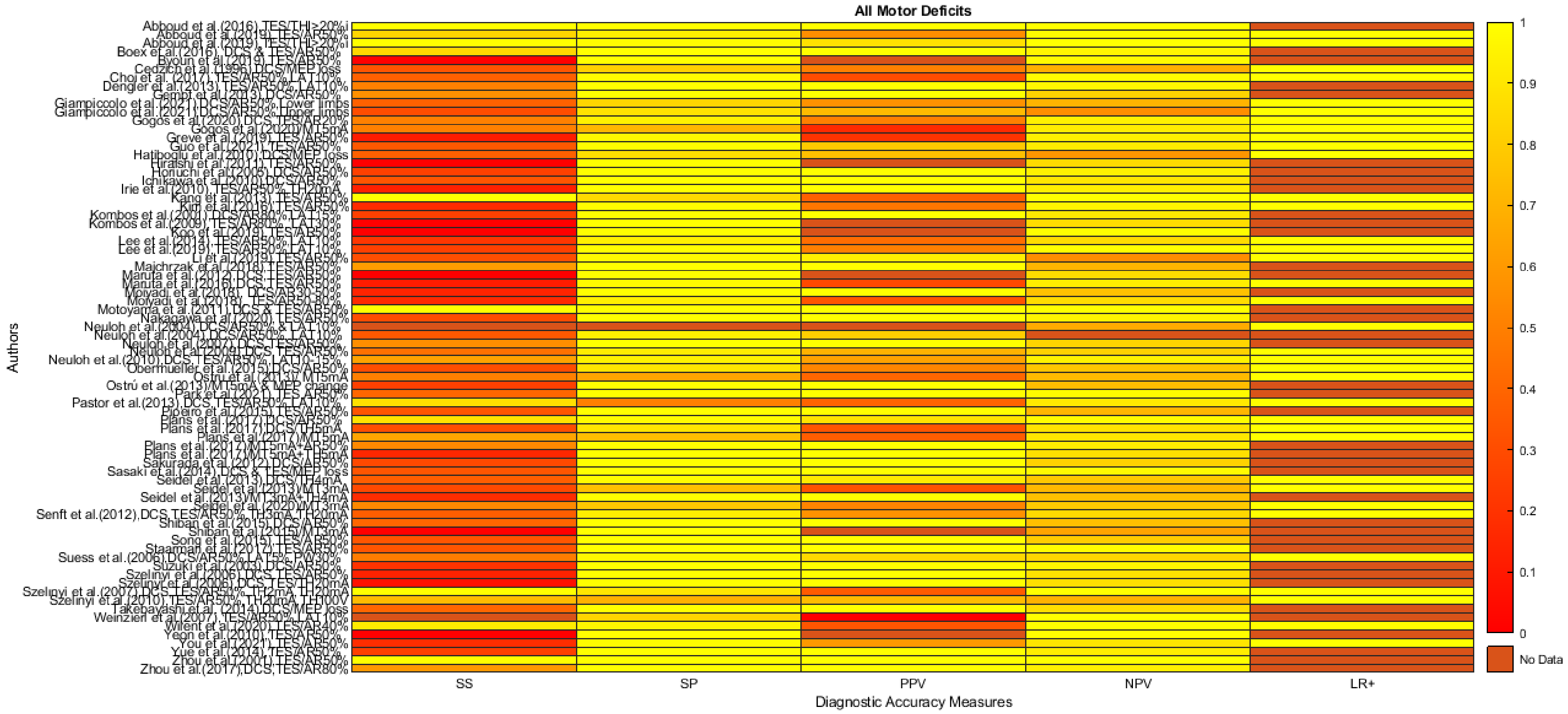

**Abbreviations for Supplementary Figures S4,S5,S6 and S7**
**AR:** Amplitude criterion; **DCS:** Direct Cortical Stimulation; **i:**ipsilateral**;** **LAT:** Latency criterion; **LR+:**Positive Likelihood Ratio; **MEP:** Motor Evoked Potential **; MT:** Motor Threshold/Mapping criterion; **NaN:** Not available ; **NPV:** Negative Predictive Value; **PPV:** Positive Predictive Value **; PW:** Pulse Width; **SS:** Sensitivity**; SP**: Specificity ; **TES:** Transcranial Electrical Stimulation; **TH:** Threshold criterion

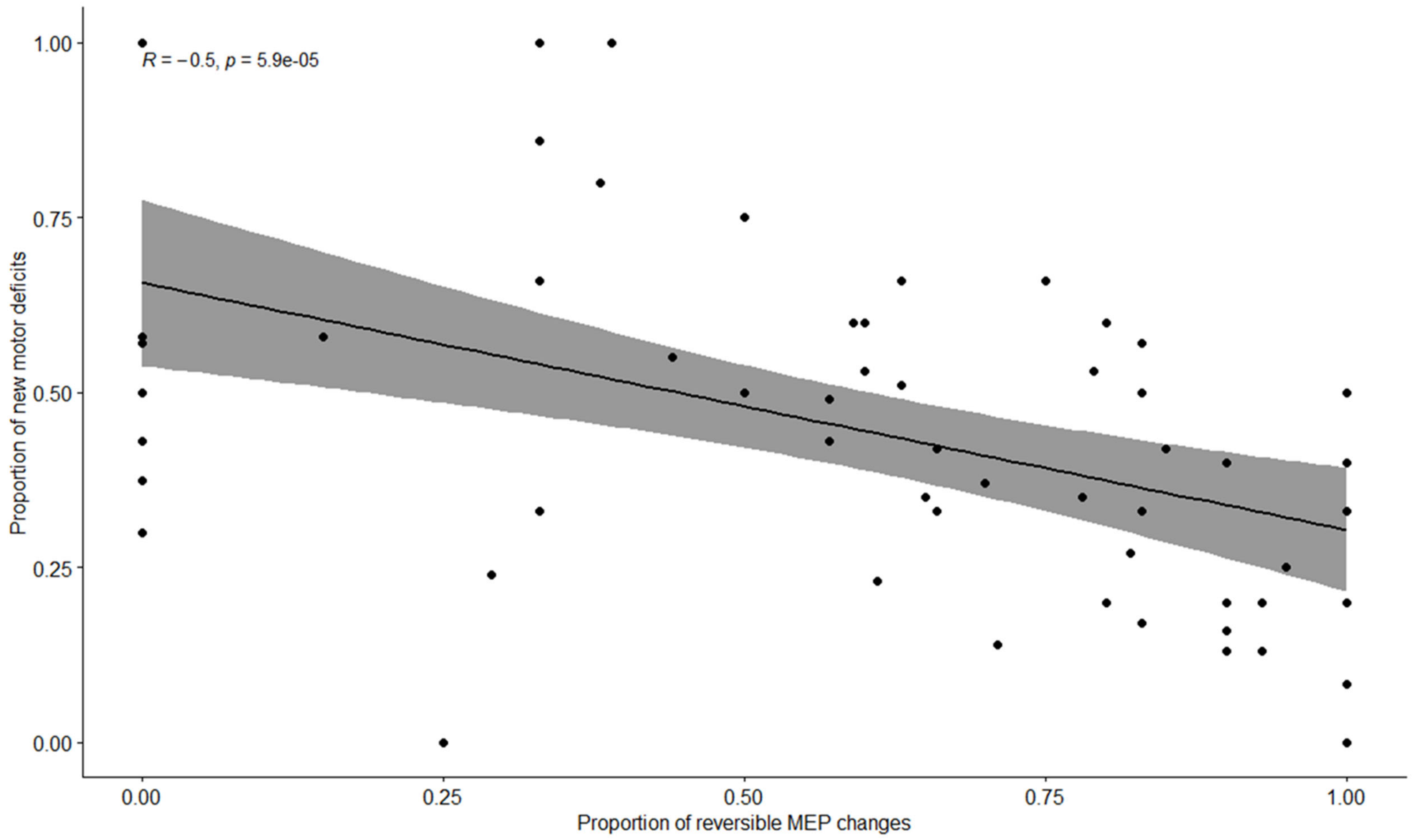

Supplementary Figure S8: Scatterplot with regression line and 95% confidence intervals depicting the negative correlation between the proportion of reversible MEP changes and the proportion of new motor deficits associated with MEP changes. Each dot represents one study. Spearman's rank correlation coefficient= -0.498($p < 0.001$).

Supplementary Table S3: Diagnostic accuracy measures for all postoperative motor deficits

All motor deficits		
<u>Authors</u>	<u>DAM</u>	<u>Value</u>
**Abboud et al.(2016),TES/THI>20%i**	SS	1
	SP	1
	PPV	1
	NPV	1
	LR+	N/A
**Abboud et al.(2019),TES/AR50%**	SS	0.8333
	SP	0.9298
	PPV	0.5556
	NPV	0.9815
	LR+	11.875
**Abboud et al.(2019),TES/THI>20%i**	SS	1
	SP	0.9870
	PPV	0.8800
	NPV	1
	LR+	76.666
**Boex et al.(2016), DCS & TES/AR50%**	SS	0.8421
	SP	1
	PPV	1
	NPV	0.9600
	LR+	N/A
**Byoun et al.(2019),TES/AR50%**	SS	0
	SP	1
	PPV	N/A
	NPV	0.9739
	LR+	N/A
**Cedzich et al.(1996),DCS/MEP loss**	SS	0.3333

	SP	0.8125
	PPV	0.5000
	NPV	0.6842
	LR+	1.777
**Choi et al. (2017),TES/AR50%,LAT10%**	SS	0.3750
	SP	0.9815
	PPV	0.3000
	NPV	0.9867
	LR+	20.250
**Dengler et al.(2013),TES/AR50%,LAT10%**	SS	0.5000
	SP	1
	PPV	1
	NPV	0.9697
	LR+	N/A
**Gempt et al.(2013),DCS/AR50%**	SS	0.5652
	SP	1
	PPV	1
	NPV	0.8246
	LR+	N/A
**Giampiccolo et al.(2021),DCS/AR50%,Upper limbs**	SS	0.3016
	SP	0.8871
	PPV	0.7308
	NPV	0.5556
	LR+	2.671
**Giampiccolo et al.(2021),DCS/AR50%,Lower limbs**	SS	0.3810
	SP	0.8378
	PPV	0.5714
	NPV	0.7045
	LR+	2.349
**Gogos et al.(2020),DCS,TES/AR20%**	SS	0.5000
	SP	0.9434

	PPV	0.5000
	NPV	0.9434
	LR+	8.833
**Gogos et al.(2020)/MT5mA**	SS	0.5000
	SP	0.7170
	PPV	0.1667
	NPV	0.9268
	LR+	1.766
**Greve et al.(2019),TES/AR50%**	SS	0.1111
	SP	0.9690
	PPV	0.2000
	NPV	0.9398
	LR+	3.583
**Guo et al.(2021),TES/AR50%**	SS	0.3438
	SP	0.9881
	PPV	0.7857
	NPV	0.9225
	LR+	28.989
**Hatiboglu et al.(2010),DCS/MEP loss**	SS	0.3750
	SP	0.8750
	PPV	0.7500
	NPV	0.5833
	LR+	3.000
**Hiraishi et al.(2011),TES/AR50%**	SS	0
	SP	1
	PPV	N/A
	NPV	0.8571
	LR+	N/A
**Horiuchi et al.(2005),DCS/AR50%**	SS	0.2500
	SP	1
	PPV	1

	NPV	0.9423
	LR+	N/A
**Ichikawa et al.(2010),DCS/AR50%**	SS	0.3333
	SP	1
	PPV	1
	NPV	0.9000
	LR+	N/A
**Irie et al.(2010),TES/AR50%,TH20mA**	SS	0.1250
	SP	1
	PPV	1
	NPV	0.9358
	LR+	N/A
**Kang et al.(2013),TES/AR50%**	SS	1
	SP	0.8529
	PPV	0.3750
	NPV	1
	LR+	6.800
**Kim et al.(2016),TES/AR50%**	SS	0.1667
	SP	0.9892
	PPV	0.4615
	NPV	0.9554
	LR+	15.452
**Kombos et al.(2001),DCS/AR80%,LAT15%**	SS	0.2500
	SP	1
	PPV	1
	NPV	0.9118
	LR+	N/A
**Kombos et al.(2009),TES/AR80%, LAT30%**	SS	0
	SP	1
	PPV	N/A
	NPV	0.8667

	LR+	N/A
**Koo et al.(2019),TES/AR50%**	SS	0
	SP	1
	PPV	N/A
	NPV	0.9928
	LR+	N/A
**Lee et al.(2014),TES/AR50%,LAT10%**	SS	0.2143
	SP	0.9429
	PPV	0.4286
	NPV	0.8571
	LR+	3.750
**Lee et al.(2019),TES/AR50%,LAT10%**	SS	0.2500
	SP	0.9982
	PPV	0.5000
	NPV	0.9946
	LR+	139.250
**Li et al.(2019),TES/AR50%**	SS	0.3061
	SP	0.9767
	PPV	0.9375
	NPV	0.5526
	LR+	13.163
**Majchrzak et al.(2018),TES/AR50%**	SS	0.6111
	SP	1
	PPV	1
	NPV	0.7083
	LR+	N/A
**Maruta et al.(2012),DCS,TES/AR50%**	SS	0
	SP	1
	PPV	N/A
	NPV	0.8636
	LR+	N/A

**Maruta et al.(2016),DCS,TES/AR50%**	SS	0.1111
	SP	0.9778
	PPV	0.2857
	NPV	0.9322
	LR+	5.000
**Moiyadi et al.(2018), DCS/AR30-50%**	SS	0.1429
	SP	1
	PPV	1
	NPV	0.7600
	LR+	N/A
**Moiyadi et al.(2018), TES/AR50-80%**	SS	0.1667
	SP	0.9394
	PPV	0.3333
	NPV	0.8611
	LR+	2.750
**Motoyama et al.(2011),DCS & TES/AR50%**	SS	1
	SP	1
	PPV	1
	NPV	1
	LR+	N/A
**Nakagawa et al.(2020),TES/AR50%**	SS	0.3000
	SP	1
	PPV	1
	NPV	0.9565
	LR+	N/A
**Neuloh et al.(2004),DCS/AR50%, LAT10%**	SS	0.3443
	SP	0.9419
	PPV	0.8077
	NPV	0.6694
	LR+	5.921
**Neuloh et al.(2007),DCS,TES/AR50%**	SS	0.5556

	SP	1
	PPV	1
	NPV	0.8356
	LR+	N/A
**Neuloh et al.(2009),DCS,TES/AR50%**	SS	0.4423
	SP	0.9281
	PPV	0.6970
	NPV	0.8165
	LR+	6.148
**Neuloh et al.(2010),DCS,TES/AR50%,LAT10-15%**	SS	0.6364
	SP	0.9467
	PPV	0.6364
	NPV	0.9467
	LR+	11.931
**Obermueller et al.(2015),DCS/AR50%**	SS	0.3023
	SP	0.8957
	PPV	0.5200
	NPV	0.7744
	LR+	2.897
**Ostrý et al.(2013)/ MT5mA**	SS	0.5000
	SP	0.6471
	PPV	0.4000
	NPV	0.7333
	LR+	1.416
**Ostrý et al.(2013)/MT5mA & MEP change**	SS	0.2500
	SP	1
	PPV	1
	NPV	0.7391
	LR+	N/A
**Park et al.(2021),TES,AR50%**	SS	0.4000
	SP	1

	PPV	1
	NPV	0.9810
	LR+	N/A
**Pastor et al.(2013),DCS,TES/AR50%,LAT10%**	SS	0.8750
	SP	0.5000
	PPV	0.3889
	NPV	0.9167
	LR+	1.750
**Piñeiro et al.(2015),TES/AR50%**	SS	0.3333
	SP	1
	PPV	1
	NPV	0.7143
	LR+	N/A
**Plans et al.(2017),DCS/AR50%**	SS	0.8889
	SP	0.9880
	PPV	0.8889
	NPV	0.9880
	LR+	73.777
**Plans et al.(2017),DCS/TH5mA**	SS	0.3077
	SP	0.8987
	PPV	0.3333
	NPV	0.8875
	LR+	3.038
**Plans et al.(2017)/MT5mA**	SS	0.6471
	SP	0.7467
	PPV	0.3667
	NPV	0.9032
	LR+	2.554
**Plans et al.(2017)/MT5mA+AR50%**	SS	0.5385
	SP	1
	PPV	1

	NPV	0.9294
	LR+	N/A
**Plans et al.(2017)/MT5mA+TH5mA**	SS	0.1538
	SP	1
	PPV	1
	NPV	0.8778
	LR+	N/A
**Sakurada et al.(2012),DCS/AR50%**	SS	0.2857
	SP	1
	PPV	1
	NPV	0.8214
	LR+	N/A
**Sasaki et al.(2014),DCS & TES/MEP loss**	SS	0.3333
	SP	1
	PPV	1
	NPV	0.9771
	LR+	N/A
**Seidel et al.(2013),DCS/TH4mA**	SS	0.3667
	SP	0.9857
	PPV	0.9167
	NPV	0.7841
	LR+	25.666
**Seidel et al.(2013)/MT3mA**	SS	0.2857
	SP	0.7571
	PPV	0.3200
	NPV	0.7260
	LR+	1.176
**Seidel et al.(2013)/MT3mA+TH4mA**	SS	0.1786
	SP	1
	PPV	1
	NPV	0.7527

	LR+	N/A
**Seidel et al.(2020)/MT3mA**	SS	0.5357
	SP	0.7778
	PPV	0.5172
	NPV	0.7903
	LR+	2.410
**Senft et al.(2012),DCS,TES/AR50%,TH3mA,TH20mA**	SS	0.3636
	SP	0.9302
	PPV	0.5714
	NPV	0.8511
	LR+	5.212
**Shiban et al.(2015),DCS/AR50%**	SS	0.4000
	SP	1
	PPV	1
	NPV	0.7500
	LR+	N/A
**Shiban et al.(2015)/MT3mA**	SS	0
	SP	1
	PPV	N/A
	NPV	0.6429
	LR+	N/A
**Song et al.(2015),TES/AR50%**	SS	0.3333
	SP	1
	PPV	1
	NPV	0.8000
	LR+	N/A
**Staarman et al.(2017),TES/AR50%**	SS	0.3333
	SP	1
	PPV	1
	NPV	0.9848
	LR+	N/A

**Suess et al.(2006),DCS/AR50%,LAT5%,PW30%**	SS	0.4857
	SP	0.9691
	PPV	0.8500
	NPV	0.8393
	LR+	15.704
**Suzuki et al.(2003),DCS/AR50%**	SS	0.2000
	SP	1
	PPV	1
	NPV	0.9626
	LR+	N/A
**Szelényi et al.(2006),DCS,TES/AR50%**	SS	0.1333
	SP	1
	PPV	1
	NPV	0.8839
	LR+	N/A
**Szelényi et al.(2006),DCS,TES/TH20mA**	SS	0.0667
	SP	1
	PPV	1
	NPV	0.8761
	LR+	N/A
**Szelényi et al.(2007),DCS,TES/AR50%,TH2mA,TH20mA**	SS	1
	SP	0.9043
	PPV	0.3571
	NPV	1
	LR+	10.444
**Szelényi et al.(2010),TES/AR50%,TH20mA,TH100V**	SS	0.7333
	SP	0.6923
	PPV	0.7333
	NPV	0.6923
	LR+	2.383

**Takebayashi et al. (2014),DCS/MEP loss**	SS	0.4000
	SP	1
	PPV	1
	NPV	0.8696
	LR+	N/A
**Weinzierl et al.(2007),TES/AR50%,LAT10%**	SS	N/A
	SP	0.8333
	PPV	0
	NPV	1
	LR+	N/A
**Wilent et al.(2020),TES/AR40%**	SS	0.9231
	SP	0.9680
	PPV	0.3333
	NPV	0.9986
	LR+	28.846
**Yeon et al.(2010),TES/AR50%**	SS	0
	SP	1
	PPV	N/A
	NPV	0.9898
	LR+	N/A
**You et al.(2021),TES/AR50%**	SS	0.1765
	SP	0.9835
	PPV	0.6000
	NPV	0.8947
	LR+	10.676
**Yue et al.(2014),TES/AR50%**	SS	0.2500
	SP	1
	PPV	1
	NPV	0.9286
	LR+	N/A
**Zhou et al.(2001),TES/AR50%**	SS	1

	SP	1
	PPV	1
	NPV	1
	LR+	N/A
**Zhou et al.(2017),DCS,TES/AR80%**	SS	0.6000
	SP	1
	PPV	1
	NPV	0.9375
	LR+	N/A

Supplementary Table S4: Diagnostic accuracy measures for permanent postoperative motor deficits

**Permanent motor deficit**		
**<u>Authors</u>**	<u>DAM</u>	<u>Value</u>
**Abboud et al.(2016),TES/THI>20%i**	SS	1
	SP	0.9091
	PPV	0.3846
	NPV	1
	LR+	11
**Abboud et al.(2019),TES/AR50%**	SS	1
	SP	0.8852
	PPV	0.2222
	NPV	1
	LR+	8.714
**Abboud et al.(2019),TES/THI>20%i**	SS	1
	SP	0.9303
	PPV	0.3200
	NPV	1
	LR+	14.352
**Boex et al.(2016), DCS & TES/AR50%**	SS	1
	SP	0.9036

	PPV	0.5000
	NPV	1
	LR+	10.375
**Byoun et al.(2019),TES/AR50%**	SS	0
	SP	1
	PPV	N/A
	NPV	0.9739
	LR+	N/A
**Cedzich et al.(1996),DCS/MEP loss**	SS	0.6000
	SP	0.8500
	PPV	0.5000
	NPV	0.8947
	LR+	4.000
**Choi et al. (2017),TES/AR50%,LAT10%**	SS	0.3333
	SP	0.9789
	PPV	0.2000
	NPV	0.9894
	LR+	15.833
**Dengler et al.(2013),TES/AR50%,LAT10%**	SS	1
	SP	1
	PPV	1
	NPV	1
	LR+	N/A
**Gempt et al.(2013),DCS/AR50%**	SS	0.8000
	SP	0.9167
	PPV	0.6154
	NPV	0.9649
	LR+	9.600
**Giampiccolo et al.(2021),DCS/AR50%,Upper limbs**	SS	0.4545
	SP	0.8447
	PPV	0.3846

	NPV	0.8788
	LR+	2.926
**Giampiccolo et al.(2021),DCS/AR50%,Lower limbs**	SS	0.2308
	SP	0.7556
	PPV	0.2143
	NPV	0.7727
	LR+	0.9441
**Gogos et al.(2020),DCS,TES/AR20%**	SS	0.5000
	SP	0.9123
	PPV	0.1667
	NPV	0.9811
	LR+	5.700
**Gogos et al.(2020)/MT5mA**	SS	0.5000
	SP	0.7018
	PPV	0.0556
	NPV	0.9756
	LR+	1.676
**Greve et al.(2019),TES/AR50%**	SS	0.2500
	SP	0.9701
	PPV	0.2000
	NPV	0.9774
	LR+	8.375
**Guo et al.(2021),TES/AR50%**	SS	0.4348
	SP	0.9847
	PPV	0.7143
	NPV	0.9520
	LR+	28.478
**Hatiboglu et al.(2010),DCS/MEP loss**	SS	1
	SP	0.8571
	PPV	0.5000
	NPV	1

	LR+	7.000
**Horiuchi et al.(2005),DCS/AR50%**	SS	1
	SP	1
	PPV	1
	NPV	1
	LR+	N/A
**Ichikawa et al.(2010),DCS/AR50%**	SS	1
	SP	1
	PPV	1
	NPV	1
	LR+	N/A
**Kombos et al.(2009),TES/AR80%, LAT30%**	SS	N/A
	SP	1
	PPV	N/A
	NPV	1
	LR+	N/A
**Koo et al.(2019),TES/AR50%**	SS	N/A
	SP	1
	PPV	N/A
	NPV	1
	LR+	N/A
**Lee et al.(2014),TES/AR50%,LAT10%**	SS	0.5000
	SP	0.9487
	PPV	0.4286
	NPV	0.9610
	LR+	9.750
**Li et al.(2019),TES/AR50%**	SS	0.4643
	SP	0.9531
	PPV	0.8125
	NPV	0.8026
	LR+	9.904

**Majchrzak et al.(2018),TES/AR50%**	SS	0.9091
	SP	0.9583
	PPV	0.9091
	NPV	0.9583
	LR+	21.818
**Maruta et al.(2016),DCS,TES/AR50%**	SS	0.1818
	SP	0.9784
	PPV	0.2857
	NPV	0.9619
	LR+	8.436
**Moiyadi et al.(2018), DCS/AR30-50%**	SS	N/A
	SP	0.9615
	PPV	0
	NPV	1
	LR+	N/A
**Moiyadi et al.(2018), TES/AR50-80%**	SS	N/A
	SP	0.9231
	PPV	0
	NPV	1
	LR+	N/A
**Neuloh et al.(2004),DCS/AR50%, LAT10%**	SS	0.8571
	SP	0.8947
	PPV	0.4615
	NPV	0.9835
	LR+	8.142
**Neuloh et al.(2007),DCS,TES/AR50%**	SS	1
	SP	0.9125
	PPV	0.5333
	NPV	1
	LR+	11.428
**Neuloh et al.(2009),DCS,TES/AR50**	SS	0.7333

	SP	0.8750
	PPV	0.3333
	NPV	0.9747
	LR+	5.866
**Neuloh et al.(2010),DCS,TES/AR50%,LAT10-15%**	SS	1
	SP	0.9259
	PPV	0.4545
	NPV	1
	LR+	13.500
**Obermueller et al.(2015),DCS/AR50%**	SS	0.5789
	SP	0.8993
	PPV	0.4400
	NPV	0.9398
	LR+	5.748
**Ostry et al.(2013)/ MT5mA**	SS	1
	SP	0.6250
	PPV	0.1000
	NPV	1
	LR+	2.666
**Ostrý et al.(2013)/MT5mA & MEP change**	SS	0
	SP	1
	PPV	N/A
	NPV	0.9600
	LR+	N/A
**Park et al.(2021),TES,AR50%**	SS	0.3333
	SP	0.9936
	PPV	0.5000
	NPV	0.9873
	LR+	52.166
**Pastor et al.(2013),DCS,TES/AR50%,LAT10%**	SS	1
	SP	0.4444

	PPV	0.1667
	NPV	1
	LR+	1.800
**Piñeiro et al.(2015),TES/AR50%**	SS	0.3333
	SP	1
	PPV	1
	NPV	0.7143
	LR+	N/A
**Plans et al.(2017),DCS/AR50%**	SS	0.8889
	SP	0.9868
	PPV	0.8889
	NPV	0.9868
	LR+	67.555
**Plans et al.(2017),DCS/TH5mA**	SS	0
	SP	0.8421
	PPV	0
	NPV	0.8767
	LR+	0
**Plans et al.(2017)/MT5mA**	SS	0.6429
	SP	0.7042
	PPV	0.3000
	NPV	0.9091
	LR+	2.173
**Plans et al.(2017)/MT5mA+AR50%**	SS	0.7778
	SP	1
	PPV	1
	NPV	0.9744
	LR+	0
**Plans et al.(2017)/MT5mA+TH5mA**	SS	0
	SP	0.9737
	PPV	0

	NPV	0.8916
	LR+	0
**Sakurada et al.(2012),DCS/AR50%**	SS	0.4000
	SP	1
	PPV	1
	NPV	0.8929
	LR+	N/A
**Sasaki et al.(2014),DCS & TES/MEP loss**	SS	1
	SP	1
	PPV	1
	NPV	1
	LR+	N/A
**Seidel et al.(2013),DCS/TH4mA**	SS	1
	SP	0.9263
	PPV	0.4167
	NPV	1
	LR+	13.571
**Seidel et al.(2013)/MT3mA**	SS	0.6667
	SP	0.7579
	PPV	0.0800
	NPV	0.9863
	LR+	2.753
**Seidel et al.(2013)/MT3mA+TH4mA**	SS	0.6667
	SP	0.9684
	PPV	0.4000
	NPV	0.9892
	LR+	21.111
**Seidel et al.(2020)/MT3mA**	SS	1
	SP	0.6927
	PPV	0.0517
	NPV	1

	LR+	3.200
**Senft et al.(2012),DCS,TES/AR50%,TH3mA,TH20mA**	SS	0.5000
	SP	0.9000
	PPV	0.2857
	NPV	0.9574
	LR+	5.000
**Shiban et al.(2015),DCS/AR50%**	SS	1
	SP	0.9231
	PPV	0.5000
	NPV	1
	LR+	13.000
**Shiban et al.(2015)/MT3mA**	SS	0
	SP	1
	PPV	N/A
	NPV	0.9286
	LR+	N/A
**Suess et al.(2006),DCS/AR50%,LAT5%,PW30%**	SS	0.8947
	SP	0.9859
	PPV	0.8500
	NPV	0.9906
	LR+	63.526
**Suzuki et al.(2003),DCS/AR50%**	SS	1
	SP	1
	PPV	1
	NPV	1
	LR+	N/A
**Szelényi et al.(2006),DCS,TES/AR50%**	SS	0.3333
	SP	1
	PPV	1
	NPV	0.9643
	LR+	N/A

**Szelényi et al.(2006),DCS,TES/TH20mA**	SS	0.1667
	SP	1
	PPV	1
	NPV	0.9558
	LR+	N/A
**Szelényi et al.(2007),DCS,TES/AR50%,TH2mA,TH20mA**	SS	1
	SP	0.8763
	PPV	0.1429
	NPV	1
	LR+	8.083
**Szelényi et al.(2010),TES/AR50%,TH20mA,TH100V**	SS	0.7143
	SP	0.5238
	PPV	0.3333
	NPV	0.8462
	LR+	1.500
**Takebayashi et al. (2014),DCS/MEP loss**	SS	1
	SP	1
	PPV	1
	NPV	1
	LR+	N/A
**Weinzierl et al.(2007),TES/AR50%,LAT10%**	SS	N/A
	SP	0.8333
	PPV	0
	NPV	1
	LR+	N/A
**Yeon et al.(2010),TES/AR50%**	SS	N/A
	SP	1
	PPV	N/A
	NPV	1
	LR+	N/A

**Yue et al.(2014),TES/AR50%**	SS	0.3333
	SP	1
	PPV	1
	NPV	0.9524
	LR+	N/A
**Zhou et al.(2017),DCS,TES/AR80%**	SS	1
	SP	0.9275
	PPV	0.1667
	NPV	1
	LR+	0.5556

Supplementary Table S5: Diagnostic accuracy measures for transient postoperative motor deficits

Authors	DAM	Value
**Abboud et al.(2016),TES/THI>20%i**	SS	N/A
	SP	0.8602
	PPV	0
	NPV	1
	LR+	N/A
**Abboud et al.(2019),TES/AR50%**	SS	0.8571
	SP	0.8776
	PPV	0.1667
	NPV	0.9954
	LR+	7.000
**Abboud et al.(2019),TES/THI>20%i**	SS	1
	SP	0.9265
	PPV	0.2800

	NPV	1
	LR+	13.611
**Boex et al.(2016), DCS & TES/AR50%**	SS	0.6000
	SP	0.8488
	PPV	0.1875
	NPV	0.9733
	LR+	3.969
**Byoun et al.(2019),TES/AR50%**	SS	N/A
	SP	1
	PPV	N/A
	NPV	1
	LR+	N/A
**Cedzich et al.(1996),DCS/MEP loss**	SS	0
	SP	0.7143
	PPV	0
	NPV	0.7895
	LR+	0
**Choi et al. (2017),TES/AR50%,LAT10%**	SS	0.5000
	SP	0.9766
	PPV	0.1000
	NPV	0.9973
	LR+	21.333
**Dengler et al.(2013),TES/AR50%,LAT10%**	SS	0
	SP	0.9697
	PPV	0
	NPV	0.9697
	LR+	0
**Gempt et al.(2013),DCS/AR50%**	SS	0.3846
	SP	0.8596
	PPV	0.3846
	NPV	0.8596

	LR+	2.740
**Giampiccolo et al.(2021),DCS/AR50%,Upper limbs**	SS	0.2727
	SP	0.8058
	PPV	0.2308
	NPV	0.8384
	LR+	1.404
**Giampiccolo et al.(2021),DCS/AR50%,Lower limbs**	SS	1
	SP	0.8000
	PPV	0.2143
	NPV	1
	LR+	5.000
**Gogos et al.(2020),DCS,TES/AR20%**	SS	0.5000
	SP	0.9273
	PPV	0.3333
	NPV	0.9623
	LR+	6.875
**Gogos et al.(2020)/MT5mA**	SS	0.5000
	SP	0.7091
	PPV	0.1111
	NPV	0.9512
	LR+	1.718
**Greve et al.(2019),TES/AR50%**	SS	0
	SP	0.9624
	PPV	0
	NPV	0.9624
	LR+	0
**Guo et al.(2021),TES/AR50%**	SS	0.1111
	SP	0.9529
	PPV	0.0714
	NPV	0.9705
	LR+	2.359

**Horiuchi et al.(2005),DCS/AR50%**	SS	0
	SP	0.9800
	PPV	0
	NPV	0.9423
	LR+	0
**Kombos et al.(2009),TES/AR80%, LAT30%**	SS	0
	SP	1
	PPV	N/A
	NPV	0.8667
	LR+	N/A
**Koo et al.(2019),TES/AR50%**	SS	0
	SP	1
	PPV	N/A
	NPV	0.9928
	LR+	N/A
**Lee et al.(2014),TES/AR50%,LAT10%**	SS	0
	SP	0.9079
	PPV	0
	NPV	0.8961
	LR+	0
**Li et al.(2019),TES/AR50%**	SS	0.0952
	SP	0.9767
	PPV	0.6667
	NPV	0.6885
	LR+	4.095
**Majchrzak et al.(2018),TES/AR50%**	SS	0.1429
	SP	0.6429
	PPV	0.0909
	NPV	0.7500
	LR+	0.400
**Maruta et al.(2012),DCS,TES/AR50%**	SS	0

	SP	1
	PPV	N/A
	NPV	0.8636
	LR+	N/A
**Maruta et al.(2016),DCS,TES/AR50%**	SS	0
	SP	0.9703
	PPV	0
	NPV	0.9703
	LR+	0
**Moiyadi et al.(2018), DCS/AR30-50%**	SS	0.3333
	SP	1
	PPV	1
	NPV	0.9200
	LR+	N/A
**Moiyadi et al.(2018), TES/AR50-80%**	SS	0
	SP	0.9143
	PPV	0
	NPV	0.8889
	LR+	0
**Neuloh et al.(2004),DCS/AR50%, LAT10%**	SS	0.1915
	SP	0.8300
	PPV	0.3462
	NPV	0.6860
	LR+	1.126
**Neuloh et al.(2007),DCS,TES/AR50%**	SS	0.3684
	SP	0.8841
	PPV	0.4667
	NPV	0.8356
	LR+	3.177
**Neuloh et al.(2009),DCS,TES/AR50%**	SS	0.3243
	SP	0.8636

	PPV	0.3636
	NPV	0.8418
	LR+	2.378
**Neuloh et al.(2010),DCS,TES/AR50%,LAT10-15%**	SS	0.3333
	SP	0.8875
	PPV	0.1818
	NPV	0.9467
	LR+	2.963
**Obermueller et al.(2015),DCS/AR50%**	SS	0.0833
	SP	0.8284
	PPV	0.0800
	NPV	0.8346
	LR+	0.4855
**Ostrý et al.(2013)/ MT5mA**	SS	0.4286
	SP	0.6111
	PPV	0.3000
	NPV	0.7333
	LR+	1.102
**Ostrý et al.(2013)/MT5mA & MEP change**	SS	0.2500
	SP	1
	PPV	1
	NPV	0.7391
	LR+	N/A
**Park et al.(2021),TES,AR50%**	SS	0.5000
	SP	0.9937
	PPV	0.5000
	NPV	0.9937
	LR+	78.750
**Pastor et al.(2013),DCS,TES/AR50%,LAT10%**	SS	0.8000
	SP	0.4400
	PPV	0.2222

	NPV	0.9167
	LR+	1.428
**Sakurada et al.(2012),DCS/AR50%**	SS	0
	SP	0.9286
	PPV	0
	NPV	0.9286
	LR+	0
**Sasaki et al.(2014),DCS & TES/MEP loss**	SS	0
	SP	0.9884
	PPV	0
	NPV	0.9771
	LR+	0
**Seidel et al.(2013),DCS/TH4mA**	SS	0.6667
	SP	0.9149
	PPV	0.3333
	NPV	0.9773
	LR+	7.833
**Seidel et al.(2013)/MT3mA**	SS	0.3333
	SP	0.7500
	PPV	0.0800
	NPV	0.9452
	LR+	1.333
**Seidel et al.(2013)/MT3mA+TH4mA**	SS	0.3333
	SP	0.9674
	PPV	0.4000
	NPV	0.9570
	LR+	10.222
**Seidel et al.(2020)/MT3mA**	SS	0.7000
	SP	0.7284
	PPV	0.2414
	NPV	0.9516

	LR+	2.812
**Senft et al.(2012),DCS,TES/AR50%,TH3mA,TH20mA**	SS	0.2857
	SP	0.8936
	PPV	0.2857
	NPV	0.8936
	LR+	2.685
**Shiban et al.(2015),DCS/AR50%**	SS	0.2500
	SP	0.9000
	PPV	0.5000
	NPV	0.7500
	LR+	2.500
**Shiban et al.(2015)/MT3mA**	SS	0
	SP	1
	PPV	N/A
	NPV	0.6923
	LR+	N/A
**Suess et al.(2006),DCS/AR50%,LAT5%,PW30%**	SS	0
	SP	0.9074
	PPV	0
	NPV	0.9245
	LR+	0
**Suzuki et al.(2003),DCS/AR50%**	SS	0
	SP	0.9904
	PPV	0
	NPV	0.9626
	LR+	0
**Szelényi et al.(2006),DCS,TES/AR50%**	SS	0
	SP	0.9810
	PPV	0
	NPV	0.9196
	LR+	0

**Szelényi et al.(2006),DCS,TES/TH20mA**	SS	0
	SP	0.9905
	PPV	0
	NPV	0.9204
	LR+	0
**Szelényi et al.(2007),DCS,TES/AR50%,TH2mA,TH20mA**	SS	1
	SP	0.8854
	PPV	0.2143
	NPV	1
	LR+	8.727
**Takebayashi et al. (2014),DCS/MEP loss**	SS	0
	SP	0.9091
	PPV	0
	NPV	0.8696
	LR+	0
**Weinzierl et al.(2007),TES/AR50%,LAT10%**	SS	N/A
	SP	0.8333
	PPV	0
	NPV	1
	LR+	N/A
**Yeon et al.(2010),TES/AR50%**	SS	0
	SP	1
	PPV	N/A
	NPV	0.9898
	LR+	N/A
**Yue et al.(2014),TES/AR50%**	SS	N/A
	SP	0.9767
	PPV	0
	NPV	1
	LR+	N/A

**Zhou et al.(2017),DCS,TES/AR80%**	SS	0.5556
	SP	0.9836
	PPV	0.8333
	NPV	0.9375
	LR+	33.888

Supplementary Table S6: Diagnostic accuracy measures for early-transient postoperative motor deficits

**Authors**	**DAM**	**Value**
**Abboud et al.(2016),TES/THI>20%i**	SS	1
	SP	0.9412
	PPV	0.6154
	NPV	1
	LR+	17.000
**Abboud et al.(2019),TES/AR50%**	SS	0.6667
	SP	0.8765
	PPV	0.1667
	NPV	0.9861
	LR+	5.400
**Abboud et al.(2019),TES/THI>20%i**	SS	1
	SP	0.9265
	PPV	0.2800
	NPV	1
	LR+	13.611
**Boex et al.(2016), DCS & TES/AR50%**	SS	0.8333
	SP	0.8706
	PPV	0.3125

	NPV	0.9867
	LR+	6.439
**Giampiccolo et al.(2021),DCS/AR50%,Upper limbs**	SS	0.1579
	SP	0.7830
	PPV	0.1154
	NPV	0.8384
	LR+	0.727
**Giampiccolo et al.(2021),DCS/AR50%,Lower limbs**	SS	0.4000
	SP	0.7736
	PPV	0.1429
	NPV	0.9318
	LR+	1.766
**Hatiboglu et al.(2010),DCS/MEP loss**	SS	0.1667
	SP	0.7000
	PPV	0.2500
	NPV	0.5833
	LR+	0.555
**Hiraishi et al.(2011),TES/AR50%**	SS	0
	SP	1
	PPV	N/A
	NPV	0.8571
	LR+	N/A
**Ichikawa et al.(2010),DCS/AR50%**	SS	0
	SP	0.9474
	PPV	0
	NPV	0.9000
	LR+	0
**Irie et al.(2010),TES/AR50%,TH20mA**	SS	0.1250
	SP	1
	PPV	1
	NPV	0.9358

	LR+	N/A
**Kang et al.(2013),TES/AR50%**	SS	1
	SP	0.8529
	PPV	0.3750
	NPV	1
	LR+	6.800
**Lee et al.(2019),TES/AR50%,LAT10%**	SS	0.2500
	SP	0.9982
	PPV	0.5000
	NPV	0.9946
	LR+	139.250
**Moiyadi et al.(2018), DCS/AR30-50%**	SS	0
	SP	0.9545
	PPV	0
	NPV	0.8400
	LR+	0
**Moiyadi et al.(2018), TES/AR50-80%**	SS	0.5000
	SP	0.9459
	PPV	0.3333
	NPV	0.9722
	LR+	9.250
**Motoyama et al.(2011),DCS & TES/AR50%**	SS	1
	SP	1
	PPV	1
	NPV	1
	LR+	N/A
**Nakagawa et al.(2020),TES/AR50%**	SS	0.3000
	SP	1
	PPV	1
	NPV	0.9565
	LR+	N/A

**Pastor et al.(2013),DCS,TES/AR50%,LAT10%**	SS	N/A
	SP	1
	PPV	N/A
	NPV	1
	LR+	N/A
**Piñeiro et al.(2015),TES/AR50%**	SS	N/A
	SP	0.8750
	PPV	0
	NPV	1
	LR+	N/A
**Plans et al.(2017),DCS/AR50%**	SS	0
	SP	0.8977
	PPV	0
	NPV	0.9518
	LR+	0
**Plans et al.(2017),DCS/TH5mA**	SS	1
	SP	0.9091
	PPV	0.3333
	NPV	1
	LR+	11.000
**Plans et al.(2017)/MT5mA**	SS	0.6667
	SP	0.6854
	PPV	0.0667
	NPV	0.9839
	LR+	2.119
**Plans et al.(2017)/MT5mA+AR50%**	SS	0
	SP	0.9205
	PPV	0
	NPV	0.9529
	LR+	0
**Plans et al.(2017)/MT5mA+TH5mA**	SS	0.5000

	SP	1
	PPV	1
	NPV	0.9778
	LR+	N/A
**Seidel et al.(2013),DCS/TH4mA**	SS	0.1053
	SP	0.8765
	PPV	0.1667
	NPV	0.8068
	LR+	0.852
**Seidel et al.(2013)/MT3mA**	SS	0.2105
	SP	0.7342
	PPV	0.1600
	NPV	0.7945
	LR+	0.792
**Seidel et al.(2013)/MT3mA+TH4mA**	SS	0.0526
	SP	0.9494
	PPV	0.2000
	NPV	0.8065
	LR+	1.039
**Seidel et al.(2020)/MT3mA**	SS	0.3939
	SP	0.6980
	PPV	0.2241
	NPV	0.8387
	LR+	1.304
**Song et al.(2015),TES/AR50%**	SS	0.3333
	SP	1
	PPV	1
	NPV	0.8000
	LR+	N/A
**Staarman et al.(2017),TES/AR50%**	SS	0.3333
	SP	1

	PPV	1
	NPV	0.9848
	LR+	N/A
**Szelényi et al.(2010),TES/AR50%,TH20mA,TH100V**	SS	0.7500
	SP	0.5500
	PPV	0.4000
	NPV	0.8462
	LR+	1.666
**Yue et al.(2014),TES/AR50%**	SS	0
	SP	0.9762
	PPV	0
	NPV	0.9762
	LR+	0

**Abbreviations for Supplementary Tables S3,S4,S5 and S6**
**AR:** Amplitude criterion; **DCS:** Direct Cortical Stimulation; **i:**ipsilateral**;** **LAT:** Latency criterion; **LR+:**Positive Likelihood Ratio; **MEP:** Motor Evoked Potential **; MT:** Motor Threshold/Mapping criterion; **NaN:** Not available ; **NPV:** Negative Predictive Value; **PPV:** Positive Predictive Value **; PW:** Pulse Width; **SS:** Sensitivity**; SP**: Specificity ; **TES:** Transcranial Electrical Stimulation; **TH:** Threshold criterion

Supplementary Table S7: The table summarizes the relative rates of all motor deficits as well as the total number of early-transient, transient and permanent motor deficits in conjunction with MEP changes.

Authors	No of all new motor deficits/ Total no of patients(%)	No of early-transient motor deficits/ No of all MEP changes(%)	No of transient motor deficits/ No of all MEP changes(%)	No of permanent motor deficits/ No of all MEP changes(%)
**TUMORS AND OTHER BRAIN LESIONS**				
Giampiccolo et al.(2021)	**50%**	**12%**	**23%**	**38%**
	**17%**	**14%**	**21%**	**21%**
Gogos et al.(2020)	**10%**	**N/A**	**33%**	**17%**
Mammadk-hanli et al. (2020)	**N/A**	**N/A**	**N/A**	**N/A**
Abboud et al.(2019)	**35%**	**18%**	**18%**	**22%**
Majchrzak et al.(2018)	**51%**	**N/A**	**39%**	**61%**
Moiyadi et al.(2018)	**18%**	**0%**	**33%**	**0%**
Plans et al. (2017)	**20%**	**19%**	**0%**	**38%**
Zhou et al.(2017)	**14%**	**N/A**	**83%**	**17%**
Abboud et al.(2016)	**13%**	**N/A**	**62%**	**38%**
Boex et al.(2016)	**21%**	**31%**	**19%**	**50%**
Obermueller et al.(2015)	**27%**	**N/A**	**15%**	**13%**

Shiban et al.(2015)	**38%**	**N/A**	**67%**	**33%**
Lee et al. (2014)	**17%**	**N/A**	**0%**	**43%**
Gempt et al.(2013)	**33%**	**N/A**	**33%**	**48%**
Ostrý et al. (2013)	**32%**	**N/A**	**67%**	**0%**
Pastor et al.(2013)	**27%**	**N/A**	**33%**	**25%**
Seidel et al.(2013)	**30%**	**23%**	**20%**	**17%**
Sakurada et al.(2012)	**23%**	**N/A**	**25%**	**50%**
Senft et al.(2012)	**20%**	**N/A**	**57%**	**29%**
Hatiboglu et al.(2010)	**50%**	**N/A**	**N/A**	**N/A**
Ichikawa et al.(2010)	**14%**	**N/A**	**40%**	**20%**
*Szelényi et al.(2010)	**54%**	**30%**	**N/A**	**26%**
Kombos et al.(2009)	**13.3%**	**N/A**	**40%**	**0%**
Neuloh et al.(2009)	**27%**	**N/A**	**37%**	**14%**
Neuloh et al.(2007)	**31%**	**N/A**	**46%**	**20%**
Suess et al.(2006)	**15%**	**N/A**	**13%**	**36%**
*Neuloh et al.(2004)	**42%**	**N/A**	**39%**	**22%**
Kombos et al.(2001)	**11%**	**N/A**	**0%**	**17%**

Zhou et al. (2001)	**16%**	**N/A**	**N/A**	**N/A**
Cedzich et al.(1996)	**36%**	**N/A**	**27%**	**27%**
**EPILEPSY SURGERY**				
Koo et al. (2019)	**0.7%**	**N/A**	**0.7%**	**0%**
Neuloh et al.(2010)	**13%**	**N/A**	**19%**	**16%**
**ANEURYSM CLIPPING**				
Guo et al.(2021)	**11%**	**N/A**	**10%**	**25%**
Park et al.(2021)	**3%**	**N/A**	**25%**	**17%**
You et al.(2021)	**12%**	**N/A**	**N/A**	**N/A**
Kameda et al.(2020)	**5%**	**50%**	**0%**	**0%**
Byoun et al.(2019)	**3%**	**N/A**	**0%**	**40%**
Greve et al.(2019)	**7%**	**N/A**	**8%**	**15%**
Li et al. (2019)	**57%**	**3%**	**23%**	**30%**
Choi et al.(2017)	**2%**	**N/A**	**10%**	**20%**
Komatsu et al. (2017)	**0%**	**0%**	**0%**	**0%**
Staarman et al.(2017)	**2%**	**20%**	**N/A**	**N/A**
Kim et al.(2016)	**5%**	**N/A**	**N/A**	**N/A**

Maruta et al.(2016)	**7%**	**N/A**	**7%**	**10%**
Song et al.(2015)	**27%**	**50%**	**N/A**	**N/A**
Sasaki et al.(2014)	**3%**	**N/A**	**5%**	**9%**
Takebayashi et al. (2014)	**20%**	**N/A**	**32%**	**21%**
Yue et al.(2014)	**9%**	**7%**	**0%**	**13%**
*Dengler et al.(2013)	**6%**	**N/A**	**7%**	**7%**
Kang et al.(2013)	**8%**	**38%**	**N/A**	**N/A**
Maruta et al.(2012)	**14%**	**N/A**	**50%**	**0%**
Shi et al.(2012)	**N/A**	**N/A**	**0%**	**33%**
Motoyama et al. (2011)	**2%**	**20%**	**N/A**	**N/A**
Irie et al. (2010)	**7%**	**33%**	**N/A**	**N/A**
Yeon et al.(2010)	**1%**	**N/A**	**8%**	**0%**
*Szelényi et al.(2007)	**5%**	**N/A**	**14%**	**9%**
Weinzierl et al.(2007)	**0%**	**N/A**	**0%**	**0%**
Szelényi et al.(2006)	**13%**	**N/A**	**17%**	**50%**
Horiuchi et al.(2005)	**8%**	**N/A**	**30%**	**10%**
Suzuki et al. (2003)	**5%**	**N/A**	**20%**	**5%**

ENDOVASCULAR PROCEDURES FOR ANEURYSMS				
Nakagawa et al.(2020)	**6%**	**43%**	**N/A**	**N/A**
Wilent et al.(2020)	**2%**	**24%**	**N/A**	**N/A**
Lee et al.(2019)	**0.7%**	**14%**	**N/A**	**N/A**
Piñeiro et al.(2015)	**38%**	**N/A**	**0%**	**50%**
Hiraishi et al.(2011)	**14%**	**33%**	**N/A**	**N/A**

Supplementary Table S8: Causes of injury in tumor patients with permanent motor deficits

**Authors**		**Mechanical Injury**	**Vascular injury**	**Unclear nature of the injury**
Gogos et al.(2020)		-	2/58 (3.4%)[1)]	-
Seidel et al.(2020)		3/182 (1.6%)	3/182 (1.6%)	-
Plans et al. (2017)		-	1/92 (1.1%)	13/92(14.1%)
Boex et al.(2016)		-	5/104 (4.8%)	-
Obermueller et al.(2015)	Glioma	4/105 (3.8%)	1/105 (1%)	-
	Metastasis	1/53 (1.9%)	1/53 (1.9%)	-
Shiban et al.(2015)		-	1/14 (7.1%)	-
Lee et al. (2014)		-	3/84 (3.6%)[2)]	-
Gempt et al.(2013)		-	8/70 (11.4%)	2/70 (2.9%)
Seidel et al.(2013)		3/100(3%)	2/100(2%)	-
Sakurada et al.(2012)		-	1/30 (3.3%)	-
Hatiboglu et al.(2010)		-	1/16 (6.3%)	2/16 (12.5%)
Ichikawa et al.(2010)		-	1/21 (4.8%)	-
Neuloh et al.(2010)		3/86 (3.5%)	-	-
Szelényi et al.(2010)		15/25 (60%)	4/25 (16%)	-

Neuloh et al.(2009)	-	2/191(1%)	2/191(1%)
Neuloh et al.(2007)	-	7/88(8%)	-
Suess et al.(2006)	17/232 (7.3%)	2/232 (0.9%)	-

Notes:

1)The authors also report about previous series with almost equal distribution of vascular and mechanical injuries (in this previous series they applied Penfield stimulation without MEP monitoring and without monopolar short train mapping)
2) The authors stated they could only speculate on the cause

In the remaining 14 included tumor papers no specification about the type of injury that led to permanent motor deficit was described
